# Supplementary material for: Leadership and governance for integrating mental healthcare at the primary healthcare (PHC) level: A mixed methods study in Ghana
Source: PLOS Glob Public Health. 2025 Aug 19;5(8):e0002672. doi: 10.1371/journal.pgph.0002672 (PMC12364371; doi:10.1371/journal.pgph.0002672)
Supplement: S1 Text — (DOCX) [file pgph.0002672.s001.docx]

**Results/thematic illustration of the actor mapping exercise**

### **Data collection and analysis framework**

The table below provides a framework of the data collection approaches that was be applied to collect data from among the study respondents and the analyses field data

Table A: Research study data collection and analysis framework

| **Research objectives** | **Study design** | **Population/respondents** | **Sampling technique & sample size** | **Data collection methods** | **Dependent/independent variables (indicators)** | **Data analysis** |
| --- | --- | --- | --- | --- | --- | --- |
| Socio-demographic characteristics of study respondents. | Descriptive | - District Health Management Team (DHMT) Regional Health Manager Team (RHMT) officials through survey & KIIs/FGDs - Community Psychiatric Nurses (CPNs)/ Community Mental Health Officials (CMHOs) - Other healthcare service providers are the PHC/CHPS level - User of mental Healthcare services/ persons living mental health conditions - Primary caregivers of people with mental health conditions - Funders/ Development Partners | **Quantitative:** Stratified purposive; sample 1104  **Qualitative:** Purposive, ensuring maximum variation; 27 KIIs/12 FGDs Saturation attained | Survey/ FGD/KII | - **Sex:** Male / Female - **Age:** 18-27/ 38-39/40-40/50-59/ 60-above - **Education:** No education/ Basic/ Secondary/ Tertiary - Profession: - Occupational: - **Other characteristics** - User of mental healthcare services - Caregivers of people with mental health conditions - Community Mental Healthcare service providers - District/ Regional & Hospital/ Health-facility directors - National level health policy official | Descriptive   - **Quantitative:** Survey questionnaire (frequency/ histograms/ proportions/ charts, graphs)   **Qualitative:** Key Informant Interview (KIIs) & Focus Group Discussions (FGDs) |
| - 1. To describe the range of mental health care services delivered at the PHC level in Ghana. | Descriptive | - District Health Management Team (DHMT) Regional Health Manager Team (RHMT) officials through survey & KIIs/FGDs - Community Psychiatric Nurses (CPNs)/ Community Mental Health Officials (CMHOs) - Other healthcare service providers are the PHC/CHPS level - User of mental Healthcare services/ persons living mental health conditions - Primary caregivers of people with mental health conditions | **Quantitative:** Stratified purposive; sample 1104  **Qualitative:** Purposive, ensuring maximum variation; 27 KIIs/12 FGDs Saturation attained | Survey, FGD/ KII | The package or range of available mental healthcare services delivered at the Primary Health Care level in Ghana   - Essential mental health services provided at the Primary Health Care PHC/CHPS level (diagnosis, prescriptions, medicine refill, mental health education. in-patient and referrals) - Service organisation and stakeholder coordination in mental healthcare delivery the PHC/CHPS level - Accessibility of mental healthcare services at the community level   Level of integration of mental healthcare into general healthcare services delivered at the PHC/CHPS | Descriptive:   - **Quantitative:** Survey questionnaire (frequency/ histogram proportions/ charts, graphs) using stataSE16   **Qualitative:** KIIs & FGDs) thematic analysis (Braun & Clark) using NVivo12 |
| - 1. To determine availability of resources for mental health care at the PHC level (numbers and mix of personnel, funding/budgets, and essential medicines, and information, including research, for management decision-making, research, and partnerships) | Descriptive | - District Health Management Team (DHMT) Regional Health Manager Team (RHMT) officials through survey & KIIs/FGDs - Community Psychiatric Nurses (CPNs)/ Community Mental Health Officials (CMHOs) - Other healthcare service providers are the PHC/CHPS level - User of mental Healthcare services/ persons living mental health conditions - Primary caregivers of people with mental health conditions - Civil society advocates - Donor community/ development partners | **Quantitative:** Stratified purposive; sample 1104  **Qualitative:** Purposive, ensuring maximum variation; 27 KIIs/12 FGDs Saturation attained | Survey, FGD/ KII | Resources supporting integration of mental healthcare services (personnel, funding, medicines)   - Number and mix of mental healthcare personnel delivering services at PHC/CHPS compared to optimum mix of personnel required - Adequacy of mental healthcare personnel at the PHC/community level - Sources of funding for mental healthcare service provision at the PHC/ community level - Funding allocations for mental healthcare at the PHC/CHPS - Expenditure on mental health at the PHC/ community level - Individual/ household out-of-pocket expenditure on mental health at the PHC/ community level - Psychotropic medicines in the list of essential medicines at PHC/CHPS level - Availability of psychotropic medicines at the PHC/ community level | - Descriptive: - **Quantitative:** Survey questionnaire (frequency/ histogram proportions/ charts, graphs) - **Qualitative:** KIIs & FGDs) - **Document review:** GHS/MHA annual reports / Ministry of Health (MoH) National Health Accounts (NHA) |
| - 1. To examine the level of community participation and ownership in the integration of mental healthcare at the primary health care level | Descriptive | - District Health Management Team (DHMT) Regional Health Manager Team (RHMT) officials through survey & KIIs/FGDs - Community Psychiatric Nurses (CPNs)/ Community Mental Health Officials (CMHOs) - Other healthcare service providers are the PHC/CHPS level - User of mental Healthcare services/ persons living mental health conditions - Primary caregivers of people with mental health conditions - Civil society advocates | **Quantitative:** Stratified purposive; sample 1104  **Qualitative:** Purposive, ensuring maximum variation; 27 KIIs/12 FGDs Saturation attained | Survey, FGD/ KII | Level of involvement of individuals/ families and communities in mental health service organisation and delivery:   - Sense of consultation of individuals/ families and communities in mental health service organisation and delivery - Sense that the services provided reflect/ address the felt needs of individuals with mental health conditions - Sense of empowerment of families and communities to enhance mental health literacy/ health promotion   Participation of private sector in mental healthcare delivery at the community level | - **Quantitative:** Survey questionnaire (frequency/ histogram proportions/ charts, graphs) using stataSE16 - **Qualitative:** KIIs & FGDs) thematic analysis (Braun & Clark) using NVivo12 |
| - 1. To examine leadership and governance structures for mental health care at the PHC level in Ghana | Descriptive | - User of mental healthcare services - Caregivers of people with mental health conditions - Community Mental Healthcare service providers - District/ Regional & Hospital/ Health-facility directors - National level health policy official | **Quantitative:** Stratified purposive; sample 1104  **Qualitative:** Purposive, ensuring maximum variation; 27 KIIs/12 FGDs Saturation attained | Survey, FGD/ KII | Effectiveness of mental health leadership and governance at the Primary Health Care (PHC)/ Community Health Planning and Services (CHPS) level  Focus is on the *results-based indicators* of the Leadership & governance block   - Effectiveness of implementation of Mental Health Act & Policy: - District Mental Health Plan - Focal person/ Coordinator for mental healthcare services at the PHC/CHPS level - Presence of a Mental Health Unit at PHC/CHPS level - Effectiveness of reporting lines - How involved are mental health staff at the community level in mental healthcare service organisation and delivery. - Policy environment for private sector participation - Effectiveness of monitoring & evaluation of implementation of mental health legislation and policy | - **Quantitative:** Survey questionnaire (frequency/ histogram proportions/ charts, graphs) using stataSE16 - **Qualitative:** KIIs & FGDs) thematic analysis (Braun & Clark) using NVivo12 - **Document review:** Mental Health Act and policy/ strategic plan(s); relevant reports of the Ghana Health Service (GHS) and Mental Health Authority of Ghana (MHA) |
| - 1. To explore stakeholders’ perspectives of the level of integration of mental health care at the PHC level in Ghana | Descriptive | - District Health Management Team (DHMT) Regional Health Manager Team (RHMT) officials through survey & KIIs/FGDs - Community Psychiatric Nurses (CPNs)/ Community Mental Health Officials (CMHOs) - Other healthcare service providers are the PHC/CHPS level - User of mental Healthcare services/ persons living mental health conditions - Primary caregivers of people with mental health conditions   Civil society advocates | **Quantitative:** Stratified purposive; sample 1104  **Qualitative:** Purposive, ensuring maximum variation; 27 KIIs/12 FGDs Saturation attained | Survey, FGD/ KII | Stakeholder impressions/ views of the level of integration of mental health at the Primary Health Care level, factors affecting effective integration of mental   - Stakeholder scores gauging perceptions of integration - Mental healthcare integration performance assessments carried out - Utilisation of community level information for mental healthcare development - Research conducted | Descriptive:   - **Quantitative:** Survey questionnaire (frequency/ histogram proportions/ charts, graphs) - **Qualitative:** KIIs & FGDs)   **Document review:** GHS/MHA annual reports |

**Key Informant Interview (KII) of xxxxxxxxxxxxxxxxxxxx**

| **Name of facilitator** | xxxxxxxx |
| --- | --- |
| **Name of note taker** | xxxxxxxxx |
| **Type of respondent** | Psychiatrist; Ghana Health Service (GHS) |
| **Gender of respondent(s)** | Female |
| **Disability of respondent(s) (physical or psychosocial)** | None |
| **Age of respondent(s)** | Not Provided/ Not asked for |
| **Community Name** | Bolgatanga (via telephone interview)  Telephone recorded interview. The respondent is a national level health policy & service implementation individual. |
| **Interview date** | 8^th^ August 2020 |
| **Start Time** | 10:05am |
| **End time** | 11:39am |
| **Recorder number** | Phone_20200808-100536__233242688206 |
| **Name of file (follow convention below)** | Transcript_Key Informant Interview_xxx-xxxxxxxxxxxxxxxxxxxxxxxxxxxxxxx.docx |

**Introduction**

xxxxxxxxxxxxxxxxxxxxxxxxxxxxxxxxx.

**Interviewer**

Once again, good morning and thank you for the opportunity, I am undertaking a study titled ‘integration of mental health care at the community level in Ghana, the role of the health systems framework’.

**Respondent**

ok

**Interviewer**

Yes, as part of contributing to knowledge and possibly erhm influencing community mental health service development. So, I will like to ask you to kindly introduce yourself, your full name, what you do, and probably your role or what your full-time work is.

**Respondent**

Ok. So, my name is xxxxxxxxxxxxxxxxxxxxxxxx. I’m a psychiatrist and I am currently the focal person for mental health xxxxxxxxxxxxxxx. So, essentially, my role is to ensure proper integration of mental health services in the xxxxxxxxxxxxxx. So, what we are looking at is integrating mental health in[to] Primary Health Care. This very important because currently, we have just one psychiatrist in the Ghana Health Service which is just xxxxxxxxxx and we don’t have the specialised people to be able to offer [mental health] services but we are all aware that a lot of the people that come into Primary Health Care [facilities] have identifiable mental health conditions that must be addressed if we are looking at giving people holistic care. So that is what we are looking at. Being able to build the capacity of Primary Health Care workers to be able to give mental health care [services] as part of the care [services] we give [to] people in Primary Health Care. So that is by way of introduction.

**Interviewer**

Ok. Thank you so much. So, I want to get your views and perspectives on mental health service organisation and coordination, as well as access [to mental health services] at the community level. What mental health services are available [at the community level] in /[for] treating mental health conditions in the community?

**Respondent**

What mental health services are available?

**Interviewer**

Yes. At the community level.

**Respondent**

At the community?

**Interviewer**

Yes.

**Respondent**

Ehm, I am not too sure if I understand the question. Can you break it down for me?

**Interviewer**

Are mental health services provided at the community level and what are they?

**Respondent**

Ok, yes. Mental health services are provided in the community. So, when we talk about community, what exactly are we looking at? Erhm, I am looking at community as the primary health care, ok. So currently, in every place in Ghana, at least, you can have, you have [a] health facility, I mean the various levels of health facilities in all the communities around Ghana, and in each of these places or very close to them, at least we know that we have professionals who are providing services. So, we have a community psychiatric nurse. We have community mental health workers/ officers. We have community mental health officers. At least these people are able to provide some [mental health] services. So, for the community mental health services, what we know is that they are trained to identify the cases, but they do not manage them. They rather refer them to the units. But the Community Psychiatric Nurses do manage the cases as part of their programme, they do community durbars and they do health talks in various places – school visits. We run a lot of programmes to be able to get the people to understand that they are around and that these services are available to them. They see the cases, manage them, and then do follow ups. And then when they think that the cases are beyond them, they refer to the higher level for further management. So that’s what we do. In this regard, I know the mental health services delivered at the Primary Health care facilities, even though limited in nature, they meet minimum standard and generally meet the felt needs of clients [mental health service user].

**Interviewer**

Yes, thank you very much. I think I have heard you well. I’ll like you to, may be, break it to the specifics. When you talk about they manage the cases, I think I am hearing you talk about mental health promotion and treatment. But what is exactly involved in the management?

**Respondent**

What exactly is involved in the management?

**Interviewer**

Yes

**Respondent**

So, if you look at mental health, causes of mental health [conditions], you are looking at the bio-psychosocial model. So, you are looking at the biological factors, then psychosocial factors. Essentially when we want to be able to give proper health for [to] our clients. Even if you approach them with this three-pronged approach – bio-psychosocial. So that is what our people are doing like we expect to do. So biologically, they are giving them medication and they are prescribing [to] medications. And then psychosocial, they address whatever underlying social stresses are. They identify them and then help the people manage them. And if they need to refer them to some other quarters they do that.

**Interviewer**

Ok, so the, all these will involve doing diagnoses and providing prescriptions, and reviewing progress of treatment of persons?

**Respondent**

Yes

**Interviewer**

I guess the health promotion activities you talked about also have something to do with prevention?

**Respondent**

Yes

**Interviewer**

Ok, can you say more about that?

**Respondent**

Erhm, so what they do is that, like I said, they have the durbars that they organise. They go into the schools. So, they have a programme they refer to as, is it, school health, or something like that.

**Interviewer**

Ok

**Respondent**

So, they go into the schools. They talk about mental health in general and then they talk about causes [of mental illnesses], risk factors as in you can talk about. So, risk factors. They talk to the people on, you know, being able to identify. So, they tell them [the] early signs and symptoms, how to identify if somebody has such a problem. And then, they, erhm, teach them as to, so after you have identified how would you be able to help the person. So, a lot of the time they refer them to these mental health professionals in the communities. So, they refer them to the mental health unit and they take it up from there. So, they do their clerking, they make the diagnosis, start treatment, if they need to bring in relevant others, as in family, friends, or opinion leaders, whoever they think is relevant in the management of the case then they do that.

**Interviewer**

Ok, thank you very much. So being in this role as the national focal person for mental health at the Ghana Health Service, how accessible are mental health services that are provided at the community level?

I am trying to understand how accessible mental health services in the communities.

**Respondent**

How accessible are these [mental health] services? Well, there is always more room for improvement, yes, there is more room for improvement, but I think that in every district in every district hospital, yes, ok, so, that I am certain about, in every district hospital you have mental health units. But I know that in almost every health facility we have community psychiatric nurses in almost every facility. Even if you don’t have in a particular facility not far away from that facility you will have community psychiatric nurses around. So, I think that fairly, mental health [services] is [are] accessible to the people but the question is the quality of the service. So, that is what I am really concerned about. Yes, the people are there and I must say they are really working. I go round, I mean, [and] places other health workers will not want to go these psychiatric nurses are always ready, they work with so much passion and all that and go all out to care for their patients or their clients but my concern has always been what is the quality of the service that is being provided because we know that these people are trained as nurses, ok, so, these are not people we have trained specifically to be able to identify and to manage the cases as they are doing now. But erhm, requisite number, we can’t even talk of requisite number because it is none-existent. So, we don’t even have specialists who are supposed to be doing this, yet when we train medical officers or the other clinicians it is expected that everybody goes through some form of training to be able to identify the cases in mental health. But the question is, even without training how much of mental health are we doing and how seriously are we taking mental health to the extent that once people step out of medical school or out of whatever training they had they just forget about mental health. But for the psychiatric nurse, we know this is my field and this is what I have decided to do so we have had to fall on these people who are ready to do the work. So, that task has been added to theirs which is rather unfortunate but under the current circumstance I think that that was the one way we could handle the situation. So, even though traditionally, they are not expected to make diagnosis and manage, the circumstance under which we find ourselves has forced us to shift that task to them. So, yes, mental health service is available accessible but the quality needs to be improved.

**Interviewer**

Ok. So, that brings me to the question [that] I want your expert dilation on. What are the services that should be in every health facility right from the community level to the tertiary level, if you can tell me?

**Respondent**

Peter can you please help me.

**Interviewer**

Yes. So, I want to understand the levels of service provision from the primary up to the tertiary level. at each stage or at each level what kind of services are expected to be delivered?

**Respondent**

You mean the ideal situation?

**Interviewer**

Yes, the ideal situation.

**Respondent**

For the ideal situation, well if you look at the WHO optimal mix of service for mental health you will realise that at the base, if you look at the pyramid, you have self-care at the base. On top of self-care you have informal community care and above it is mental health services in primary health care and I think at the apex is where you have the tertiary psychiatric institutions. Am I right? Help me.

**Interviewer**

Yes, well, I want to hear your perspectives. It’s not a must, I mean, whatever you understand it. Yes.

**Respondent**

Ok. So, and then if you look at the pyramid, in terms of the cost for service delivery. You realise that as it goes up it becomes very, very expensive. Again, when it comes to the number of people that have access to [menta] health [services]. Even though at the apex it is the most expensive, at the apex you realise that only a few people have access to the [mental] health services that they need. Ok. So, for me, I think that we should rather concentrate on building the lower level. If we build the service at the lower level then you have a lot more people having access to quality [mental] health care. That is what I think the ideal situation should be. We are looking at improving access to quality [menta] health care [services]. So, if we talk about access to quality [mental] health care what are we looking at? So, as I said, in every community at least you will find a health post. So, who are the people providing services at the health post? We have physician assistants, we have general practitioners, medical officers. Can we, you know, depend on these people to be able to provide the mental health services we think everybody should have access to? My answer is yes. We can depend on them but we are not there yet because we need to give them the requisite knowledge and skills and then the resources to be able to take care of the people. So, when people are sick they go into the hospital, they are seen by these people but currently when people are sick [and] when they have a mental health condition and even when they go into the same space people do not want to attend to them because they think that it is a specialised area and you have a special group people who should see them. So, we have our mental health unit and these units are being manned by the nurses and I am saying again [that] not that we don’t appreciate the work they do but we think that we should be able to build the capacity of the clinicians, the physician assistants, the medical officers to be able to identify the people because, like I said already, these nurses are not trained to be able to make a diagnosis. And when you look at mental health conditions, most of the times [the] people have other co-morbid, you know, physical conditions. A nurse is not trained to be able to, you know, identify these to make a differential diagnosis to be able to manage these cases very well. So, I think that these people must first be seen by doctors, by the physician assistants who are trained to make a diagnosis, roll out other cases where then they start the management. We know the have a lot in their hands. When they start the management then our people the psychiatric units/ nurses can continue and do the follow up and then we expect that the doctors and the physician assistants should also be able to come back and review the cases once in a while. And then we are looking at other forms of psychosocial support. Can we bring on board the people that will be able to help us give us such services, who are these people? So, we can think about the opinion leaders in the community. We can think about [the] social welfare [department], we look at family members because we are with the people. So, in the schools, can we have teachers interested in mental health given the knowledge [and] the skills needed to identify the cases because if have a child that has a mental or neurological problem because people do not even know that there’s a problem a lot of times what happens [is that] we do not even want them in our schools because they become a problem. They are a bit difficult to manage so our teachers and headteachers even the church, our religious leaders let me put it that way, our religious leaders we need to bring everybody on board so that as we are managing, may be biologically, then you are also able to get the psychosocial support that they need. And of course, I know that there are other support groups that are existing in some areas and then there a lot of NGOs that help in that regard. I know that BasicNeeds for instance, you people. BasicNeeds is really doing a lot in terms of that, helping people with psychosocial support. So, I think generally, I have answered your questions or you help clarify. I think we need everybody on board. We need to make sure that when people get into these facilities in the communities, they are getting quality healthcare, holistic healthcare and not just leaving them for the community mental health officers them and the community psychiatric nurses to attend to. So, that is what I think the picture should be like. And when they have managed these cases at the community level and things are complicated they can then refer to the higher institution. So, if I am talking about higher institutions, I really wish we get to a point where we have psychiatrists or other mental health professional or specialist in all the regional hospitals. It is my wish that we get there. We have these people in these regions. So, if the primary health care people are not able to manage, they can refer. And then looking at the mental health law, it says that all regional hospitals should have psychiatric wings but how many regional hospitals have that. And we are saying that the district hospitals should have, at least, a virtual bed to take care of people with mental, neurological and substance use disorders. How practical is this? Is it even in existence? So, but even if these were available some conditions are such that you need to admit the people for a long time so you cannot put somebody on these beds for a period like three months. Really, one month? Because you need to take care of a lot of people. So, for people that have cases that need to be admitted for a long time or a longer period, these one you can refer to the three tertiary psychiatric institutions so that they do the specialised care. But then, quality [mental] healthcare should trickle down to everybody at the community level. Yes, that is what I think.

**Interviewer**

Thanks so much. I think you’ve said so much and that’s helpful. Erhm, I will come back to the issue of psychiatrists or what kind of personnel should be across [the] levels. Yes. But just to confirm I mean, we talked about the ideal situation, the WHO pyramid, but in Ghana is there a document, is there whatever written up to show the levels of mental health service provision across the health system right from primary to secondary to tertiary level. Do we have a document of that nature somewhere?

**Respondent**

Erhm, Peter, I must say that I am not aware of any such document, but I think it will be good for me to find out. So, I will note that and then find out. In fact, I am not aware of such [document].

**Interviewer**

Ok

**Respondent**

Is there anything like that you are aware of?

**Interviewer**

I have no idea actually. That is why I am doing this study.

**Respondent**

Ah ok. I am not a of such

**Interviewer**

Yes. Thank you. So, I want to come to the issues of human resources. Tell me about mental health workers in community health facilities providing mental health services. If you know their number, it may not be [possible] but if you know their number and gender mix.

**Respondent**

Oh, I will need to [check]

**Interviewer**

You will need to [refer] but what are the calibre [of personnel], I think you mentioned some CPNs and community health workers.

**Respondent**

I am trying to look for some documentation in my laptop [computer]. So, erhm, I’m coming.

**Interviewer**

Yes

**Respondent**

We can talk about something else while I look for it.

**Interviewer**

Ok

**Respondent**

Because, I don’t want to say something that I am not sure of. It will be better for me to speak to the issues. If I don’t know, I don’t know that say something that is not true.

**Interviewer**

Yes. No problem. But from the top of your head what are the mix of health [care] service providers providing mental health services at the community level?

**Respondent**

At the community level, we have the psychiatric nurses, the community mental health officers, then there’s the community volunteers but those people they are not really trained, they are just volunteers. These are the one that on top of my head I can [mention] looking at... yes.

**Interviewer**

Ok, alright and how are mental health professionals working at the community level accredited?

**Respondent**

Ok, a hah, so, I think that there’s also the CPOs. The CPOs too are there. When you talked about accreditation, I remembered that they are the ones that we have an issue with now. The CPOs.

**Interviewer**

Ok, who are the CPOs? What’s the full name?

**Respondent**

They are Community Psychiatric Officers. So, these are physician assistants that have done an additional course in mental health. So, they have this small training on mental health. Yes, so that is what it is, that is basically it. They are trained as physician assistants but they just have, how do you call it, an additional training in mental health service.

**Interviewer**

Ok. So, that is, the CPOs, the CPNs and which other ones [that] you also mentioned.

**Respondent**

And the Community Mental Health Officers. These ones they are trained in Kintampo. They are trained to identify cases in the community and refer. Basically, that’s what they do. And then erhm, for the volunteers, these ones, they are not in any particular specialised group. they are just community people trained to identify mental health presentations so that they can be able to refer them to the appropriate quarters for treatment.

**Interviewer**

Ok, thank you. So, erhm, back to the ideal or what should be in place. In every level what calibre of staff should be place for us? The maximum level or the mix of mental health staff that should be at every level?

**Respondent**

At every level?

**Interviewer**

Yes. Ok, let’s take the community level. At the community level, in the ideal situation what [is] are the mix of mental health team or [mental health] service provider team that should be at a community mental health facility?

**Respondent**

Community mental health facility

**Interviewer**

Yes

**Respondent**

You mean we are creating a different facility?

**Interviewer**

In a typical community mental health facility what mix of staff should be available if we had everything?

**Respondent**

E-heh, but right now we don’t even want to create a community mental health facility. Because we are looking at integration. Putting together with all the others in primary health care.

**Interviewer**

Ok, may be my mention of facility has got it wrong. In a typical community level mental health [service] provision, what mix of personnel should be there to provide mental health services? If we had everything.

**Respondent**

If we had everything. So here too, I need you to help me. If you say community, what kind of facility are you thinking about?

**Interviewer**

That’s why I have dropped the name ‘facility’. In providing mental health services at the community level, what mix of staff or personnel should be available to provide mental health services? For example, if you have a cardio unit you will have their mix of staff. If you have the ophthalmology they will have their [mix of staff] so in a typical community mental health unit at the primary level, what mix of staff should be there?

**Respondent**

Ok, I get it. Oh, so, typically, I will be happy to see a psychiatrist, but that will be in like 50 years [time]. I cannot dream that one.

**Interviewer**

Yes, I think I want to understand. As I said, you did mention [that] access is ok [but] quality is a problem and part of it could be personnel. I may be wrong. So, I want to understand, in service organisation, in a mental health organisation at the community level, if we had everything what mix of personnel should we have.

**Respondent**

Ok, at the community level we should have a physician, a doctor or physician assistant, ok who would be able to identify and manage these cases. I will be expecting to see psychiatric nurses there. I’ll be expecting to see a clinical psychologist, a social worker. We do not have [an] occupational therapist but it will be good to have an occupational therapist will be good. And what else, erhm, Peter this is what I can think about.

**Interviewer**

No problem. Thank you very much. So, to close that part of it. Do we have a written up documents that spells out our mix of staff at every level of mental health service provision, particularly at the community level?

**Respondent**

Peter, I don’t know of any such [document]. I don’t know.

**Interviewer**

Ok, that’s fine.

**Respondent**

I don’t know but again I’ll have to find out.

**Interviewer**

Yes, please. No problem. Yah.

So, I want to go on to the mental health legislation. We have a mental health law that I very much know. Do we [you] see the law promoting delivery of mental health care at the community level and if so why?

**Respondent**

Waw, do we see?

**Interviewer**

And if not why?

Yes.

Does the law as it is promote community or mental health care service provision at the community primary level and why?

**Respondent**

Peter, you know what?

**Interviewer**

No

**Respondent**

You should have told me to read the law. (*respondent laughs)*. This is becoming serious business.

**Interviewer**

*(laughs along adding that)* I thought it would have been one of your everyday books that you have.

**Respondent**

Oh, but even with that one, if I know I am going to speak to it. Because, I know where this is leading to. Then I revise my notes well and speak to it very well.

**Interviewer**

Ok

**Respondent**

Because, I don’t want to, you know. So, you give me time. Let me go, may be not the questions, let me through it again. You know when you haven’t really thought about something and your are reading the document it’s different but if you think about it and you go looking for it specifically, it might also be different. So, you let me note the issues concerning the mental health law that you want me to clarify. Because me, there is one thing I believe in. When you have opportunity to speak, you speak to facts but not whatever you are thinking. It’s not that you have the platform so you just say anything. So please, give some time, let me note down the issues [and] go look them up because as I sit here I have my own views but let me go and then look at it again.

**Interviewer**

Ok, so let me

**Respondent**

So, now you want to know if the Act promotes mental health provision at the primary level.

**Interviewer**

Yes. Does the Act promote mental healthcare provision at the primary level and if so why or how? And Doc, you know you are a very busy person [and] I do hope you would send something but let me rephrase the question. Should any mental health legislation be focusing on mental health provision at the primary level and why?

**Respondent**

Certainly!

**Interviewer**

And why?

**Respondent**

Certainly! Because like I explained, if you look at the pyramid of health care, especially for mental health, there we know [that] when you look at the provision of, how do you call it, psychiatric services. As soon as you mention psychiatric services in Ghana, everybody thinks about Pantang [Psychiatric Hospital], Accra Psychiatric Hospital, Ankaful [Psychiatric Hospital]. But these are just three institutions. These are just three health facilities and all of them are in the south [of Ghana]. Now, Ghana didn’t have a lot of psychiatrists and even now, we still don’t have a lot. But let’s say that our number is, you know, we are hitting about 40 [psychiatrists]. If you ask yourself, of the 40 people, psychiatrist we have in Ghana. So, when I’m talking I talk about 40 [psychiatrists]. I have added even those on retirement because once in a while we call some of them to help. They are still active even though they are supposed to be on retirement. So, let’s put it at 39 - 40 [psychiatrists]. Ask yourself, all these 39-40 people [psychiatrists] are in Accra [and] Kumasi. That is the sad situation. all these people are in Accra and Kumasi. I do know that in Cape Coast, Ankaful, we have 2 and then there is 1 in [the] Volta Region. There are 16 regions in Ghana, but all these people are in just these two regions. Now, if all these people are in the two regions, who provides [mental health] care in the other [remaining] regions. That is why we need to go down and equip people who are already in the regions to be able to provide the [mental health] care that we need to give to the people.

So that when you have equipped these people with the knowledge and skills and resources to be able to provide the mental health care at that level. For the complicated cases, then we can refer to these higher or three institutions. But you even ask yourself, these institutions, how many cases can they even admit, how many cases can they attend to? So, it is very, very crucial, very important that we build the capacity of the service providers in primary health care. So, I’m looking at doctors, professional officers, I’m looking at physician assistants, then we have nurse prescribers, in mental health [care] delivery we have the nurse prescribers, these are the nurses that have been given [training?] that are able to make a diagnosis and start management. We need to build their capacities. We cannot be talking about the three institutions and talk about mental health. Really! So, I think that we should stop looking at tertiary institutions, the few of them and rather build the capacity of people in primary health care, at the lower level, because the people are with us in our communities. they are with us, I mean, and then they access health care everywhere. So, if the go into the health facilities, they are going there with their malaria, they are going there with their hypertension, their diabetes, why can’t they go the same places with their anxieties, the depression, post-partum cases and all that? I hope I have been able to answer the question now.

**Interviewer**

Yes

**Respondent**

If you need further clarification I can provide.

**Interviewer**

Yes, I can follow up. Thanks. That’s helpful. I want to also focus on [mental] service users. Persons with lived experience. Do you think they have a role in the development of community mental health services and how should they be involved? *This should have been how are they involved in service organisation at the community level?*

**Respondent**

I think they do have a role. So, you, globally, especially in Ghana, mental health comes with lots of stigma and discrimination but we also know that mental health [mental illness] is not respecter of persons. So, everybody, from whatever social class can suffer mental illness. And then in our communities also or in our settings, we ascribe a lot of meaning to mental health or mental health disorders. It will be very important for us to bring in the service users to help us move mental health. If people are in the community and then we see them not doing well mentally and then we are able to manage these cases, we can such these [such] people as ambassadors. So, they can help us talk to other people in the community, you know, about the fact that it affects everybody, including myself, and that help is available. In actual sense, a lot of people do well when they access help [within their community] and comply so that this whole non-appreciated of mental health, mental health is shrouded in some mystery and all that will go away. So, we need to bring in the service users to deal with them as ambassadors for [to] other people. So, if I see that this person was broken down some time ago but is doing well and I can look up to that persons and say I will also seek help, ok. Then these our people who have been in that situation, so even if you are formulating anything it is not as though you just imaging it but these are service users who can best tell you what needs to be done. For us that have never experienced that condition will just assume that oh, this will work well for these people. But if you bring them on board, they can tell you that oh, no, even though you are thinking about it this way, it would have been better if you had thought about this way of if you had done it this way. So, I think that it is very, very important [that] we bring them on board and that than sit somewhere and imagine that we know it all. They will be able to tell you how it is like or what will make life much, much better for them.

**Interviewer**

U-huh! Thank you. This is helpful

**Respondent**

I am still looking for the document

**Interviewer**

No problem, if it possible for it to be e-mailed, that’s ok. But I just wanted to touch on; to go back on this human resource issue.

**Respondent**

That is what I’m saying. I’m still looking for it.

**Interviewer**

Yes. Uhmm, So, who manages mental health care workers at the primary health level and how are they managed?

**Respondent**

Erhm, so we know that mental health services are provided in all the agencies of the Ministry of Health. So, agencies, I am talking about CHAG [Christian Health Association of Ghana], I am talking the about Ghana Health Service, I am talking about the private sector, I am talking about Mental Health Authority [of Ghana]. So, the management in these agencies are the ones that manage or who oversee human resource of mental health [workers]. So if you come to the Ghana Health Service for instance, the heads of the facility are responsible. If you go into CHAG, they are responsible. In the same vein, if you go into the Mental Health Authority facility, heads of the facility are responsible.

**Interviewer**

Ok, Do [does] Mental Health Authority have facilities?

**Respondent**

Oh, yes.

**Interviewer**

Which are those ones?

**Respondent**

The three psychiatric hospitals

**Interviewer**

Oh, ok. Alright.

**Respondent**

But they [Mental Health Authority of Ghana] also have regional mental health coordinators. I know of the 10 regions. For the new ones they are yet to appoint regional mental health coordinators. But the 10 regional mental health coordinators appointed by the Mental Health Authority are attached to the regional health directorates so they work with the regional heath directorates. So, they work with the regional health directorate. So, for us in [the] Ghana Health Service we work with the regional mental health coordinators that are appointed by the Mental Health Authority.

**Interviewer**

Uhuh, ok. How effective is that, those regional mental health coordinators, in terms of their management?

**Respondent**

Erhm, in terms of their management, erhm, well, I think that it’s bit of a challenge. I’m speaking for mental health. I am not speaking of the others. I do know that the regional mental health coordinators are doing a lot of work. So, they work. But our challenge as Ghana Health Service has been the fact that, you know these are people that are appointed by one agency and then we have to depend on them to do some work. So, really at a point, there is a conflict. Who do they owe their allegiance to? So, that has been our major challenge. But apart from that I think that the regional mental health coordinators or some are really, really on the ground and they are working. And then we work with them at thar level but I cannot speak for the other agencies.

**Interviewer**

Mmh, what is the nature of the challenge? Is it to do with insubordination, is it to do with reporting lines?

**Respondent**

It’s basically reporting lines. Because who do I report to? Because the truth is that these people are not appointed by the Ghana Health Service. Ok. So, well sometimes we do complain but I ask myself, if I were in their shoes, won’t I behave the same way? Because, really, I don’t owe you any responsibility. I am appointed by one agency, so, if anything at all, I should be satisfying that one agency. So, what is that one agency that appointed you, what is expected of me by that one agency. May be when that has been defined then probably the other agencies that are falling on me, you know, can draw from. But I’m not too sure what their job description is or what when they were appointed by the Mental Health Authority. Yes, so the Mental Health Authority is just like, is another agency of the Ministry of Health, just as Ghana Health Service. So (*interrupted)*.

**Interviewer**

Ok, can there be a way of improving the coordination of the health worker, the regional coordinator as an example, in the development of community mental health?

**Respondent**

Oh, I think that it is something that is doable. I think it is something that is doable and has to be looked at. That’s why I’m saying that I don’t know their JD, their job descriptions, [provided by] the appointing authority. I don’t know about it so it will be a bit difficult. But if I knew their job description then I could say oh if we did this [in] this way it would make it better. As I sit here, I don’t know what their JD is.

**Interviewer**

Ok, *waw*, thank you.

**Respondent**

And then especially if you are looking at the agencies playing different roles. So, if they are playing different roles then certainly the JDs of coordinator, the regional mental health coordinator would be leaning towards the interest of the appointing agency and not necessarily to those of us at the receiving end.

**Interviewer**

Mhmm, ok. I see. Thanks very much. I know it is almost getting to an hour but I have just two questions on financing, research and *erh* barriers and recommendations. So, financing of mental health. What are the sourcing of funding for mental health at the community level?

**Respondent**

So, at the community level, people are paying out of the pocket. Funds health facilities. Ok, you have donors, NGOs, other groups, And then, well, I was going to say that some facilities also help the mental health unit but it also comes from these same, *erhm* sources – out-of-pocket and then from the donors. *Erh*, we are looking at insurance. So, currently, for people, for example, for people that have epilepsy, epilepsy is a neurological disorder so they can access their medications through the National Health Insurance (Scheme). We are looking at people accessing all medications regarding mental, neurological and substance use disorders using the NHIS. So, for now, that is how it is [on funding of mental health at the community level]

**Interviewer**

Does the Ghana Health Service at the headquarters level provide any funding for mental health services at the community?

**Respondent**

Erhm, not really.

**Interviewer**

Mhmm. Ok

**Respondent**

Not really but I have to fine out.

**Interviewer**

Ok

**Respondent**

I don’t [know]. Not really, what I do know is that we support in terms of capacity building, training and all that but financial support, no. I have to find out if there’s been some [funding support] in the past and what happened.

**Interviewer**

Ok, how about provision of medical products especially medicines and related equipment and devices. *Errh*, how are they planned for, procured and distributed?

**Respondent**

*Errh, hmm,* Peter I don’t have an answer to this one. I have to find out. [laughs]. Let me put this one down. I have to find out. So, how are medical products, procured and distributed for community mental health service provision? Hmm, even though I’m saying [that] I will find out, I can say that I don’t think I will find anything for community level mental health service provision because really, nobody thinks about mental health. Nobody really thinks about mental health even in our procurement of medication, when was the last time [that] government gave psychotropics? So, that’s a major thing. So, at the national level, we don’t really think about mental health. Even, in our planning and procurement, I don’t know, but I don’t think there’s anything that I will find. I don’t think I will find anything. Even, if I did, it will be very, very, very minimal but I will still go through and then check because I don’t know.

**Interviewer**

Ok. Then knowledge and information flow or feedback, is there an information sharing or feedback process in mental health service provision at the community level?

**Respondent**

I don’t think so. Information sharing and feedback at the community level.

**Interviewer**

Yes,

**Respondent**

So, can you please, expatiate on it for me.

**Interviewer**

Can I ?

**Respondent**

Expatiate, I mean explain it further?

**Interviewer**

Yah, in terms of relaying information or directives and also collecting feedback. I don’t know how do they call it [but] like people will have suggestion boxes and people will write [their views and suggestions and drop them inside the boxes] or your organise specific reviews to hear from service users, service providers and what is going on and how that can inform [the] development of the mental health services at the community level?

**Respondent**

Mhmm, I don’t think there is anything like that, but I still will find out so, I am noting the questions down. I will find out. But I don’t think there’s anything like that. So, may be there’s something that I am not aware of.

**Interviewer**

Ok.

**Respondent**

Yes.

**Interviewer**

How about intentional research to build information and knowledge to develop community mental health.

**Respondent**

None existent.

**Interviewer**

Ok, then information systems. Is there an information system, database used to collect and manage erhm on [mental health] service provision at the [community level] [*interrupted by respondent*]

**Respondent**

Oh, that one, that is the DHIMS. So that’s the DHIMS.

**Interviewer**

So, describe how the monitoring and evaluation processes take place.

**Respondent**

The monitoring and evaluation of the DHIMS or [*injected by interview* or generally]?

**Interviewer**

Yes, generally. Service provision [generally].

**Respondent**

So what happens with this particular one is that, I am sure you know more than me, because I remember recently, a few months ago, I told Dr Sottie that everywhere I pass DHIMS, DHIMS, what is DHIMS? Then she asked me to get the people to tell me and then help me log on to it. So, it’s essentially about data in the district. So, people that have assessed mental health services, so, [with] what conditions they came in. So that, it tells you essentially what is going on in terms of mental health work in the district. So, all the facilities that are providing mental health services. You know, they collect their data and then forward it at the district level, they enter and it is comes up at the national level so you can access it. Apart from that, I don’t know much

**Interviewer**

Thank you very much. To my last bits of questions. I know you have given me more than I deserve.

**Respondent**

Oh, isn’t it because you want to make mental health better for Ghana.

**Interviewer**

Well. Thank you for that.

**Respondent**

We are all looking at making service provision being better because it has to improve. As for that, it has to improve. So, I don’t have a problem.

**Interviewer**

*Mhuh*, yes, and I want to ask this question [that] we all bandy around. You keep hearing integration of mental health services, integration of mental health services whether at the Primary Health Care level of community level, what to your exactly does integration of mental health service mean. I mean by your understanding of it.

**Respondent**

Ok, so, what I think is that everywhere people go to access health [care services], they should be able to access mental health services. So, for instance, if I am not well, for example, if I have hypertension and I go to see the doctor, the doctor or physician assistant, whoever attends to me takes care of my hypertension. When we talk of integration, what we are saying is that, the same places, the same consulting rooms, the same facilities that people go to access health [care services] for all other conditions they should be able to access help for mental health conditions so that I don’t have to go to a separate place a special place to have access to mental health care [services]. Right [from] where everybody goes I should go there to access mental health care and it shouldn’t be a very special person providing care. So, we all sit in the same queue, we all sit in the same OPD [Out Patient Department], we all sit in the same place [and] necessarily having designated mental health facilities but right [from] where everybody goes access health services I get mental health services, that’s all.

**Interviewer**

Ok, thank you. So what opportunities or facilitators are there to support the development or the integration of mental health [services ] at the community level? Are there any opportunities that we can maximise or, yea, facilitating factors that can promote it

**Respondent**

Oh, I think that there’s a lot of opportunity. So, like I have been singing. In every facility, you would find at least, you will find a physician assistant. In every place you will find a physician assistant. The physician assistant per their training is able to make a diagnosis. Not just diagnosis [but] differential diagnosis so other conditions that can look like this, rule them out, manage appropriately, and then refer complex cases to higher authority. Apart from these people, you have medical or doctors. All these people are already providing health care. All we need to do is to do a little add on. so, we train them to be able to identify the common, common mental health disorders as they come into their consulting rooms and they manage them. And then if they are unable to then they refer to specialists. And the specialists are the … [psychiatrists?]. So, we can rely on these people to be able to do that. Then, apart from these people, I have said that we are already using our nurses. Well, people do not agree with me but the truth is that these nurses are a special workforce. Even though they have not be trained, you know, to be able to make a diagnosis and manage [mental health conditions], we are still using them to provide such services. Can’t we enhance their training to be able to do a better job? Because, when you go out there into your communities they’re a very passionate workforce. They work with so much enthusiasm and I’ve always said that if you meet a group of people that are already passionate about what they do, it is easier to teach them to do it better than people who are not interested at all. So, these people are already doing it, it just that the quality of the service they are giving is low. Can we improve on that by, you know, building their capacity to do it and giving them the resources to be able to do it. If we do that, then [mental health] service[s] will be available in our communities and we do not have to travel all the way to a specialised centre to seek help. The very complex ones then they refer. But of course, as they do this there has to be a system of monitoring what goes on otherwise, we will be creating mini-specialists everywhere and then, I know there is the danger of people saying that now we are in control and they will not even refer cases appropriately. So there has to be a system that monitors what goes on and then correct whatever is not going on right. This we can by, so apart from the training, or the capacity building that I am talking about, we can do the supportive supervision, come back and see how well they are doing. If we need to organise refresher courses you do that regularly, and then as they go/grow, then we will be building a workforce to be able to [be] adding on the numbers of people that are able to provide at least the basic health care that we can provide in our communities. and then the very complex ones, we can refer to the higher specialists.

**Moderator**

Ok, I want to find out how do you see the growing international focus and attention on mental health, advocacy and, growing civil society involvement in mental health advocacy impacting on Ghana’s mental health development at the community level?

**Respondent**

Erm, I am happy that at least there is growing interest in mental health, you know, globally [and] in Ghana as well. Erhm, but I think that it has to be put right. So, how are we going about it. Is it that somebody gets and says that oh, I have an interest in mental health and I want to do this and that and then we just allow people. I think it has to be streamlined. I expect, [and] I hope I am not being controversial here, but I expect the Mental Health Authority to up its game to be able to be to streamline all these things. Otherwise, what is going to happen is that, I think that, we will have a lot of people coming in, a lot of groups coming up saying we want to do this, we want to do that, which is all good but if we had a body or, for want of a better expression, a regulator that ensures that these activities are streamlined so that you don’t even have a duplication of the same activities of strategy. So that resources can be pooled together and then the work is streamlined such that they are doing it right and not just misusing resources. Because what could happen is a possibility of wastage of in terms of resources, especially financial. Because, I think that with the increase with the interest in mental health, you know, people are looking for opportunities. People are looking for opportunities everywhere. People will jump at it but what are they exactly doing? What impact will they be creating? So, there has to be a monitoring of it. There has to be streamlining of it. It’s not everybody who gets up and says I want to do mental health and you say ok fine we are happy, no, no, no. what exactly do you want to do? Whatever that you are interested in doing how, of what benefit would it be to the people that you are looking at? Could it be better if the same things being done by another agency so that you could look at this so that we do not have a duplication or replication, wastage and all that. So, I think that we should put all together and agree how it should be done so that we would minimise the wastage because, currently, I think there’s a lot of waste.

**Moderator**

*Mhm, hmm.* Ok. So we have talked about the opportunities [and facilitators], how about the challenges. What are the challenges that will still, that are likely to affect [the] effective integration or delivery of mental health services at the community level?

**Respondent**

*Hoh*, challenges. They are plenty [*laughing*]. It’s not easy. It’s not easy. The challenges are plenty but I think that I will just summarise. So, the first thing is, ok, ok. So, I remember once Dr Osei, he said [it] not once, he said it before that the head of a facility, a regional hospital, told him in his face [that] “mental health is not my priority”. “Mental health is not my priority”, so until we have [health facility] managers [who] that see mental health as a priority, this problem is not going to go away. And this is right from the community level to the national level. Even our policy makers and our legislators, are they interested in mental health? May be, I may digressing but I was telling xxxx, you know xxxx? xxxxx?

**Moderator**

Yah, xxxx. Of LEKMA? *mhmm*

**Respondent**

xxxxx. So, she is on the xxxxx thing.

**Moderator**

Ok

**Respondent**

Yes, I was telling xxxxxx that I think that we need to make our Legislators appreciate mental health, because, they are the ones that will formulate, you know, the laws, the policies and everything concerning mental health. We can make noise all day, all years, if they do not appreciate it, they will not, you know, buy into it and we will just be left hanging. Social … So how are you going to about it [and] I said I want to go into Parliament [of Ghana] and tell the people about mental health because the truth is that we are all living in denial. Mental health exists, it’s real but people don’t want to talk about mental health. Sometimes, when we have relatives who have these challenges or it could be us you don’t want people to even know that we have this challenge. And I was telling her when they have their people we go into their homes and attend to them when they see us privately and we are happy with that. When we meet outside, they talk as though or they behave as though they know nothing about mental health. Let’s go in there, let’s see us talking to them, let them appreciate that the problem is there. People are ignorant, so they don’t know and then they behave the way they are behaving. But, let’s get the right people to understand and appreciate it so that they can, you know, formulate the policy and when we formulate them to ensure that these ones are running and are being implemented and all that.

Apart from that, at the regional level, so for us in [the] Ghana Health Service we have our regional directorates, the regional directors of health services, are they interested in mental health? If you have a regional director [who] that is interested in mental health, it’s good because they will support that. Then you come to the district level, are the heads of the places interested? So, it has to come right from the top because at the top if the person at the top believes in it they are able to rally the people around and then they run with it. But, if you are down [and] the person at the top don’t see what you are doing, you will scream and scream and nothing will work. So, we need to get all the managers right from the top to understand. Then when you have brought all these people, who are the other relevant people to focus on? You can never succeed without our traditional leaders, our religious leaders, we cannot succeed because we are very communal people, very religious. We can’t succeed [without them] so we need to bring people on board. Then who are the others? We need the [Department of] Social Welfare. Are we even equipping them to be able to render the services that is needed? I think it is all about we not, you know, making mental health a priority because, it is true, it is expensive to run mental health [care services] and now we are saying mental health care is free. Who provides free services, free care or free health services in Ghana? The National Health Insurance [Scheme], why don’t we have mental health conditions as part of the conditions taken care of by the National Health Insurance. Isn’t it the same [National Health Insurance] Act that has brought us this far? Because the [National Health Insurance] Act says that mental health is free so the National Health Insurance doesn’t see why it should [pay for mental health services], but I think we should have a proper discussion because you cannot give services for free. Somebody must pay and who is paying?

*Erhm*, the Mental Health Authority has spoken about the LI, the funds and all that, me, I don’t believe in it. I don’t believe in that. It will not work. I think that if we are even looking at providing services at every place, integration, why do you want a separate fund in the first place? Go into the same pool that everybody is going to access help, the National Health [Insurance Scheme]. That money you can be sure that people will be able to get access and it should be [of] quality just as every other condition. Me, that’s [how] what I see it.

So those are challenges. The challenge [of] stigma, discrimination, even for us, as mental health professionals, people don’t understand why you want to do mental health or you want to pursue mental health of all the specialties. Even the facilities, people don’t want to get around them. So we until we are gotten beyond that which is going to be a very long process because it is deep-seated. Our understanding of mental is terrible as a people. But, I believe that we are gradually breaking ground. People now appreciate it better. We need commitment of the government as well. So, in terms of resource, human resource. We need to train a lot more people. So, human resource is one major challenge. We don’t have the requisite people to be able to provide services so we can look at that. Train more specialists, occupational therapists, mental health social workers, I mean, clinical psychologists. Currently, there are a lot of people that pursue mental psychology in school and they are unemployed. Meanwhile, there is a lot of work to be done. Why is so? You have nurses trained [and who are] unemployed. Why is it so? So, we need commitment from the government system. Political will should be there.

Then, I don’t know if I have mentioned the religious people because now when people are sick that is where they go to seek help so, we can’t succeed without these people. So, people have all sorts of views on that but I think that we cannot succeed without the people. I think that what we should try to do to bring them on board because no matter what do, we are very religious, superstitious people. So, even though I will come to the hospital, I will still go and see my Pastor or my who-ever-it-is. So, if we get these people to also appreciate mental health, what it is, how it can be managed and all that, when the people go there, at least they [will] understand that this is not a spiritual illness so they can, they will refer the people back. And like I always tell people I will never tell you to stop praying to whatever that you pray to but I will also encourage you to take your medications or whatever forms of treatment you are given, I will ask you to and encourage you to continue with it. I will not stop you [from] practising your faith or whatever. Because, even it is known that if you believe in something it serves as protection. So, we need to work hand in hand with these people.

The one challenge has been, so, for instance, if you take the challenge of suicide and then drug use disorders, yes, fortunately, the Mental Health Authority already started some work with the Judiciary. He xxxxxxxxxxxxxxxxxxxxxxx did some training for the members of the Judiciary. I think it is something [that] they will have to continue because these are challenges where we to see some form of disorders as criminal. And therefore, instead of getting proper help for the people, rather we push them behind bars, which shouldn’t be. So, that’s another major challenge.

**Moderator**

Mhmm.

**Respondent**

What else? We are talking about infrastructure. Yes, we don’t want big facilities but when we are building our hospitals, we should remember that there is a need for mental health care and therefore put in, at least, there should be a unit, a ward or something to take care of these people. We are saying that we cannot mix them with every other person. Yes, of course, sometimes if you somebody that is medically deranged, and then the way they behave and all that you cannot put them amongst people that are very, very sick but we want them managed in the same facilities. So we expect that when we are putting up structures or health facilities, the has been that we always forget that there is need to put up a mental health unit. So, that is another thing we need to look at. We should always remember to put in a mental health unit. And then when we are procuring our medications, that has been another major challenge. Because mental health is said to be free, people don’t want to stock medications because if it’s free will I sell the medications and how much profit am I going to make out of it? So, we don’t even want to stock. So, it still has to do with people at the top decision-making position being interested, thinking about mental health, being proactive and all that. So, these are the major, major challenges that I think of and possible solutions.

**Moderator**

Yes, I could hear challenges and you offering solutions [*langhs*]. Thank you so much. I think I couldn’t have had a better time with you than now. So, I have virtually ended my questions except to ask that if you have any other addition or anything else [that] you may want to add or if you also want to ask me a question relating to the study that you may not be clear with.

**Respondent**

Erhm, I do not have any questions. It is just my wish that we’re able to enhance mental health services. That is my wish.

**Moderator**

Indeed, that’s all our wish.

**Respondent**

Yes, we are able to provide quality [mental health] services throughout Ghana. I mean, I have been thinking, in terms of human resources, really what can we do? What can we do? Ahm, in our training institutions what can we do [with them]? Can we spend a little more time, you know, talking about mental health so that people have an interest. It is not just. For instance, in medical schools, until recently, people do just three weeks positioning/ residency in psychiatry and that’s it. But is that adequate? Because when you come out you meet them [people with mental illness]. That is how come people [medical officers] are not ready to see mental health cases. So, I think that we should be looking at the whole thing altogether, training in our institutions, erhm human resource allocation. So, right now if you go out into the regions for instance, in Ghana Health Service, a lot of people want to specialise in O&G, a lot of people [but] could there be a quota system [for that]. And then for people in a deprived specialised area like mental health could you give them a bit of freedom and said instead of insisting on may be a three year post housemanship whatsoever, can we reduce it to may be to one year, two year just to whip up the interest of people. And then there’s one thing that I am looking at. When we have brought them into the Ghana College [of Physicians and Surgeons] and they are done, can we say go back into the district, go back into the region and provide services for at least one year before you can come back and do your fellowship. Can we insist on that like they do in other specialised specialties. By so doing, gradually, at least, in very region we will have at least a psychiatrist that will oversee all these. And then as you go about building the capacity of the physician assistants and medical officers, at least you know that there are people on the ground that are seeing the common cases. If it becomes difficult, they have somebody close by [that] they can refer to. How can we do this? It’s a big challenge. I am talking to you because I know you can help me. You understand my issues.

**Moderator**

Absolutely.

**Respondent**

Yes, because I think that it’s a challenge. If you go into Komfo Anokye for instance. Komfo Anokye is just 11 bed capacity. Korle Bu Psychiatric Unit is just a 10-bed capacity, but you have a lot of specialists, what really are they doing? But look at the whole of Ghana, there’s no psychiatrist. I think it’s really, really a big issue. May be, you can all help me make that noise because, you can’t have six high psychiatrists sitting in a 10-bed unit doing what, when Ghana is as big as it is, all these people are down south Accra [and] Kumasi. It’s not right. It’s not right. So, [for] me I don’t have questions, when I have, I will ask. But I have a few clarifications [that] I have to read out for you of information I have to get for you. So, a document on the levels of care in Ghana if there are any, and then erh [human resource mix across every level]

**Moderator**

human resource mix across every level [of the health care system]

**Respondent**

Human resources. Actually, I am looking for my own report. I would have been able to get you some information.

**Moderator**

No problem.

Then planning for medical products for mental health, medicines, logistics

**Respondent**

I don’t think there is anything but I don’t want to say that

**Moderator**

Laughs

**Respondent**

laughs

**Moderator**

Ok, that’s fine.

**Respondent**

But I have to be careful when I am talking before they tell me to proceed on leave. Laughs.

**Moderator**

Laughs. I understand. Doc. thank you so much.

**Respondent**

Thank you too.

**Moderator**

And I hope that once in the north I can locate you in die course. We have the northern delicacy if possible. Laughs.

**Respondent**

Eih, I appreciate it. My people say we are going to have lunch at Akayet or so.

**Moderator**

Yes, they have good food there.

**Respondent**

They have good food there.

**Moderator**

They do. They do have good food.

The have a good pool there too. They have a good ambiance.

So, all the best. Enjoy Upper East. Bye for now.

**Respondent**

Bye

**Moderator**

Bye

| **Name of Interviewer/ Facilitator** | xxxxxxxxxxxx |
| --- | --- |
| **Name of note taker** | xxxxxxxxxxxx |
| **Type of respondent** | Mental Health Service users & caregivers |
| **Gender of respondent(s)** | Female |
| **Disability of respondent(s) (physical or psychosocial)** | Persons living with mental health conditions |
| **Age of respondent(s)** | Not asked |
| **Community Name** | xxxxxxxx, Bolgatanga |
| **Interview date** | 11^th^ August 2020 |
| **Start Time** | 10:05pm |
| **End time** | 11:15pm |
| **Recorder number** | VN870088.MP3 |
| **Name of file (follow convention below)** | Transcript_Focus Group Discussion_xxxxxxxxxxxxxxxxx.docx |

**Introduction**

This Focus Group Discussion (FGD) was held with a group of mental health service users from the xxxxxxxxx and xxxxxxxx communities within the xxxxxxxxx rea of the Upper East Region. The participants are members of a Self-Help Group (SHG) known as the xxxxx.

The participants were selected for this FGD because they were receiving regular treatment of their mental illness from the mental health units of the xxxxxxxxx regional hospital or the Sokabisi Community Health Planning Services (CHPS) Compound.

The interview was in Gurune (Frafra), the local language of the area and widely spoken language.

A digital voice recorder was used to record the discussion and which were complemented by handwritten notes. The details of the discussion and written up below.

**Moderator**

I would like to ask you to record the discussion and I want to ask your permission. Do you agree to that?

**Respondents/Participants**

Yes

**Moderator:**

As already mentioned to you, I am research into treatment provided to people with mental health care needs in the hospitals. I want to understand how treatment of mental illness at the community level is provided and how that can be improved to ensure every person who has a mental health need gets it. Whatever can be done to enable everyone, particularly persons living with mental health conditions, to have mental health care services closest to them and be supported to benefit and have better health. I am reaching out to a range of stakeholders and you who are caregivers of people living with mental health conditions for you to also share your perspectives. That is why I approached my junior brother [*the contact person*] to request you to have about five of you to have a discussion with you. I will ask questions and each of your is to say what she knows and experience. What we are doing is what is referred to as research. As we were conversing a while ago, we are not going to mention anyone’s name or say this person named said this. We will not say, [*for example*], Adongma said this or Atampoka said that. In the report that we will write, it will mention the groups/ categories of people who were interviewed or who we had discussions with and the perspectives they had on the range of issues that were discussed. No one’s name is going to be mentioned but it is important that that you agree that we can have the discussion. This is what I want to say. Do you agree that we can have the discussion?

**Respondents:**

Yes

**Moderator:**

I will ask each one of you to introduce herself. You will mention your name, you don’t have to get up for we are recording. If you know your age, you mention the number of years, and mention the name and sex/gender of the person with mental illness that you are caring for and the name of the mental illness of the person. We will record this first before we proceed with the questions, I want to ask you. Do you understand? Can we proceed?

**Respondents:**

Yes, we understand

**Moderator:**

Or my Frafra (Gurune) is not understandable?

**Respondents:**

It’s good and perfect.

**Moderator:**

I thank you.

Then I will proceed. Because I am recording I would like that you speak [out] audibly.

**Respondents:**

My name is xxxxxxxxxxxxxxxxxx. I am a member of the xxxxxxxxxxxxxxx. I take care of xxxxxxxxxx’s mental illness is such that he gets frightened/ shivers all of a sudden. It first started as though it was high fever. It progressed and now he gets frightened all of a sudden and he remains still. I have cared for him for quite a while and now I myself I have a mental health condition. I have had ear problems. This made me a patient and that is how I have become a leader of the group. I don’t know the name of the illness in the English Language. I am thirty-five years old. At the hospital, I was told the name of the illness is Epilepsy. xxxxxxxxxxxx has been living with the illness for 10 years now. He is my husband. We first started traditional treatment remedies and there were no results. We then proceeded to the hospital and have been attending the hospital many times up to now. When this group came about he and I became members of the it. Medical services were organised for us and with the medicines provided his condition has improved. Before this, things were challenging for us.

My name is xxxxxxxxxxxxx. I am taking care of my son. He is called xxxxxxxxxxxxxx. At the hospital I was told his condition is epilepsy. His condition began when he was three years old. We began treatment since then till now. We have been receiving treatment since then and that has helped to improve his condition. Our challenge now is the shortage of medicines. When you go to the clinic you are told that there are no medicines. It has been two years now since we got any medicines from the hospital. We are now buying the medicines and cost money. Anytime we get money to buy the medicines and he takes he gets well and moves about. I am forty-nine (49) years.

This group is up to ten (10) years now. xxxxxxxxxxxxis now twenty-one (21) years now.

**Moderator:**

Since when this did group start?

**Respondents:**

It’s up to ten (10) years now.

**Moderator:**

How old is xxxxxxxxxxxxx now?

**Respondents:**

He is twenty-one (21) years now.

**Moderator:**

Yes, my mother or should I say my mother-in-law

**Respondents -**

xxxxxxxxxxxxxxxxx**.** I myself, since I grew up, I have had a sever headache. It continued till I gave birth to my daughter. It was one night that he stretched himself and remained still and foaming from the mouth. It was initially said to be just a convulsive fit and we were advised to seek traditional treatment for that. She suffered the fit again and we decided to take him to the hospital where he was given an injection. She got an attack again for almost three months it was difficult to spot the iris of his eyes and he remained stiff. He was taken to the hospital and we continued visiting the hospital for several days. At a point traditional healer was brought to treat him for being possessed by spiritual forces. That helped improve his condition as her body relaxed from its stiff state. Her condition improved slightly. We decided to take him to the Nalerigu Baptist Medical Centre in Nalerigu, where he was treated. He was given some small tablets that we were to break in half and give him daily. Within three days his eyes returned to normal and he could now see well. There was some relief. My daughter was also easing on himself. Anytime she passed stool, it smeared all over him. We decided to take him a gain to the hospital for medical attention. We were given medication which we were giving to him. She has since gained some sense of awareness of himself [*consciousness/ insight/ discernment*] but she is unlike children of her age as his sense of reasoning is unlike her peers. When she mixes with other children, they despise him. The other children don’t want my child to play or interacts with them. This is mainly because of her lack of control of her bowels. We kept struggling with her like that till this group came about and we came to join. Through the group we were getting treatment and medicines but now we buy the medicines. When we get money we buy for her but when we are short of money she goes without the medication and when she relapses it is frightening. The signs of a relapse are that her eyes begin to turn red and her body muscles begin to become stiff. when we get the medicines and she takes, she come back to normal. Now with the help medicines she is able to live normally. She has since become pregnant and given birth.

Initially during our search for treatment, we were often told it was convulsion. As a result, [at the hospital], we were advised to make sure we always have ginger with us to insert into her anus in the event of an attack. We were always given some medicines. I was not told the name of the tablets they were giving to us, as far as I remember.

I think my daughter will be a little over 20 years.

I don’t remember the number of years of this group but I am one of the original members of the group.

**Moderator:**

My mother, you are also one of the caregivers of a persons with living with a mental health condition or epilepsy. Please, tell me about yourself.

**Respondent– xxxxxxxxxxxxxxxx**

My name is xxxxxxxxxxxxxx. I take care of my child called xxxxxxxxxxxxxx. I don’t know my age. My child at childhood was attending school normally. It was when she was about writing her [basic] school certificate exams and to complete basic school that she experienced the illness. She was at her desk about to start her exams when she suddenly fell off her chair. Before then he was diagnosed to have a hernia so when it happened we thought it was because of the hernia. Since her childhood, nothing of that sort happened to her. So we took her to have the surgery for the hernia. However, after the hernia the situation did not change. It was used to happen as though it was high fever. When it happens he starts to wander about. We were told the condition was as a result of spiritual possession for which he needed to be seek traditional treatment. A number of traditional treatments were sought but there was no change. We were then advised to take him to Kumasi for treatment which we did but there was no change. We proceeded to Accra but that also did not help. We went with him to the Nalerigu [Baptist Medical Centre] and it was there we informed that there is a place at the Bolgatanga Regional Hospital where they manage such conditions. We were told that the medicines they will give us [at the Baptist Medical Centre] will be the same as that which they will get in Bolga which will save them travel costs. We began going to the psychiatric unit of the Bolgatanga Hospital and Mr Tawah [a Psychiatric Nurse] was attending to us. It was Asibi, a member of the group, who visited our house and witnessed his relapse, who advised the child’s father to take him to the hospital in Bolga for attention and stop seeking treatment from the traditional healers. My son can get the attack and will have to stay indoors for days before coming out. Sometimes, he comes out of the room and just wanders about in the house or the neighbourhood till he is brought back. He can suffer the attack up to five times. We were told at the hospital that his condition is epilepsy.

Following the visit of Asibi, we were encouraged to join the group since there was an organisation interested in working with people with such conditions. I got registered and through your assistance, we got a medical team that assessed his conditions and recommended another type of medicine which has helped very much. The medicines were coming regularly and things changed for the better. Thanks to God, his condition has improved. However, over the last two years now the medicines have not been regular. We are now given prescriptions to buy the medicines. So, we buy the amount our money can afford. Despite this, his condition has improved greatly. It is not like the previous times when her colleagues despised her and did not want her to play with them. They will disperse when he is coming to join them making statements referring to his epilepsy.

Since joining the group however, the situation has changed. We get the medicines and we get recognition and our children get included. I thank you. I thank you very much.

**Moderator:**

Thank you. Now I can say we know one another. I am xxxxxxxxxx. I work for xxxxxxxxxxxxxx. However, I am a here as a student researching into mental health service provision at the community level for my school thesis report. Being the work that I do, I am interested in knowing more about hospital-based mental health treatment services that are provided in community-based health facilities. That is why I have a number of questions to ask so that you tell me what you know and your perspectives on the questions I am going to ask you. For this reason, I am going to ask again, where are the treatment services for the people with mental illness or epilepsy that you are caring for provided?

**Respondents:**

We go to the big hospital [Bolgatanga Regional Hospital].

**Interviewer:**

Do you all of you go to the big hospital [Bolgatanga Regional Hospital] or some of you go elsewhere?

**Respondents:**

We all go to the big hospital

**Moderator:**

I have discussed with some other people around here and they have informed me that there is a clinic in this area which they go. Is there anyone of you who goes there?

**Respondents:**

Yes, I go there.

The rest of us [3 members] go to the Regional *Hospital*.

For me, the issues is that, we all used to go the regional hospital. Then at one time we were all asked to be going to the clinic at Sokabisi but there was a time my child suffered the fit so much that we had to rush her to the clinic and there referred us to the regional hospital. When we got there they [*the mental health staff*] asked that why did I stop coming to them and now that I have a crises I am coming to them. I explained to them (*the mental health staff at the unit*] that they requested us to be going to the Sokabisi clinic which we complied with. It is those people at the Sokabisi clinic who have referred us to the mental health clinic at the regional hospital and that is why we are here. They then informed me that I have to return to them at the regional hospital so that when the child is in such crises, they can effectively manage it. But if I refuse their advice and continue to attend the clinic at Sokabisi and in a crises situation I come, they cannot do much. I agreed with them because if go to the mental health unit at the regional hospital you are more likely to get medicines. Unless there is a shortage, if not you will always be served when you go there but that is not the case with the Sokabisi clinic. That is why I reverted to the mental health unit at the Bolgatanga Regional Hospital

**Moderator:**

Those of you who are attending the clinic at Sokabisi, you were called by name and specifically instructed to go [referred] there or you were informed that there is a clinic at Sokabisi and you could go there for services? How did that come about?

**Respondents:**

We those living in Sokabisi, there is one of the [mental health] nurses there who attends to our mental health needs for which we do not have to travel far for the services. At one time we went to Plaza[[1]](#footnote-1) for medical attention. It was there that we were told to our medical/clinical files have been handed over to the [mental health] nurse at Sokabisi and from hence we should be attending there for reviews. He [*the mental health nurse*] has our medical/ clinical files. Our files were handed to him to take care of us.

**Moderator:**

So the staff of the regional mental health unit selected your files and handed them over to the [mental health] nurse at Sokabisi clinic and informed you to be going there for treatment?

**Respondents:**

Yes. You see, the issue is that we were always crowded any time we went to the regional hospital for treatment services. The crowding distracted the concentration of the nurses attending to us. Occasionally scuffles broke out among the caregivers and/or service users. The decision by the staff to ask some of us to come to the Sokabisi for treatment/ review of treatment was to avert the crowding and bring about some order. It was for that reason that the nurses at the regional hospital decided to ask some of us to start going to Sokabisi for attention. We were told that anyone of us who is near a clinic [*a community health facility*] with mental health services provided should go there. Once they split us up we could go for treatment with less crowding and quarrels as clearly we were all ill people and fighting among ourselves should be the last thing to happen to us.

**Moderator:**

Is there anything to add.

**Respondents:**

I have something to add. It relates to the regional hospital that we are discussion now. The people from Sokabisi wanted us [*from Yikene*] to ask for our medical files to be removed from the regional hospital to the Sokabisi clinic but the staff at the mental health unit of the Bolgatanga Regional Hospital refused to do so. There was a time when one big person [*psychiatrist*] was coming to consult with patients as part of their training. We were asked to go a bring our medical/clinical folders from the regional hospital but we were denied the folders. It was the nurses themselves who brought the folders for the outreach and after we were attended to, the nurses returned the medical folders to the hospital to keep with them. What I want to say is that we have always been treated at the regional hospital and no where else.

**Moderator:**

Ok. So, when you take your patients [*persons with mental health conditions or epilepsy*] for treatment, be it at the clinic of the regional hospital, what kind of treatment are you provided in these health facilities? What is the treatment made up of?

**Respondents:**

On arrival, they will first take the temperature of the patient. After that you go take the folder [*from the Out-Patient Department*]. When you get your folder you go collect your medicines.

**Moderator:**

Does anything else happen. When you arrive they just take your temperature and then they take your name to go on to pick your medical folder/clinical file and that is it? What else happens?

**Respondents:**

They will also ask you how you [*the patient is feeling*] are feeling.

**Moderator:**

When you follow your child for mental attention for its mental ill-health/ epilepsy condition what they do?

**Respondents:**

After taking the body temperature and getting your folder, they ask you as how you are feeling being on the medication provided. They also ask to know if the patient takes the medicines as prescribed.

**Moderator:**

How about the rest of you? Is it the same as those who have just spoken, or it is different. What treatment is the person with the mental health condition/ health?

**Respondents:**

My daughter goes there monthly to collect here medications. When the month ends and her medicines are finished, even if there are no medicines in the hospital, they will first draw her blood sample for a laboratory test. She will also be given medicines. When she was pregnant, she was told the medicine was too strong. For that reason, they will temporarily suspend giving her the medicines. She was asked to stop taking the medicines. The nurses advised that as soon as she gave birth, we went back and the nurses advised that she should allow the baby to grow up a bit more and when the child is no longer breastfeeding then she can commence with the medications. The reason why they asked that she not take the medicines while breastfeeding was that the medicines could lead to the breast milk not been produced in good quantities for the baby to suck and that could affect the baby.

I was now going alone to take my medication.

**Moderator:**

Ok, you started going alone.

When you go for treatment and they ask about how you are fairing with the medicine prescribed for you and then give you more medicines, is there any advise that they add?

**Respondents:**

Yes, they always impress upon us that when we get home we should ensure the medicines are taken as prescribed. We should not allow the patient to consume alcohol. That consumption of alcohol compromises the effect the medicine(s) will have had patient. We are to ensure they eat well.

We know that mental health services are to support the poor and sick ones, but they are not easily available. We still walk miles to access treatment services at the regional hospital

As for my husband in each of his visits to the mental health clinic his blood sample is taken for laboratory test. This is a check up on him.

**Moderator:**

Is this for every month that your husband visits the clinic?

**Respondents:**

Yes, because he now has high blood pressure too.

**Moderator:**

Ok, when they do that what else happens?

**Respondents:**

After the blood test, he is given his medicines. If there are shortages in the hospital a prescription is written out for us to go and buy. He is asked to buy the medicines to last a month after which he is to come for a review.

**Moderator:**

Ok, so the advice is that you should ensure the patients take the medicines as prescribed and they should not take alcohol?

**Respondents:**

Yes. They also give advice to we the caregivers. They impress upon us to understand that as caregivers we need to have a lot of understanding for our patients and accommodate their excesses. Their illness makes them say certain things that could be hurting but is it important that we deal with them with tact. As caregivers, we should avoid making references to their illness or make derogatory or insulting statements. It can make them take actions that will not augur well for you the caregiver and their very selves. For that matter we need to take a lot or care in looking after our relatives who living with the illness.

**Moderator:**

How about the health workers, do they visit you in your homes to see to the health condition of the persons living with the mental health conditions/ epilepsy?

**Respondents:**

Yes, they come visit us. Our nurse at xxxxxxx comes around. We do not have a unit in our clinic, we have to go to the mental health unit at the regional hospital. If it takes time and he expects that the medicines should have been finished for which you should have come for a refill; if you miss the monthly review that you are expected to go to the health facility to have with the [mental health] nurse and for your medicines and another month comes around you will see him in your house.

**Moderator:**

So he comes when he realises that you missed your monthly attendance for review and collection of your medicines?

**Respondents:**

Yes, he will seek to know whether the medicines have been finished or not and why you have not come for the monthly review. One time we went review and he prescribed medicines which we were using but which did not finish so we decided not to go for the review. After we had missed a month and went, he asked why we didn’t come for review and I told him that the medicines we had were not finished, that is why we did not come. He told me that whether the medicines are finished or not each month it is important that we come for review. He told us that it is not proper to use an old prescription to buy a refill of the medicines. He told us that most pharmacies don’t allow that. He said, we were to come for the review and if there are medicines, he can give us a top up for the month and if needed a fresh prescription form to go and buy the medicines.

**Moderator:**

Who else has something to add. Do the nurses visit and what is the frequency of the visits? Who visits you? Do visit weekly, monthly or how? As she has said, if you miss the scheduled monthly review they follow up to find out why

**Respondents:**

Yes, they visit. For us who go to the regional hospital, it is the same. If you miss your scheduled monthly review a select team of two or three nurses visit you at home to find out why you did not come for review. In the visit, they want to know if the medicines for your relative is still there or not. The team has visited my in this manner a number of times.

**Moderator:**

Ok, when they come, what conversation to they have with you? What subjects do you discuss? Is it only when you miss a month review appointment that the follow-up to find out why or they visit to chat with you and know more about the progress of the treatment of your relative

**Respondents:**

First they tell you they have come to find out why you missed your monthly review, next, the want to know if you still have your medicines for the patient. They will ask that you bring out the medicines for them to inspect. Next, they will want to know how you follow the prescriptions. So if they say the medicine should be taken only in the night, they will ask how you are taking it – they will check if you are taking it in the night or you take in the morning and in the night? They will reiterate the advice that the patient should not be taking alcohol while on the medication. They impress on us to make sure the patient is fed well as taking the medicines must be supported with the patient eating well. They also tell the rest of the members of the household not to behave in ways that annoy the patient and the caregiver that everybody needs to be supportive in the treatment of the patient.

We are not to discuss the services delivered in the hospital. Apart from the nurse asking you how are feeling when you go for your medicines, we are not consulted. The NGOs like BasicNeeds meet us. They encourage us to organise our groups so that we can support one another and help people to go for treatment.

**Moderator:**

I thank you. Has anyone of you got something to add?

**Respondents:**

Yes, I have. When we were living here in Yikene, we got visits from the nurses as described already but we relocated to Sumbrungo and we have never been visited since we moved there. It is because they don’t know where we have moved to and the location is also far. However, each time we go to the clinic there advise us always to ensure we are helping our relative taking the treatment to take their medicines as prescribed. We ensure they are fed well as the medicines are strong and the people under treatment need to eat well.

**Moderator:**

I thank you. Let me end here and move on to another area. You are here in your homes. Do you find that all who need hospital-based treatment for mental illness or epilepsy get them in the communities?

**Respondents:**

Yes, Now {laughing} the traditional herbalists are no longer there so everyone has made the hospital (formal health care services] our home. Now everyone attends church. As everyone now is a Christian, who is going to the soothsayer to find out what sacrifices to make and which traditional healers to go to or invite to come and administer treatment? Out home is now the hospital. When your feel unwell, that is where you go to.

**Moderator:**

So, everyone who requires treatment for his/ her mental illness or epilepsy gets it provided to him/her by formal health care services?

**Respondents:**

Truly, there are people who still don’t understand mental illness or epilepsy. I visited Kulgo [ a nearby village] and I saw young people with mental health care needs and it was pitiful. When I mentioned to their parents they need for them to be sent for treatment, the parents complained that they had no money. Despite not having money, it is important that they are kept clean and well looked after but this was not the case. I encouraged them to try and send their children to seek medical attention.

We were like these people I saw at Kolgo but thanks to the contact we have had with you people we know better and are taking care and included. The surrounding areas need to be reached. I also went to Zorko and witnessed another situation where a woman beat up her daughter who was agitated and restless. I had to appeal to her to stop maltreating her for it is not her making. For a girl at that age will not intentionally do the things she is doing. But for resource constraints, it is important that we go round end educate people about the need to take their relative with mental health care needs to the hospital for treatment. There is still a lot of ignorance out there and people need to be educated. It is important to let people know that help is at the hospitals. You see people with such children in great difficulty, making them maltreat their biological children and that is unfortunate. Every parent had children in the hope that their children will one day take care of them. It’s unfortunate they turn out that way and a lot of patience is required to care for them.

**Moderator:**

What I have heard from you is that there is ignorance with not much information about the availability of treatment for mental illness or epilepsy while other cite lack of money to take their relatives to be provided treatment in the hospital.

I now want to ask do you know the people in the health facilities who are taking care of you? The health care workers who are treating you, you know them to be who?

**Respondents:**

BasicNeeds and

**Moderator:**

BasicNeeds does not do the medical treatment that you are provided. The people treating you at the regional hospital and Sokabisi are not BasicNeeds staff. Do you know those people treating you who they are?

**Respondents:**

They are nurses and doctors.

**Moderators:**

So those looking after you are nurses [and doctors]. Are there others in the hospitals and clinics who treat mental illness or epilepsy that you know of?

**Respondents:**

No. we don’t know.

**Moderators:**

But who said nurses and doctors? You know doctors look after you. Which doctors are looking after you.

**Respondents:**

They are the doctors who treat mental illness.

**Moderators:**

Do you know the names of the doctors?

**Respondents:**

One is called xxxxxxxxx.

**Moderator:**

No, I don’t mean their real names but the doctor profession that they learnt and qualified in.

**Respondents:**

No, we don’t. we just know them and nurses and doctors.

The doctors are those who treat mental illness or those with disability.

**Moderator:**

Have you heard the word psychiatrist?

**Respondents:**

No.

**Moderator:**

And have you heard of one of the doctors known as Clinical Psychologist?

**Respondents:**

No, I have not.

Yes, We have heard of such a name.

**Moderator:**

Have you ever had a clinical psychologist attend to you?

**Respondents:**

When you get to the place and [incomplete sentence]

One doctor came some time back. We went and were attended to by him. They mentioned his name but I have forgotten.

**Moderator:**

So, you know nurses and doctors treatment you. However, from your perspective do you think the health workers treating should be more than just nurses and doctors or it is just alright?

**Respondents:**

Truly, it is for nurses and doctors to treat these kinds of conditions. They are not illnesses that traditional healers should treat.

**Moderator:**

Looking among you, I see some of you have had formal education. When they say specialist, I guess you understand. Are there specialist in the health facilities that you go for treatment? They are the ones that I have mentioned as psychiatrists, clinical psychologists, are they there in there in the health facilities to provide you treatment?

**Respondents:**

It was a few years ago that some doctors came from Accra and we went there and they attended to us.

**Moderator:**

How many years now since that visit? But in the health facilities do you have them?

**Respondents:**

{with some hesitation}. If they are there we would not know. Unless the mention them we wouldn’t know.

We don’t know.

**Moderator:**

Do you know the calibre and category of nurses and doctors who should have been there to provide you with the treatment for mental illness or epilepsy? Whether they are nurses, psychiatrists, and clinical psychologists you are not able to distinguish that?

**Respondents:**

Yes. we don’t know.

**Moderator:**
How are the nurses and doctors trained and qualify and come to provide the treatment?

**Respondents:**

No idea

**Moderators:**

Ok, that’s fine. I now want to turn to the treatment you receive in the health facilities, do you pay for the services provided?

**Respondents:**

No, we do not pay. They treat us free of charge.

**Moderator:**

I heard one of you say they write out a prescription for you to go and buy.

**Respondents:**

Yes, that is when the medicine is not available. They write a prescription for us to go and buy.

**Moderator:**

So, when you attend the clinic and you are called to meet with the nurse and you are asked questions about the persons taking the treatment is faring, the taking of your blood for laboratory tests and you temperature is checked is all free of charge?

**Respondents:**

Except the laboratory test of the blood everything else is free.

At first, the nurses used to ask us to give them one [Ghana] Cedis for them to provide us the medicines. We did not agree, pointing out to the nurses that the treatment is supposed to be free. We held a meeting and invited our big people [pointing to Bernard Azuure]. They in turn invited the nurses here for a meeting. Understanding was reached and they dropped the demand of Ghana One Cedis for the medicine and gave us medicines anytime they had stocks. When the medicines ran out to prescribe for us to go and buy.

**Moderator:**

In that case any payments you make relate to payment for medicines.

**Respondents:**

Yes

**Moderator:**

How about the laboratory test of the blood? When the draw your husband’s Atanga’s blood for testing do you pay?

**Respondents:**
No. I don’t pay

**Moderator:**

Is the health insurance [National Health Insurance Scheme (NHIS)] working? Does your health insurance work for you with regards getting treatment for mental illness or epilepsy?

**Respondents:**

We used to use it for treatment at the mental health unit and they will provide us the medicines. However, it got to a point that they said the insurance [National Health Insurance] cannot work again. That is what led to us buying the medicines. They informed us that the government does not reimburse the drug-stores [pharmacies] for the medicines they supply. We did not complain. It got to another time that we were told that the health insurance will be renewed free of charge.

**Moderator:**

For how many years now since the free renewal of health insurance premiums began?

**Respondents:**

This lasted for about a year now; [for] almost two years that we were having our health insurance cards renewed free of charge. We could go a collect a letter and then gather the cards of our members in bulk and give them to the leader of our group to take to the offices of the health insurance scheme for renewal. We used the cards to collect the medicines free of charge. Then at a point they said it was is possible again to use the insurance for free. At this point too, the medicines are not available. Now even if you have a valid insurance card you will still have to go and buy the medicines [for the patient]. There are no medicines that you will be provided at the health facility.

**Moderator:**

So, the insurance not really working now as it as previous times or it is just not working at all.

**Respondents:**

Now the health insurance card does not work at all. When you reach there and present it they only use it to retrieve your folder. They will then check your temperature. Should there be need for a laboratory test of your blood sample that may be covered but that’s it. For the medicines you will be given a prescription to go an buy.

**Moderator:**It means the health insurance card works in some aspects and not in others.

You have said that when you go, you are medically checked – they take your temperature, take samples of your blood for testing - and you are educated/ counselled on how to handle your relatives who are receiving treatment for their mental illness or epilepsy. Who do you think pays for all these services that you mention that your are provided at the hospital or clinic?

**Respondents:**

The government pays for that.

**Moderator:**

The government pays? And who else?

**Respondents:**

It is the government.

**Moderator:**

Do you know of any others who pay for those services you are provided at the hospitals or the clinic?

**Respondents:**

We cannot know. It is the government we can think of.

**Moderator**

Explain to me any mental healthcare services provided by private clinics or places that you use.

**Respondents:**

For us here, it is only the psychiatric unit that is at the regional hospital. We have many private hospitals and maternity homes, but none offer mental healthcare services. It is only at the government hospitals that mental healthcare services are provided.

There are no private services. Even those we here provide services, they are not real. They want to make money.

**Moderator:**

If the government pays, do you know of organisations that support/ complement? Do you know of other organisations that help to improve the health of your relatives living with mental illness or epilepsy.

**Respondents:**

It is BasicNeeds [Ghana] that is the only other organisations that supports.

**Moderator:**

Which others do you know of?

**Respondents:**

Even if there are others who support us, it will be via BasicNeeds [Ghana]. We do not know of any other agency. There are no organisations that know us, except through you [BasicNeeds-Ghana].

**Moderator:**

I thank you. Considering the money government is providing towards your treatment, are those monies [funds] enough or government could have done better? More could have been provided than is prevailing.

From your perspective, knowing what is taking place at the hospital when you go for treatment, with some of you saying that you have been going from treatment form those facilities for close to ten years now or more, what will you say about the kind of treatment services provided do you think the treatment is adequate or more could have been done?

**Respondents:**

We think more could have been done. Government could have done better. We are virtually patients ourselves. We do not have the energy work to earn the income that can enable us buy the medicines. When you get there [the hospital] there are no medicines available. It would have proper that you get the medicines regularly as the nature of our conditions is that we should not default with taking our medications. There are times that the medicines will finish but there is no money to buy the medicines for your relative living with the mental health condition. When you break with taking the medicines it worsens your condition. What should therefore happen is or the government to make sure there are medicines all the time so that we don’t default. The government should also ensure that the national health insurance cards works and we are able to have our cards renewed free of charge. This is what will help our relative who are living with mental illness or epilepsy.

**Moderator:**

Ok. Has anyone got anything else to add?

**Respondents:**

I have something to add. What she has just said is true. The medicines are everything for us. we wished it [the government] did more that but we understand that there are difficulties in the world. We are not the people with this kind of conditions. There others elsewhere and we cannot complain that the government should attend to only us alone. It is challenging on this earth but we think that renewal of the health insurance card should be the one thing the government should continue to do for us so that we can be getting the medicines. For this illness, if you are skip in taking the medicines even a day your situation changes for the worse. There is no stable work that you can do to regularly earn to buy the medicines. It we can say is to continue to pray to God for God to open good doors for your those who are leading us to have good health and work to be able to in turn help us. this can help us prolong our lives.

**Moderator:**

I thank you. Now if there is nothing else to add on funding of mental health services at the community level what the government [of Ghana] should have been doing and what other organisations are supporting mental health care services at the community level, I want to ask if you have heard of the mental health law and do you have any information about it?

The government has put in place a law specifically to address mental health issues and of persons living with mental illness. Are you aware of the Mental Health Law which aims to look at persons living with mental illness or epilepsy. Do you have information on it, are you aware about the law?

**Respondents:**

I have not heard.

I have not heard of it [mental health law] too.

Yes, we heard of it. We were informed about it in a meeting that we were called to attend at Taboore-Tindongo [a section of Bolgatanga]. At that meeting we were made to understand that government has a responsibility to look after groups like ours in Bolga[tanga] here. That every person living with mental illness wherever located should be assisted by the government. The law requires of government as such. That is how they shared the information with us at the meeting that we attended.

There was another time that we were informed that the government has authorised that homes should be visited to find persons living mental illness or epilepsy in homes who have not been brought out to receive medical attention or to participate activities. The government has put in place a law like us. We too who are virtually ill and taking care of our relatives we should take it upon ourselves to educate people in our communities who have persons with the conditions that our relatives have that we are caring for to come out and to utilise the services available to help improve the health and wellbeing of the people living with mental illness.

**Moderator:**

Fine, so. what else about the Law do you know?

**Respondents:**

We were also made to understand that as caregivers we are to ensure our relatives living with the condition are kept safe and that they don’t wander about and engage in unwarranted acts/activities. However, should anyone abuse them or make derogatory remarks/ references towards them. People should attempt to remove people living with mental illness from their place of work or jobs they are engaged in. We should report such people to the appropriate authorities to deal with the culprits.

**Moderator:**

How about you the caregivers and the persons living with the mental health conditions, was there indication in the you are to speak out [advocate] for better treatment for improved health? Were you informed that you can let the health workers know how you feel about the treatment services they provide you or suggest to them ways by which they and the government should support you to improve treatment and health?

**Respondents:**

Yes. They made us know that the law provides for them. There was a time our members leaders went to the [FM] radio station to discuss about the right of every person living with mental illness to be free to engage in whatever endeavour of interest to him/ her. Persons living with mental illness must not be hidden away in home. We did speak about this over the radio that the Law of the government a person with mental illness or whoever to be free to live a normal life and do what ever pleases him just as everyone else.

**Moderator:**

I am now going to ask you another question. As you go to the health facility accompanying your relatives with mental illness for treatment, have the health workers ever invited you to have a discussion with them for you to tell how else they could improve their services that they provide to you?

**Respondents:**

No we have not been invited for such discussions.

**Moderator:**

But have you people yourselves come together to discuss and take decisions of suggestions on ways treatment provided to your relatives with mental illness can be improved that you put before the health workers work with?

**Respondents:**

No, we have not.

There was one time that there was a discussion. One woman narrated that she was pregnant and went to the hospital to seek attention and informed the health workers that she had a disability as she was sight impairment. The health workers there replied to her that if she knew she has sight challenges why did she get pregnant. We had a discussion and it was decided that the nurses should be informed that being vulnerable, when we come to the health facilities they need to recognise that we are vulnerable people and give us the much needed attention this is better than the persons living with mental illness and seeking treatment at the hospitals mixing with the other patients queuing up for services. In the queues you struggle with each other as you shift towards the service point and you to hear derogatory statements made towards them which do not augur well. There is need for us to be given a separate space so that when we come, we get served without much delay. This allows

**Moderator:**

The nurses did not call you for you to give them this feedback but how did you convey your concerns to them. Dis it reach them?

**Respondents:**

No. It was our collective concern that we took to them [the health workers/nurses]. We wrote a letter with these concerns and sent to the health workers at the hospital.

**Moderator:**

Who signed the letter that you wrote?

**Respondents**

xxxxxxxxxxxxxxxxxxx

**Moderator:**

It means the health workers have never invited you to a meeting to discuss ways to improve their services they provide. Ok, let’s proceed. We will soon end our discussion.

Considering the discussion we have had so far. Has anyone come in such manner to hold discussions with you, informing you that he is carrying out a research to explore how provision of mental health care treatment services can be improved? Have you been invited to have this kind of conversation?

**Respondents:**

No

**Moderator:**

But do you know that there people, students from schools/ universities who go around researching into various subjects of interest to them as part of writing on how treatment services and care of persons living with mental illnesses can be better support to improve their health and wellbeing for their academic qualifications?

**Respondents:**

Yes. We have information about that. There have been up to two people who came to our house and asked to speak to an grown up/elderly person who can tell them about the history of the community – how things began – for his test [examination] but for a group like for us to have a conversation as we are having no, not yet. After the conversation with the elderly person we didn’t ask what the whole conversation was about.

**Moderator:**

How about when you go to the hospital and they take your name, where you come from and other details, do you know what they use such information for?

**Respondents:**

No, we don’t know.

**Moderator:**

Ok, at the start of this conversation I explained that this is an effort to understand how formal health facility treatment of mental illnesses or epilepsy can be widely available within our communities. In your view how can treatment services be widely available to everyone who has need of such services?

**Respondents:**

I think as we have just done, working as a group. we those who are lucky to benefit from treatment services need to raise our voices for those who are yet to benefit to be aware and come forward to get treated.

**Moderator:**

So you mean you will reach those who living in the communities with mental illness but are yet to get treatment to come forward and access the treatment?

**Respondent:**

Yes.

**Moderator:**

Alright, it means you those who have benefited you will tell others to come forward and also get treated but I mean from the point of the hospital how can we ensure that treatment is available every one and so that anyone with a mental health care need gets it within proximity of their community or home(s)?

**Respondent:**

It would have been good for clinics with mental health services to be spread across our communities. If there is a clinic in our community that provides mental health care treatment services it will make things easier for us. there are people in our community when you mention to them that the mental illness their relative is living with can be treated they retort by saying they don’t have the means to get the person there, they don’t have money to take them to the hospital for treatment. So, if the clinics are closer to us it will help.

**Moderator:**

Has anyone else got something to add.

**Respondents:**

I have something to add. I am hoping that those of you who are enlightened can continue to support us. Some of the people who support us have motorcycle and other don’t. some have bicycles. Those with motorcycles face challenges with getting fuel to move around to support us. We are appealing that the government also pays some attention to help such workers and us. you those who are enlightened and have motorcycles and bicycles, however far the location with the motorcycles and bicycles you are able to reach and with God’s help you will be able to educate/advise them. As our brother has just as our brother has indicated that it becomes possible for doctors to be everywhere to help give us treatment to improve our health and lives. This will help bring about development in the land (community/country/ world). This is what I have to add.

**Moderator:**

Thanks to you. In your view/ thoughts what do you think will prevent services being widespread from being realised? What will make treatment services not to be widely available as you have desired?

**Respondents:**

When our illness and vulnerabilities are reduced and we can become productive and useful to ourselves. We are more or less living with ill-health and are vulnerable. You have no work or means of earning income. It makes one sometimes think that being dead is better than being alive in this state. But if each day you find yourself occupied doing some productive activity that can earn you income/ a living you will find meaning to live and be happy. You pray that God should help you to also be counted among people.

**Moderator:**

I am done now. If there is anyone of you who wants to ask me a question or thinks we should have discussed something that I have not brought up in this our conversation or you have something in mind that you wish us to discuss you can bring it up now.

**Respondents:**

I have something to add. In these political campaigns, when the politicians come around to campaign people living with mental illness go there to listen and they accept them but when it comes to the point of voting, people with mental illness are not allowed. They are prevented and maltreated and claim that they are not fit to vote.

What my sister has said is true. My son went to register for the Ghana Card and the people were hooting at him and did not allow him to register. For two consecutive days he got up early at 1 am in the morning and went and queued for the card but was prevented on each of the occasions. He was able to complete the form alright and was now waiting to go in a take his photograph. Knowing how they can behave when they are tired and frustrated, he spoke angrily and that led to him being prevented from taking his photo to get the Ghana Card. They asked him to go a get a guarantor and someone agreed to serve as one but they still refused to allow him complete the process. It led to some pandemonium and the people there began saying that a mad man has come to disrupt the registration exercise. Police were called in however, luckily before they arrived he was whisked away on a motorcycle. You can imagine what could have befallen him if the police arrived. My son felt hard done. His form was torn away.

**Moderator:**

Is there any other matter. I have ended my questions and I thank you. I will go and write out my report. Should I reach an area that I will want some more information, I will contact my brother Ben and he will reach out to you for the information. I thank you very much.

Reflections:
This was a very insightful discussion. The participants were frank and forthcoming. Being they seemed well organised and sought to project the advocacy activities of the group. getting to know that I work for BasicNeeds-Ghana did not seem to affect the discussions in anyway.

| **Name of Interviewer/Facilitator** | xxxxxxxxx |
| --- | --- |
| **Name of note taker** | xxxxxxxxx |
| **Type of participant** | Mental Health Service user |
| **Gender of participant** | Female |
| **Disability of respondent(s) (physical or psychosocial)** | Person living with mental illness |
| **Age of respondent(s)** | 27years |
| **Community Name** | …, Bolgatanga |
| **Interview date** | Not indicated |
| **Duration of interview** | 27.20min |
| **Recorder number** | VN870089.MP3 |
| **Name of file (follow convention below)** | Transcript_individual interview_Mental Health service user.female. Bolgatanga.docx |

**Introduction**

This individual interview was held with a person with mental health condition from a community (name of community not indicated) within the Bolgtatanga Municipal Area of the Upper East Region. The participant is a member of a Self-Help Group (SHG) known as the xxxxx.

The participant was selected for this interview because she was receiving regular treatment of the mental illness from the mental health units of the Bolgatanga regional hospital.

The interview was in Gurune (Frafra), the local language of the area and widely spoken language. A digital voice recorder was used to record the discussion and which were complemented by handwritten notes. The details of the discussion and written up below

**Interviewer**

I have now pressed the button because you said you have agreed. Just write their names in a fresh page. Errrh! So I have now pressed the button for the recording. Errrh… our discussion is a student conversation, just like when they say research. We are conducting a research to investigate persons with mental health conditions who are receiving treatment at the hospital to know how we can expand the treatment to cover everybody. We did a research to investigate to know. So now we want to have a conversation with everybody, focusing on people receiving treatment from the hospital, those taking care of patients with mental illness and epilepsy and all those providing treatment to them. We want to have a conversation with all these people. So you are the one I was asked to contact so we can have this discussion. I thank you for making the effort for us to meet.

I am going around having discussions and investigating to know how it is. So you can also introduce yourself, tell me who you are, how old you are, are you someone who is a patient or caregiver, if you know your illness you can tell me (the English name), your age and name. Do you understand what I am saying?

**Respondent**

My name is xxxxxxxxxxxxx. I have mental illness.

**Interviewer**

Do you know your age?

**Respondent**

Yes I do. I am twenty seven years of age.

**Interviewer**

Do you have an idea of how long you have been going through this illness?

**Respondent**

I have been going through this for three years now.

**Interviewer**

Before the illness started, where were you and what were you doing?

**Respondent**

Before the illness I was in school.

**Interviewer**

Which school?

**Respondent**

I was attending Zebilla [*Senior High School*] when the illness started.

**Interviewer**

Is that a secondary school?

**Respondent**

Yes

**Interviewer**

And did you take it to the hospital?

**Respondent**

I went to the hospital. When the illness started initially, I was not taken to the hospital. But I was taken for treatment at traditional healers. When they got me there they could not manage my illness. And we went somewhere else and they could not. And they went to pastors, but it was not possible. But when BasicNeeds came, I used to run around and wonder about not knowing where I was. But when the BasicNeeds came, they instructed me to go to the hospital for drugs and I was taking the drugs till I got better.

**Interviewer**

So when you went to the hospital, what did they say was the name of the illness?

**Respondent**

That…mental… psychiatric… errh! patients!

**Interviewer**

Did they just say Psychiatric patient?

**Respondent**

No… like insanity/ madness… hahaaa… l don’t even know… like mental…

**Interviewer**

As I indicated, I just asked about your name but when we are to write the report, we will not mention your name a say you said this… we will just say we had a discussion with people and these were some of their views.

As of now, where do you go for treatment?

**Respondent**

I go to the Regional Hospital for my treatment.

**Interviewer**

Do you know any other place people suffering from mental illness go for their treatment?

**Respondent**

No, I don't know any other place except the Regional Hospital.

**Interviewer**

Are you are aware there are clinics in the communities where people are treated?

**Respondent**

Small hospitals? Yes I sometimes see small hospitals making announcements…

**Interviewer**

No, I mean hospitals… small hospitals, what they call clinic. Are you aware of any located in the community?

**Respondent**

Apart from the regional hospital, I don’t know of any

**Interviewer**

Have you heard about the Sɔkabihi clinic?

**Respondent**

Oooh yes! I have heard about it.

**Interviewer**

Have you ever been there?

**Respondent**

I have never been there.

**Interviewer**

Whenever you visit the hospital, what treatment do they give you?

**Respondent**

My head… they usually give drugs for my headache.

**Interviewer**

So when you get to the hospital, what exactly do they do?

**Respondent**

They often take my folder to check and ask me to go for drugs. Sometimes they write in it and ask me to purchase it from the drug store. They usually tell me to continue taking the medication. Sometimes the doctor finds out whether there is improvement with the medications I am taking. Whenever, I go there they are usually aware I am coming for my medication. Sometimes Anytime I go to the hospital, I usually go to see the doctor if the drugs I'm taking is useful in treating my illness and after that, the doctor then prescribes some drugs for me to buy at the pharmacy. Sometimes the doctor do ask me of how I feel after taking my drugs.

And what do you usually tell him?

I usually tell him that whenever I take the drugs, I always feel much better.

**Interviewer**

Do they usually take your temperature?

**Respondent**

Yes, they do.

**Moderator**

Do they usually take your blood for laboratory test?

**Respondent**

No, they don’t take my blood sample to the laboratory. Sometimes they give me injections. They give me injections.

**Interviewer**

Who are the health personnels that are in charge of your treatment? Not their names

**Respondent**

They are nurses

**Interviewer**

Is there a doctor in addition?

**Respondent**

Doctor… A doctor came sometime ago.

**Interviewer**

Was he a visiting doctor? Or he is there already?

**Respondent**

He was a visiting doctor. I went there but could not see him because the people were many.

**Interviewer**

Did he work the next day or not?

**Respondent**

It seems he did not week the next day

**Interviewer**

You said nurses take care of you. Apart from nurses are there other professionals that should be taking care of you?

**Respondent**

Yes, the people from BasicNeeds.

**Interviewer**

But the people from Basic Needs are not doctors

**Respondent**

Hahaaaa… but don’t they take care of us?

**Interviewer**

Apart from the nurses, the psychiatrist, the clinical psychologist, social workers and the occupational therapist are also supposed to be there. Do you have all these people in the clinic?

**Respondent**

No, I have never heard of them but I think we need their support also to help take care of our illness.

**Interviewer**

I want to know, the treatment they are giving to people with mental health and epilepsy. Is the treatment extended to cover other patients suffering from the mental illness in the community?

**Respondent**

No, I don't think so because others are not aware that they can go to the hospital for the treatment.

**Interviewer**

Don't you think it is due to inadequate health personnels that's why others are not aware that there is treatment in the hospital for those suffering from mental illness and epilepsy?

**Respondent**

Yes, I think so and something should be worked on immediately. Sometime ago, some government officials came to the hospital to investigate and know how we are responding to treatment with the drugs we were taking. And we told them how good the drugs has been and how it has made us to feel better.

**Interviewer**

Have you ever heard of the mental health law?

**Respondent**

I have never heard of any mental health law.

**Interviewer**

OK. Have you heard of the data collection before ?

**Respondent**

No, I haven't.

**Interviewer**

Whenever you go to the hospital and they do ask you your name, age and how you are responding to treatment with the drugs you have been given all consist data collection. The questions they usually ask you in the hospital, do you know what they usually use it for?

**Respondent**

I don't know what they usually use it for.

**Interviewer**

Why don't you know what it's used for?

**Respondent**

I don't know because if the health personnel doesn't make it known to us then we would not also know what they would use it for.

**Interviewer**

Do you pay some bills in the hospital for taking your temperature?

**Respondent**

We don't pay any bills for taking our temperature.

**Interviewer**

Why don't you pay any bills for taking your temperature?

**Respondent**

I think they are assisting us.

**Interviewer**

As they are not taking any money for your bills, who do you think is paying them?

**Respondent**

The government is the one paying them since they are working in a public hospital.

**Interviewer**

Apart from the government, do you know any other people that are motivating the health personnel?

**Respondent**

I don't know any.

**Interviewer**

Is the health insurance beneficial to you?

**Respondent**

Yes, it's beneficial to us because that is what we use to take our drugs without making any payment.

**Interviewer**

Apart from the government and the National Health Insurance Scheme, is there any other place the nurses get motivation from which can also be used in assisting you?

**Respondent**

No, I don't know of any other place.

**Interviewer**

Has there been anyone here before to have a conversation with you and also to know your intention concerning research?

**Respondent**

Yes, someone has ever been here.

**Interviewer**

What can we do to extend treatment to other patients suffering from mental health or epilepsy in this Region?

**Respondent**

In extending the treatment to patients suffering from the same illness, I think it lies in our hands. If we the patients who are receiving treatment gets in contact with other patients who are not receiving treatment, then we can direct them to the hospital so that they can also receive treatment.

**Interviewer**

What can we also do to help in treating this illness and also to extend the treatment to other patients?

**Respondent**

I think you people can also join our group and assist financially or in any other way you can.

**Interviewer**

On this note, I want to give you the opportunity to tell me your intentions concerning this conversation in case there is something bothering you and you would like to voice it out.

**Respondent**

Actually, I have nothing much to say except that the stigmatization and discrimination is what we don't like. Even when we were doing the Ghana card, the people there didn't value us but were rather looking down upon us and also anytime we have a meeting, we are always taken for granted which we don't deserve.

**Interviewer**

So as their leader what did you do? Did you write a letter to the National Identification concerning what you were going through?

**Respondent**

No, we didn't do anything as such.

**Interviewer**

OK. Thank you very much for your time.

| **Name of Interviewer/Facilitator** | xxxxxx |
| --- | --- |
| **Name of note taker** | xxxxxxx |
| **Type of participant** | Caretaker (father) |
| **Gender of participant** | Male |
| **Disability of respondent (s) (physical or psycho-social)** | N/A |
| **Age pf participant** | 70 |
| **Community name** | Not indicated |
| **Interview date** | 11^th^ August,2020 |
| **Duration of interview** | 33 minutes 1 second |
| **Recorder number** | VN870091.MP3 |
| **Name of file (follow convention below)** | Transcript individual interview. |

**Introduction**

This individual interview was held with a caretaker of a mentally challenged patient in the Bolgatanga municipal in the upper east region of Ghana. The participant was selected because he is a caretaker of mentally challenged patient. The interview was in Frafra and a digital voice recorder was used to record the discussion which was translated into English and complemented with a hand written notes. The details of the discussion is written below.

**Interviewer**

Am aware you’ve sat for quite too long possibly feeling some discomfort on your buttocks.

**Respondent**

Its well. Nothing comes easy. If you don’t do it that way you can’t handle mankind. Its about having patience.

**Interviewer**

Have you agreed for us to have this conversation recorded?

**Respondent**

Yes please

**Moderator**

What we are to discuss about is what we term research. How will we ensure that the drugs that are being given to mentally challenged persons or epileptic persons are easily accessible with minimal stress? We wish to plead to caretakers of the sick, the sick themselves, people providing health care services and others assisting in the provision of health to carry out this assignment for documentation purposes which may help government to implement measures to assist in this battle and not for any other purpose aside the mentioned above. For this reason we came to plead to in seeking your consent to carry out this assignment, do you agree for us to carry this out?

**Respondent**

Why wouldn’t, I agree, I do agree

**Moderator**

Thank you for this honor.

I will like to ask of your name, your father’s name, your age, if you are looking after the sick or you are yourself by any means challenged, the treatment of it according to healthcare provider you can let me know after which I will then ask subsequent questions. What is your name?

**Respondent**

My name is xxxx, my father is also called xxxxxx

**Moderator**

Do you know your age?

**Respondent**

For the age, was my document to be around I could tell but now, I think I will be around 70 or 71

**Moderator**

So you are around 70?

**Respondent**

Yes

**Moderator**

Thank you very much. Who is the person that's sick or yourself?

**Participant**

A child of mine that's sick.

**Moderator**

So you are the caretaker of your child?

**Respondent**

Yes please

**Moderator**

What’s the child's name?

**Participant**

She’s called xxxxx but locally named as xxxxxx.

**Moderator**

Meaning xxxxxxxxxx?

**Participant**

Yes please

**Moderator**

Do you know the name of the condition according to medical diagnosis?

**Participant**

Medically they said it’s a mental illness

**Moderator**

Did they mention to you the specific mental condition?

**Respondent**

Personally I know its madness since am an adult and matured enough.

**Moderator**

At the hospital, did they make mention of the specific mental condition?

**Respondent**

The reason is, I've not being visiting the facility with her personally due to financial challenges, its the mother that used to go with her. As she used to go with the mum, they said it was mental illness. It got to a time she wasn’t visiting the health center any longer. When it happens that way you have to then be persuading her to but when you you compel her she wouldn't just go at all.

**Moderator**

So what was she diagnosed of? Like they will tell others that they have malaria, its fever or its diarrhoea, they didn’t asked to be told as such?

**Respondent**

They only said its mental illness but we are aware its madness. Just because I wasn’t of myself by then if not I would've gone there with her myself.

**Moderator**

OK, thank you for that. The other question is, where do they go for the drugs at the hospital for the mental illness?

**Respondent**

When you get to the hospital, they are normally behind the maternity block side, towards Zaare. That’s where they are located.

**Moderator**

Where you directed as where they take the drugs, that's the regional hospital. But are you aware there are other health facilities you can visit to get such drugs?

**Respondent**

The truth is, I wouldn't lie, that's where they've been sending her and we've not inquired to know if there are other alternatives.

**Moderator**

Does Yikene has a medical center just as Sokabisi do?

**Respondent**

Yes they do have

**Moderator**

So are you aware that people with mental illness are being treated there?

**Respondent**

Frankly speaking I’ve not been able to ask to know such. I know they have a clinic but I’ve nit asked such to know.

**Moderator**

Okay. So when they go to the hospital, concerning their treatment, how do they care for them?

**Respondent**

The doctor himself caters for them.

**Moderator**

How does the doctor caters for them? In what ways?

**Respondent**

Sometimes they look at their speech, appearance and behavior.

**Moderator**

Have you ever seen them take blood samples for laboratory investigations?

**Respondent**

I've never seen them take blood sample for laboratory investigation. I wouldn't lie.

**Moderator**

But do they normally check for BP?

**Respondent**

Yes

**Moderator**

Do they check his weight?

**Respondent**

Yes they do

**Moderator**

After these, what next do they do?

**Respondent**

After these they proceed to where they care for them

**Moderator**

What then happens there?

**Respondent**

They will also assess to know how the sickness looks like

**Moderator**

After this what again?

**Respondent**

They then proceed to give injection

**Moderator**

What next after the injection?

**Respondent**

They then give the drugs and educate them on how the drugs are to be taken then ask to return when the medication finishes.

**Moderator**

Do they give advice to you the parent yourself?

**Respondent**

Yes, they do

**Moderator**

What advice do they give?

**Respondent**

They ask us to come with them as early as possible when we detect any sign of the condition. They also ask us not to disturb them or speak abusive words to them but we should be persuading in an open and welcoming manner.

**Moderator**

Thank you for that. But what advice do they normally give to the sick person?

**Respondent**

They normally ask her to always behave and comply with medication. She shouldn't also to aggressive or engage in activities that are too energy demanding.

How they should communicate with people and how to manage their temper if not it aggravates the condition.

**Moderator**

Okay, thank you for that. Now looking at the treatment they give for people with mental illness and epileptic patients, how does it go?

**Respondent**

Truthfully speaking people with mental illness are increasing almost on daily basis.

**Moderator**

As they are increasing that way, do you think their treatment are also increasing that way?

**Respondent**

The treatment too they are treating

**Moderator**

Are the treatments everywhere?

**Respondent**

The treatments are at the hospitals but I don’t think they will be at the clinics.

**Interviewer**

Why do you think the treatments will noise be at the clinics?

**Respondent**

I've not visited the clinic since the condition started. I've only visited the hospital.

**Interviewer**

That’s why I asked of the clinics. I asked whether you know that the drugs for mental illnesses and epilepsy are at the clinics of which you responded that you don’t know. Then you also said the mental illness are increasing and I asked as to whether you think how they are increasing, the treatments are also increasing that way. Are there hospital treatments available that anymore can access care with ease?

**Respondent**

We have scientific treatment and local treatment

**Interviewer**

Am not talking of local treatment. Am asking of the scientific treatment. It is available and accessible that anyone can reach out to for treatment?

**Respondent**

It’s the hospital that has the drugs because it can’t be everywhere to be used for treatment. With the clinics, when they detect that it’s a mental illness they only refer you to the hospital. But because I’ve nit sent her to the clinics I can’t tell but I know even if you send her there, they are definitely going to tell you to send her to the hospital because they wouldn't have drugs to be given.

**Interviewer**

Okay, thank you for that. So how are we going to ensure that the hospital treatment is available for all so when the need be they can easily get access to for treatment?

**Respondent**

I will say, if it was possible, they could get a clinic precisely form the treatment of mental illness and made known by all.

**Interviewer**

So the government should do so right?

**Respondent**

Its the responsibility of the government to do so. Looking at us, do we have anything to do to help? So if the government doesn't help, how are we going to do so. We can’t also use our own authority and just begin something like that because we don’t have.

**Interviewer**

Is it the money you don’t have or the resources?

**Respondent**

My son, when the situation becomes worse now and I don’t get money to reach her to the hospital then it has to become worse. That’s what am talking about.

**Interviewer**

Okay, I also want to ask, those at the hospital, who are they?

**Respondent**

Who are they as in? Who are they as in males or females?

**Interviewer**

Who are they in the work they do?

**Respondent**

They are professionals.

**Interviewer**

Are they doctors or nurses?

**Respondent**

They have their nurses and doctors there.

**Interviewer**

So those attending to people like Faustina, are they nurses or doctors?

**Respondent**

They are doctors

**Interviewer**

Do you know their names? Not their names as xxxxxxxxxx but something like this person is a psychologist, psychiatrist, social worker and this person is a quality control, do you know such as they who treat mental illness?

**Respondent**

I won’t lie, normally when I get there, my attention is always to get my daughter attended to and nothing else. Anyone coming I wouldn't be able to tell whether this or that.

**Interviewer**

So you’ve never heard of a psychiatrist?

**Respondent**

Psychiatrist?

**Interviewer**

Yes, you’ve never heard?

**Respondent**

They've been calling but only do I hear. I don’t know the meaning.

**Interviewer**

You’ve not also heard of a clinical psychologist?

**Respondent**

I have heard that one too but don’t know the meaning.

**Interviewer**

What of occupational therapist? Have you heard of that before?

**Respondent**

Yes

**Interviewer**

And what does such a person do?

**Participant**

They also assist

**Interviewer**

So, looking at it knowing that there are nurses and doctors who are there, are there supposed to be any other group of people included or they alone are okay?

**Participant**

Adding to those working there?

**Interviewer**

Yes

**Respondent**

There’s the need that others are added to work there to facilitate the work and reduce he work load on them. So its good to add.

**Interviewer**

You even said you don’t know their differences. Though I wanted to ask if there's the need that the psychiatrist and psychologist are there but you said you don’t know them so I will proceed to the next question. Do you pay for the services?

**Respondent**

Truly we don’t pay

**Interviewer**

You don’t pay?

**Respondent**

Yes we don’t pay, we've never being asked to pay. It’s only the folders that we are asked to pay for.

**Interviewer**

So now that you don’t pay, whose money do you think they use in payment?

**Respondent**

I know it’s the government

**Moderator**

Only government or others are also involved?

**Respondent**

Some assembly members are also involved.

**Moderator**

Assembly members as in?

**Respondent**

You know I can’t investigate the hospital to know but its possible other agencies are supporting.

**Moderator**

What am now asking is, when you take government out, to the best of your knowledge who are those supporting?

**Respondent**

I can’t really tell

**Moderator**

Okay. Thank you much. But the insurance you are having, is it beneficial? Does it help you get drugs as in mental health treatment?

**Respondent**

Yes

**Moderator**

Your own or it’s for xxxxxxxxxxx?

**Respondent**

We use all

**Moderator**

And what happens after that?

**Respondent**

Due to that we get the drugs for free.

**Moderator**

You don’t pay anything again?

**Respondent**

No! When they take that one that's all.

**Moderator**

So with the health insurance, when you get there, what can it pay for you?

**Respondent**

They will take it and check its validity. If it’s expired, they can’t care for you.

**Moderator**

The drugs you said they sometimes writes for you to buy, does it incur cost for you or you feel government will refund it at the appropriate time?

**Respondent**

How will government refund, if you buy that's for the health of your relative.

**Moderator**

I will also want to ask, did you attend school?

**Respondent**

I never schooled, I went one day and I was beaten by my father that why did I go to school leaving the cattle, because of that I never went back.

**Moderator**

Have you as a relative of a mentally challenged person ever being invited to the health center to discuss how to handle you relatives in order to improve their health?

**Respondent**

Yes they have

**Moderator**

Like how many times?

**Respondent**

Severally

**Moderator**

Have you attended before?

**Respondent**

No. My wife wasn’t around and myself I couldn’t make it there.

**Moderator**

Have you ever sent a suggestion to the healthcare workers as this is what we think that if you it will help us all? Have you done such before?

**Respondent**

We haven't done such before

**Moderator**

I am left with small for us to end our discussion

**Respondent**

Okay

**Moderator**

Have you heard of the mental health law by government for mental and epileptic patients?

**Respondent**

Law?

**Moderator**

Yes, concerning mentally challenged and epileptic patients

**Respondent**

Am not aware

**Moderator**

Was to ask whether they have a right or not but now that you don’t know of the law its okay. So what will we do to ensure that the health care provision is widespread so people can get it at any point in time and accessible?

**Respondent**

When you have a father you run to him in time of need. Let your request be made known. Something like getting clinics for mental health treatment so we don’t always have to ran to the hospital all the time when situation become bad.

**Moderator**

You said run to your father, which father are you talking of?

**Respondent**

Government of course

**Moderator**

Government? So he does what?

**Participant**

So he can help us.

**Moderator**

Help you in what way?

**Respondent**

Help as in providing clinics for mental health treatment. This will help even at midnight and times when you can’t afford transport to reach the hospital.

**Moderator**

Aside government, who can also help?

**Respondent**

Aside government, the next in line of action is the traditional ruler. When we reach out to him and he’s able also reach out to those who matters and can help.

**Moderator**

You can reach out to government to help but what can prevent this from happening? Is it conflict or not being serious?

**Respondent**

You plead to him to help with the traditional ruler as a mediator in the absence of conflict?

**Moderator**

Please, tell me more about how people like you who are caring for persons living with mental health conditions participate in the mental healthcare services provided or planning them to ensure that your ideas and key aspects of the services that need to be address are attended to?

**Respondent**

We are not involved much. Even though we have experiences, we don’t think we are expert enough or qualified to contribute ideas. The people in-charge don’t ask us and we don’t request to be involved. We ensure we go for the services on the days that you were scheduled to from your last visit.

**Moderator**

From what you have described, surely you could make a contribution to how services can be improved or better provided, how can you do that?

**Respondent**

Mmmm, I think they should invite us to meetings to discuss how the services can better serve us. One of the NGOs, BasicNeeds, they make an effort and encourage us to form groups to express our concerns. We meet and tell them or our problems of caring for our people with the illness. This is not so with the nurses or the hospitals.

**Moderator**

Please, kindly explain to me how you can be more involved in determining the quality services that can better help with the recovery of the people with mental health conditions.

**Respondent**

Errh, hmm. Education to help us know more about mental health and to help in promoting mental wellbeing is not taking place in our community. Perhaps it is yet to happen. We will welcome it if it does. For me, just as many of us parents with these kind children, we are carrying a heavy burden. You are so occupied but if we can be of help to others to improve care or even nurse hope, I will.

**Moderator**

Do you also have anything to ask or clarify about our discussion?

**Respondent**

You said?

**Moderator**

Maybe something you want to ask for clarification or contribution concerning what we've discussed.

**Respondent**

What I have to add is for you to proceed for this to reach the top for them to help us.

**Moderator**

I appreciate your time and effort for making this discussion a fruitful one. You’ve been here since morning, I really appreciate it. But we have a document for you to sign to show we've actually had such a conversation with you though we didn’t give you water.

**Respondent**

I am rather supposed to give you water and not you giving me water again

| **Name of Interviewer/ Facilitator** | xxxxxxxxxxxx |
| --- | --- |
| **Name of note taker** | xxxxxxxxxxxx |
| **Type of participant** | Health Service Manager |
| **Gender of participant** | Male |
| **Disability of respondent(s) (physical or psychosocial)** | N/A |
| **Age of participant** | Not indicated |
| **Community Name** | Tamale |
| **Interview date** | 19^th^ August 2020 |
| **Duration of interview** | 1hr14min |
| **Recorder number** | VN870096.MP3 |
| **Name of file (follow convention below)** | Transcript individual interview. xxxxxxxxxxxxx.docx |

**Introduction**

This individual interview was held with the xxxxxxxxxxxxxxx at the Ghana Health Service in the xxxxxxxxxxxxx of Ghana. The participant was selected for this interview because he is a health professional and a public health expert. The interview was in English. A digital voice recorder was used to record the discussion and which were complemented by handwritten notes. The details of the discussion and written up below

**Interviewer:**

Good afternoon sir again. My name is xxxxx

**Respondent:**

Good afternoon.

**Interviewer:**

I am a student the school of public health, university of Ghana doing a PhD and I have decided to focus on thesis research on integration of mental health at the community level. I am trying to understand how the role and influence of the WHO health system framework has on development of mental health at the community level in Ghana's health system. So in short, that is the focus of my research. And I am doing a mixed study. I'm undertaking a survey with a questionnaire, which is being done by a data collection agent and I'm also undertaking the qualitative data collection in the form of key informant interviews the focus group discussions and I'm leading that myself. As I have mentioned in my application to you, you are a key stakeholder in the health system in Ghana. Particularly in the locations where I want to collect the data and errrh I very much think my study would have been incomplete if I don't get to speak with you as a leader of health services in the xxxxxxx region. So thank you so much for giving me the opportunity.

**Respondent:**

You're welcome

**Interviewer:**

I am most grateful

**Respondent:**

You're welcome

**Interviewer:**

I realized you are one of the early pioneers of the school of public health.

**Respondent:**

laughs...

**Interviewer:**

So you're the trail blazers?

**Respondent:**

Yeah, 1999

**Interviewer:**

So emmmm… this is to the gauge your perspectives. I don't know if you had opportunity to do the online survey but I believe that would give you an idea about some of the questions. I want to explore but they would range from service organization, human resources, financing medical products, leadership and governance issues, yeah, to information and all that. So thank you once again. I hope if we have not finished completing the consent form by the end of this, we would do it.

**Respondent:**

Yes, we would do that by the end of this.

**Interviewer:**

So I would like you to introduce yourself, your position title and what that means, and how long you've been in this job.

**Participant:**

xxxxxxxxxxxx is my name, the regional director of health services for the northern region. Emmmm… I got to the xxxxxxxxxxxxxx here in xxxxx as a xxxxxxxxxx but I have been in other regions as deputy director for public health in Central region and Greater Accra. But I can say in the health service as a whole I have been there for more than 25 years.

**Interviewer:**

Great! Thank you.

**Respondent:**

You're welcome

**Interviewer:**

What services are available for treating mental health conditions in community base health facilities in the Ghana health service?

**Respondent:**

Laughs... Let me start first by saying that, errrrh… currently I would say, though we have an authority which is responsible for mental health you can see that the structure or it is still in a kind of way that I would say its only actually visible at the very top of its governance structure but immediately we would want to come down from the national level to the regional level that distinction appears to sort of errrh disappear because the services and everything is done by the Ghana health service. We have coordinators, regional coordinators who are under the authority but when it comes to the operations at this level they are under the Ghana health service because they report here directly to the deputy director clinical care. Then if you go below you would realize that we also have in most of our hospitals we have designated other units or wards depending on the size for psychiatric care and when you go into the community, then you would be talking about the community psychiatric nurses. They take care of people directly in the community so they would be doing the minor things and refer things that go beyond them to the hospitals. So you realize that, yes we have people who have been trained but their community health nurses and these community health nurses are Ghana health service staff. That is why I said authority structure only appears to be very visible at the top but we work hand in hand from regional right down to community level. We don't show any distinction.

**Interviewer:**

So typically if you get into the Ghana health service facility, I'm interested in the district or subdistrict health facility. What services are you likely you get?, what mental health services is one likely to get?

**Respondent:**

Since we are talking about the community level, let me take it from there. The CHPS and the health center are within the community. In those places, that's where the community health nurses actually operate so you realize that most of what they would be doing there could be health education on mental issues and the mental issues here includes; epilepsy, drug abuse, and those other issues. So they give education on those ones. And some of the districts within the area where they have maybe a psychiatric nurse, they may be able to give some of the drugs that solves some of the problems of mental disorders. So at that level basically, it is mainly education and management of minor ailments where the hospital also has a psychiatric nurse. Let me also state that, we have situation where a whole district may not even have a psychiatric nurse, so that gap is there. So once you realize that the district or community nurses may have to refer to the neighbouring facility which may be in another district or even in another region depending on the location of the district. So at that level, those are the kind of services you are likely to get.

**Interviewer:**

So these services you have described and the levels you earlier on talked about, are they documented anywhere to show that we documents in terms of level of service provision across the health system.

**Respondent:**

That's why I was saying that you see in the previous system it is just what we are kind of holding because if you look at mental health as a service it has been quite disadvantaged. Even as a country I remember we can boast of three key psychiatric hospitals and then we had some regional hospitals which has an attachment of a psychiatric unit. Those are the kind of things we had. So you realize that, I remember those days we posted those who have gone for psychiatric training so we had district psychiatric nurses. So they are in charge of the district. They come and go all other the place. Now maybe we have gotten more who are the community ones that's why now we can have a situation where people are in the community level otherwise those days it was the district psychiatric officer who moved from one community to another for the whole district but now the numbers are more so the sit and have people working under him in various zones or communities that he has created. So that has been the picture. So won't say that we can see a very distinct difference showing that this level this is a category of staff because now we realized that the service is seeing mental health as belonging to another service delivering body. That's where the challenge is because for instance the coordinator we have here is a coordinator for mental health for the region and his payments are under the authority so we cannot do much unless the authority. I have not seen what structure they have come out with apart from the one we are trying to collaborate to see that this is the distinct structure. But whatever we have now is what we are doing. It may not be the authority’s thing. I have not seen a document to that effect there maybe one but I have not seen it. That is why I told you, now we have a regional coordinator and then we have district coordinators that he will operate with and then within the districts they have community psychiatric nurses who are directly in the community. This is how it is done and then you know as the regional hospital which I know, they are supposed to have a psychiatric unit and the regional hospitals some of them too are well structured so they also have a unit but the district hospitals, most of them doesn't have a ward. And even like the hospital I have talked about, if you realize we don't have any hospital in the north here. If you have a hospital in this area that's Upper West, Upper East, and Northern region, it's likely to be private or faith based either an individual is trying to sort out the problem otherwise we don't have those things.

**Interviewer:**

Yeah, so I want to talk about the fact that, what likely services you get at the community level with the community education about mental health. Some of them also they give medicines. Do you think they do diagnosis at that level?

**Respondent:**

You know some of the health centers have psychiatric nurses attached there. They can do minor diagnosis because when I am looking at mental health, it looks like if somebody comes with chronic insomnia, chronic epilepsy, yes they can manage those ones. But not when we are talking about things like schizophrenia or Bipolar disorder. Those ones no. At the level No.

**Interviewer:**

Okay

**Respondent:**

Epilepsy and those other things can be managed at that level

**Interviewer:**

Is there a situation where conditions like epilepsy are seen at the general health facilities?

**Respondent:**

Yeah, you see, usually it's not like we have specialized structures so most of the cases we get we first run to the nearest facility which is the general facility and especially this area where we don't even have psychiatric facilities and people don't perceive epilepsy as a mental condition so they bring it to the general hospital which they will treat because they may not from the first know it's a mental problem because other conditions can make you sick and so they cone to the general facility where the doctor would then say that, from the history of the examination, I think this person is suffering from epilepsy and then may need more special attention of a psychiatrist otherwise they would go to the general hospital and they would be treated if they think maybe it's a fit due to fever in the case of children.

**Interviewer:**

Thank you! So generally how accessible are mental health services to the population at the community level? This accessibility is more around geographical access.

**Respondent:**

Okay, let me say that the access situation is a twofold affair one; there are places where maybe there might even be a structure for treating mental health condition but people will not go because of the perception of what they think is the cause of mental health but strictly in the health sense I don't think we have enough access especially in our part of the country. We don't have enough access because if we have any structures here apart from maybe the wing from regional hospital there is no district that has a facility which is purely mental. There is nothing like that so the access is grossly inadequate. Apart from that that is why I'm saying the other challenge too is that the people don't perceive mental health issues as hospital issues; they look at it more spiritual so you would even realize that from that angle they don't even know that they don't have access. So they don't even know that they don't have access to what they actually deserve that is why they end up in spiritual homes and camps where they are abused and violated and all kind of things. So that is the way I look at it. The access is not there and the people also don't seem to realize that they don't actually have the kind of access they need because they don't even see the problem as a hospital problem. So that means that our education must be tightened or intensified to make sure the appropriate what actually is causing mental health and that it can't be solely attributed to spiritual issues.

**Interviewer:**

How about been able to utilize the service affordability? How to generally get the services provided, do we see them as affordable to the average individuals?

**Respondent:**

You know originally, psychiatric case management or treatment is for free but I don't know with time either the complaints that have always been that's the reason why they come into the news any way, is that either there are no drugs for them and patient's relatives have to buy drugs or they are not available. So that has been the challenge on and off. So yes, the medicines they need don't seem to be available as expected and like I said it was free but we also got to a point where we realized that since the thing is not available and somebody gets it from somewhere we are forced to charge the people which most of those people couldn't afford because most of them these are conditions which has been prolonged, they are no longer engaged in any meaningful job so they don't have means, they rely on relatives and relatives after a while would not be able to continue supporting them that way so any amount you may think is very small may not be affordable to them.

**Interviewer:**

Thank you so much sir,

**Respondent:**

You're welcome.

**Interviewer:**

I want to move on to human resources

**Respondent:**

Okay, feel free.

**Interviewer:**

So how and in what ways are human resources developed and deployed for mental health services at the community level?

**Respondent:**

The human resources that we have mainly now is the nurses because we realized that even among the doctors we are very few doctors moving to psychiatric at least in our part of the world so they train community health nurses as psychiatric personnel and general nurses also go for training as psychiatric nurses and so when they come those are the ones that we deploy to the various levels so it's mainly nurses that are involved in the care of psychiatrists at this lower level. It is the hospitals I think that you would get few doctors who are psychiatrists. That is why they are actually overwhelmed.

**Interviewer:**

Okay, so are there a mixed of nurses, we have talked about general nurses some are community health nurses that have go to specialize, are there some other caliber of nurses that are operating in the mental health space?

**Respondent:**

Apart from the community, and general nurses, the public health nurses, no it's rare. It's mainly the community health nurses and then the other nurses.

**Interviewer:**

Okay. So I think you have probably answered the question but let me ask for clarity. Do we have a human resource manual or a professional mixed document anywhere that describes at every level what mixed of staff is available. To say alright, if you come to the community mental health facility, we have A, B, C, D type of staff and then if you go to the next level, these are the kind of staff you should have. Do you know what kind of mixed staff should be in each level?

**Respondent**

I have not seen a document to that effect but I can say that from what is the practical that is happening, the district and lower level are mainly community health nurses and then the district level we may get a general nurse some will say enrolled nurse or some of them are the RGN [Registered General Nurse] been a district person. Then at the regional level these are the coordinators they bring in. They are some of the senior nursing officers who are degree nurses. They are general nurses but with degrees or if you want to go to the nursing where I came from, somebody would have been qualified to be principal or DDNS and those things but they are still general nurses. That's the way I look at it. Then in some of the facilities, the hospitals which we don't have any in our part we have the doctors being the psychiatrist. Some places you could see medical assistant attending to them but they don't take themselves as solely for mental health. Yeah, so that's what we have. So mainly we realized that most of the psychiatrists that's the doctors are in the psychiatric hospitals and then the regional hospitals where they have units for them. But you realize that in the hospitals where there are solely mental hospitals you would get various mixed of people there because they go beyond just directional measures. Because we need certain technicians who use gadgets, they have lab people because they treat them like any other patient so those other mixed of people are there who the lab people use the lab for and then you would see some general nurses not necessarily trained in the psychiatric and then we have psychotherapist in some of them but you realize that these are not common. Some of them do maybe part time there. They are not there solely for that but I believe partly it's because the mental health as a service has not developed in the country. That's why some of those things are not there. I know they are busy doing it, you know it was 2018 that an act was passed through so they are still in the developing process.

**Interviewer**

Thank you Sir! So I mean from where you're sitting, in the idea of situation what would have been the mixed of staff at each level? If you had all that want, all the caliber of staff, what would be the staffing mixed of each level.

**Respondent**

You see, I don't think the staffing mixed should be any different from other patients because you see, they may come with supposed mental reasons but they have just other things like any other patient comes in with. Because the truth is that, some of them if you see them, somebody needs to tell you that we are dealing with him as a mental patient. Do they still need all other services. So we shouldn't sort of, I don't know how to say it, discriminate and say they should have this, they shouldn't have this. They should have all so that when they go there it becomes a stocked shop. It's not like you go there oh, this one is mental this thing so go to the facility and when you finish, these people don't work here. So now from mental you go there for BP to be checked and then so others. No! No! I would prefer that if we say we are giving service to them. This is a hospital setting, all staffing that are needed for any other condition should be there in addition to specialized people in the psychiatric or mental health. That's the way I want to look at it. So lab X-ray and what do we call it?

**Interviewer:**

Which category of them are for key management?

**Respondent:**

All category of nurses. If we have the luxury, the category of doctors based on specialization, they would be in the facility so that when they come, if you finish seeing the person for whatever mental health and maybe in the process of examination you realize that this person has a heart problem as well or has some other surgical problems. They should be able to just move to the next room and take care of them.

**Interviewer:**

So that removes the stigma?

**Respondent:**

So in the ideal situation that's the way it should be but you know ideal things don't happen but we can work towards it. We may not get the complete but that picture will be there which can even make other people walk into that facility because they would be given the treatment. They may only make special arrangement for the aggressive ones so that their ward and things would be different than people usually trained to manage them handle them.

**Interviewer:**

Thank you

**Respondent:**

You're welcome

**Interviewer:**

So I was going to ask if you have professionals like clinical psychologist and occupational therapist ehm What level do you think they would most effective?

**Respondent:**

Oh, you see for me those category of people should be right down from the district level because the challenges we have as a country is where it train just a few of this crop we are talking about because most of our special this things are packed at the top there and then people go through a trouble from district to district referral, referral, referral until they get there and you realize that they go and accumulate there then it becomes a problem at the top so people start queuing and that's where issues of ehm abuse of their rights comes in. But if we have every district for instance they have the psychologist, ehm to take care of them at the whatever level, it would be it will be okay. So they can have enough of those things right from the district level so that if they have to refer, it's either because the kind of either gadgets or things that are required cannot be available at that level then they move them to the regional or national level. But otherwise the district should be equipped to and you realize that when it happens that way, the cases would be seen much earlier than them becoming chronic.

**Interviewer:**

Thank you Sir, I have just one more question in human resources. We touched on seeming parallel relation mental health authority Ghana Health service.

**Respondent:**

Yeah

**Interviewer:**

How are mental health staff managed? The those who are providing mental health services at the community level, are they managing?

**Respondent:**

Oh, let me say that those at community level, they are just like any other like I said they are Ghana health staff, so there is no special ehm treatment for them. They are Ghana Health Service staff. It's just like we have a bunch of community health nurses. They say they want to do post basics; some go to do disease control, some go to do nutrition and some go to do psychiatry. So when they come back, there are community health nurses who have done post basics and so you specialize as disease control officer so join the disease control unit, you psychiatry join the psychiatric unit and you nutrition so join the nutrition unit.

**Interviewer:**

So there is no special distinction?

**Respondent:**

Yeah

**Moderator:**

Ok, are there any challenges with reporting lines?

**Respondent:**

You know we still compile mental health reports, and it starts just like they generate them from the district level because now our system of reporting is through the DHIMS. So the facilities enter information at their level and it just goes out because we at this level, we don't enter information but we only look to see if what they have entered makes sense and if it doesn't make sense we call them to explain because we can't change it at our level. If you call them and it doesn't make sense, they have to change it. They will correct it and it will reflect here so that's how we take the reports and then like I said, the clinical coordinator who is the regional rep is under the deputy director clinical care so if we are reporting even at the annual reviews and things it's the deputy director clinical care who would present for ehm mental health so whatever information he has that's how it comes out. But I know that the coordinator sends report to some national rep.

**Interviewer:**

Are there any challenges with that?

**Respondent:**

You see what happens is that because it's like they have come to be a coordinator ehm then a emolument wise, they are not your staff.

**Interviewer:**

Meaning their emoluments are different?

**Respondent:**

They belong to the authority [Ghana Mental Health Authority]

**Interviewer**

Even with their emoluments?

**Respondent:**

Yeah, they belong to the authorities because they appointed them. Excuse me I have a call.

**Interviewer:**

Okay.

**Respondent:**

Sorry for the delay

**Interviewer:**

It's okay.

**Respondent:**

Yeah so you know is the Authority who appointed them so they pay their this thing [salaries], so we can say that once it's like that, they can easily cause problems but the way we work here, we have not encountered any problem. Everything seems to be seem less.

**Interviewer:**

Is there anything similar like that happening in Ghana health service where there is a whole structure there and their emolument is not within Ghana health service? Any other group?

**Respondent:**

No, I think all the people under Ghana working authority service, there was a time where ehm what do we call it? I think Global Fund engaged some people for TB and HIV. So they were working on the data and those things. Yeah but most of those people have been virtually absorbed into Ghana health service.

**Interviewer:**

Because my understanding all along has been that, you know, they were more unless ehm ehm ehm..

**Respondent:**

Coordinators?

Moderator:

Coordinators when they were working before authority so my understanding was that it's continuing the same.

**Participant:**

No, no. This were the people appointed, they appointed them and gave them letters. So the truth is that, yes some of them were already psychiatry people but they needed this appointment to show that you belong to the authority.

**Interviewer:**

OK

**Participant:**

So that's how some of them did their things.

**Interviewer:**

And ehm how is the existing legislation, the mental health law promoting community mental health?

**Respondent:**

Well, you know the law basically was to make sure that their rights and things are respected, things are made affordable, accessible and other things. Because the thing is not there, the structures are not there. We don't have a psychiatric unit at those lower levels. I mean structure-wise though the service is being delivered, these community psychiatric nurses, they are either attached to a district hospital or to a big health center but its in the area. So, it's not like they have a place of their own where you can say that when you're walking there you are going specifically to the psychiatric.

**Interviewer:**

So yes

**Respondent:**

But like I said, the laws is just ehn an act which was passed 2012. So it's about 8years and you know the processes, the protocols and things. And even if you look at it, I don't know whether even their families what and things are actually well defined. So you realize that every now and then their CEO would be complaining that no ehm monies have been released to them and those things. So, yes the law is there but it will take some time for it's effect to actually permeate the lower level but I must say that it's a good start and at least is ehh giving the way to follow that they are at least when we talk about their rights and then the fact that people are abusing them and now people can be taken on especially those quacks who try to claim that they're helping them and then ends up abusing them and making their situation worse. And now because there is a law you can be taken on and that seeks to protect them and then also indirectly it also talks about the quality so the quality of care they will be receiving, it will improve once they start putting these structures in place.mm but..

**Interviewer:**

And ehm do you know of any mental policy that's existing and how is it promotion community mental health services?

**Respondent:**

Community

**Interviewer:**

Mental health services at the community level?

**Respondent:**

Policy, hmm, which policy?

**Interviewer:**

Any mental health policy.

**Respondent:**

Just trying to see if I can remember anything. I don't know I don't know but I know there are programmes that are run by other NGOs and other thing which seek to take care of their needs but I can't remember of any policy.

**Interviewer:**

There has been a mental health policy that has been under development.

**Respondent:**

Okay

**Interviewer:**

We were thinking that this government would have formally launch it because it was completed some way back in 2017, 2016 there about

**Respondent:**

Okay

**Interviewer:**

Just for the minister to launch it.

**Respondent:**

I don't know of any one that's in existence.

**Interviewer:**

How about participation of people with mental health problems in service organization. How much is that happening involving people who are living with mental illness in service delivery and how they think the services can be better delivered for them.

**Respondent:**

Oh let me say that, that one is very minimal though it's happening. Ehm I'm saying this because you realize that the community psychiatric nurses most of them operate with the relatives of these clients and so some of them they are the people who keep the medications for the clients. They don't give the medications to the clients so it's relatives who would ehm administer the drugs. They report, they bring them for reviews if there is need or if there any relapse, they are the people who bring those clients to the facility. And some they have telephone communication with the nurses so that if anything is happening in the house that they need their attention, they communicate. So that's the way they involve them but I won't say maybe they come to the facility to assist doing anything but they do this based on their own clients' family family members.

**Interviewer:**

Could that be improved and so how...

**Respondent:**

Yeah, you know for families to be involved, yes we can improve that by giving some of them certain basic acknowledge that they can use to assist the person rather than allowing other people to come and be abusing them because some of the things are simple things they could have done at home for them instead of carrying them to the spiritual homes or some other places. So if we can educate them that is the relatives to understand that, look with the nature of the disease this one can happen and if this one happens do this then it will go a long way to improve the condition of the clients. So educating the relatives, I think can do.

**Interviewer:**

Thank you sir, this is helpful.

**Respondent:**

You are welcome

**Interviewer:**

I want to move to financing of mental health. What finances oh budgets are there for mental health services at the community level? What are the source of funding? Do we even have the funding for mental health services at the community level?

**Respondent:**

I won't say they have but I know occasionally the authorities gives money to the coordinator through the regional director for them to carry out certain either training or service. Whatever psychiatric activity they want to carry out at the community level but there are no budget lines as for a service for psychiatric or mental health activities.

**Interviewer:**

What is it so?

**Respondent:**

Like I said, now they are on their own in quotes, but we know that they have not reached that level where they can have all this. Because, like I said from the law, you can realize that a flow of their funds is yet to be determined how the processes are still ongoing. I don't know if they have finished now but I know some years back they were still having that challenge. So if those things have still not been addressed then that is why they are not getting that. Because I don't know whether, because they also realized that they say they have staff of their own so if they give them any by that, then that becomes a duplication. I suspect those are issues not until they are able to address and see that. look when we are talking about mental health that is mental health so this is mental health hospital, this is mental health staff then it would be difficult to do that.

**Interviewer:**

So if occasionally, they send something to the regional coordinator through the regional director then they must be getting some funding. Do you know the source?

**Respondent:**

Oh, this one that came that was some years back I was in Greater Accra then. I know it was government that allocated something to them, though I know that there are some other NGOs like you people, you were supporting them. Apart from that, I don't know any other official source that they get their money but I know there are few NGOs that are supporting them and you realized that in some of the cases the NGOs is not, we can't say it's universal they just go to a particular region and support some people while in some place they maybe getting something to do based on the interest of the NGO but some regions don't have that advantage.

**Interviewer:**

Okay, are payment made for mental health services?

**Respondent:**

Oh, currently those that the community psychiatric people take care of are not supposed to pay.

**Interviewer:**

So do you know if payments takes place?

**Respondent:**

No complains have come to me to that effect but I know if they are taking, the people would complain because like I said they are already poor and they are relying on somebody and so those other people would complain especially when they know that mental health care should be free.

**Interviewer:**

A few people have talked to so far especially the people with mental health problems, family caregivers and they talked about them paying for the medicines because the medicines are not available and so they have to prescribe it for them to go and buy at the pharmacy.

**Respondent:**

That is the challenge.

**Interviewer:**

And how is health insurance working in mental health care?

**Respondent:**

In fact I don't know what to say. in fact I need to take a second look that was my intention, I have not checked if they have included their drugs into the health insurance. I don't know about that, because this should be one on the categories that they should be considered.

**Interviewer:**

Okay, so you have no comment on that?

**Respondent:**

Yes, I have no comment that because I don't know if they have included yeah drugs but the only thing I can say is if they can include that then it would be a big relief for the he relatives because the patients themselves cannot buy the medication because the serious ones can not work so they are not earning anything to even have money to make expenditure.

**Interviewer:**

Okay. And ehm how are medical products especially medicines and related equipment or devices to support mental health care service the way the community level planned for and procured and distributed.

**Respondent:**

They are like any other ehm unit psychiatric nurses and then those at the hospital and things. when you come to planning, they also plan and bring but some of those things are sent to the higher level because like I said they would send theirs to the authority and how they acquire this things I don't know because hitherto, the medicines and things go through, ehm the allocations they were bringing specifically for mental health which we just get and download to them, because, it's just like we have programmed that TB, they buy them nationally and then distribute to the regions and then regions to facilities and it goes down. it's the same way their drugs should be coming but they are not forthcoming.

**Interviewer:**

So when you are doing your annual programmes or budgets, do you plan to procure such tropic medicines?

**Respondent:**

Some of the hospitals do but most will not because if you go and buy you will not get re-imbursement. That's why I was saying I need to check of they are included in the health insurance because I remember some time ago there was a team which came here and we were saying that we need to advocate for that but I don't know if they are included otherwise if it is the hospital will not hesitate to tell is because they know they will get back their money but as we speak now I'm not aware it's part of the health insurance package. So most the facilities we would not because some of the drugs are quite expensive looking at the fact that some of them you need to use them for a long period. The price maybe small but if you look at the quantities you need, it end up being big.

**Interviewer:**

So we touched on information technology

**Respondent:**

Yeah

**Interviewer:**

I want to revisit it. We talked about basics too. So what kind of information about mental health care services is collected at the community level and how is it processed and used.

**Respondent:**

The information acts is like before the games people go into communities. We try to look at how many cases are we going to classify as mental health cases so we look at that and categorize them. So if you look at the report of mental health, they would talk about epilepsy, drug abuse, schizophrenia and various categories. They try to find out the numbers. They find out how many of them are on drugs. Those who are getting well, the relapse, those are the kind of things they Pict from that level and as we go depending on the level that they enter over it when you get to the hospital level, their management is higher so they will also but they still also look at the types and the outcomes.

**Interviewer:**

Okay. So is there a kind of feedback process that exist for data information, evidence generation and for decision making.

**Respondent:**

Yeah, what we do here particularly as a region is that it's not limited to just psychiatric. We have platforms where you see either the deputy director public health of clinical care is always looking and sending back to the various facilities. This data is incomplete. You have not completed this form. You know we have data sets in there, if it is not complete, then it's either they do a score board that's those in red, those in green, those in yellow and others you send it back to them. You see the responding we have updated, we have corrected. That's the kind of things we do.

**Interviewer:**

I know the Ghana health service is quite good at performance reviews and others.

**Respondent:**

Laughs...

**Interviewer:**

How does mental health fits in all that?

**Respondent:**

They fit in because they are under clinical care. So once more we are doing that you can be sure that whatever they have done for the period would be reported through clinical care.

**Interviewer:**

Okay. So monitoring and evaluation at the community level in terms of mental health service provision. how are they organized?

**Respondent:**

Oh, this one, it's the coordinator. You know he has some district officers who would go and look at what's happening at the very lower level so that's how it goes.

**Interviewer:**

Okay, is there any research related to mental health currently going on in the region?

**Respondent:**

No. There is nothing like such happening.

**Interviewer:**

Okay, I'm getting towards my last set of questions (laughs...) I want to understand, we have bondage about this term of integration of mental health. What is your understanding of integration of mental health. What is your understanding integration of mental health? Integration of mental health with general care, integration of mental health at the community level. Your opinion, what is integration of mental health services at the community level?

**Respondent:**

Okay, you see for me is another way of abusing the word integration. You see like I have already mentioned there are staffs who are engaged in the mental health who are already Ghana health service staff who either they were doing other things. These are community health nurses who originally, they only go to what? immunization; they go free and come back and now they go with their friends whilst they are doing immunization they are doing mental health. So why do you people now go and say how are we going to integrate or because it is already part of it. Like I am saying if as we speak, mental authority has its structures, like we have CHAG facilities, these are CHAG staffs then we can start talking about the service that we have both and how can we collaborate or bring things together. but this is already happening because if you say at our community level the people who go there are Ghana health service staff, so we cannot talk about integration here. It is an abuse of the word.

**Interviewer:**

Okay, so what you would have been the proper word to use?

**Respondent:**

So you see, if the authority wants to say okay, because we are now authority maybe he wants us to share the structures that existed before their going. Is it that we should start getting worried that oh, let's remain as an integrated body and you can have your saying or we would let your staff, this staff be here, they would be accountable to you but when it comes to service delivery then the two to see to it. But we don't have that now, because as we speak now, apart from the coordinators, all the other lower level payer's are paid by the under Ghana Health Service payroll and so they are already working for Ghana Health Service.

**Interviewer:**

So this has always been the tricking issue ehn? There seems to be an admission that yeah mental health have been disadvantaged, they are not saying neglected. mental mental health have been disadvantaged somehow for quite some time but we are also looking at the issue of integrated it's then you would get the response that, oh look, everything is already running there is nothing else going on about it.

**Respondent**

I think I think what they should rather talk about is how can we improve or make mental health services more visible. Is the visibility that's not there because they, as for the provision of those services they are there is just like neglected tropical diseases. they are diseases just like the other ones but you see our attention is being paid to malaria, measles this, chronic ulcer and those things. so people have now gone to rebrand it and say neglected chop-guard disease and have given this name which is appealing to people and they are ready to sponsor them too. So now you see people talking about neglected tropical diseases. They making those diseases more visible creating more awareness for them. Maybe I would say facing a similar thing where the attention is not enough, so how can we increase or bring it to the fore rather than saying integration. You can’t talk of integration when something is already part of something. Because if the community health nurses are going out, they go together unless she has a case that she has been called specifically which she will go for but otherwise it is like how outreach. We are going to this area to operate whilst you are working, you are looking out for this. So for me I don't.

**Interviewer:**

Now, one of the health system, health system blocks is leadership and governance.

**Respondent:**

mmm

**Interviewer:**

How would you describe the level of this leadership of mental health at the community level, doesn't it exist?

**Respondent:**

Alright, if you want to talk about leadership and governance at the community level, we need to look at it. In the community, the chiefs and elders are the people who are in charge of the, the community though we go in there to provide health services so you realize that some of the things you do there you may have to seek permission from them to check because he just said this person is mental health so do this. You should be able to convince them that ehh something can be done about it, in a different way from what they are doing. So in some of the communities, depending on how it is organized. the opinion leaders have a say so you need to actually deal with them before you can ehh because some of them because of their beliefs especially much smaller communities. Because of their belief, you can just go there and say that this one is mental health you are going the treat because the chief priest or somebody has already defined what must be done and you can't to go again that. So those things are there. But when it comes to ehh our structure then you would say that yes, the psychiatric nurses in charge at the community or ehm subdistrict level when it comes to psychiatric issues and is been supervised by the district psychiatric person who is also under the district director then from there, it comes to the regional level where the coordinator is now in charge of supervising the district. And then further to the subdistrict level and reporting to clinical care director who would inform the regional director and then the coordinator also has the responsibility to report ehh parallel to the authority. So as you are getting to the upper level then the governance seems to branch off because then the coordinator becomes ehh, have divided attention. He must satisfy the Ghana Health Service, he must satisfy the Authority.

**Interviewer:**

Is it working?

**Respondent:**

laughs.... We cannot say there is no leadership and governance of mental health in place at the Primary Care level. Increasingly, directors are taking an interest and through that the situation will improve. Well, like I said yeah, they are busy attending to the patients, they bring reports and whatever report they send there we are not worried because ehn occasionally they send them money to carry out some of the activities anyway so you must be accountable. so if somebody [referring to Ghana Mental Health Authority send you money you can't say you're giving report somebody else. And the fact that yes, our [GHS] people are engaged and they [mental health staff] use our facilities that's why we are also interested in looking at what are they doing and how are they doing it. yeah.

**Interviewer:**

So ehh you did mention that maybe perhaps one of the reason we should be looking at is to give visibility to mental health but it is not an issue of integration as said.

**Respondent:** Yeah

**Interviewer:**

but what else can facilitate, what opportunities are there to develop mental health at the community level than is currently the case?

**Respondent:**

Oh you see what do we call it? If you look at the way ehm mental health have been at the this thing. the one, if we can make sure that if we say mental health should have its care unit then we must have their staff well defined and resourced appropriately. Because if you have to be depending on somebody to do somebody's else work then your ehh you are not likely to give out your best because you can have plans which if they don't ehh fit into what the others want to do because if you are to go out, in using your common car and these people for one reason or the other will not go then you may also end up carrying out your activity. So, they must look out clearly for that service and resource it to the people who are actually running the affairs must be resourced too to carry out their activities. so that if it is outreach, they want to do, they can go out, if it's durbars they want to do, they have the means to carry out durbars and then they would look at them.

**Interviewer:**

So, what's your opinion on the growing international or even in country call for improvement in mental health care services?

**Respondent:**

So that one, like I said it is call in the right direction because you see ehh some of these things is not things they brought onto themselves. It is just unfortunate situation that some of them found themselves in that state. So we must treat them with dignity ehh because they are still humans and especially when some of them we give them the quality care they become they can come back themselves and be very responsible. So I think such a call to respect their rights and dignity and give them the best of care is the right direction.

**Interviewer:**

Are there any key strategies you think you can comment that can support development of mental health care at the lower level to the committee level?

**Respondent:**

Hmm ehh first like I said now you see we have the capacity we have there when you come to mental health it is limited. That is why I was saying if like you said ideal situation if we can get more ehh should I say qualified or yeah more qualified personnel or human resource to operate at the district level then people would see results faster and people would get to understand that oh these things they also just need ehh specialized doctors or healthcare personnel to have their situation changed and you realize that ehh the patronage of the facilities would change and with time it would no longer be on issue of stigma. If we manage to do that, that you stigma would automatically just go away because ah this person that has been there so it is just a sickness but if you don't have, you are only using nurses to go and talk to them that this can be done, this can be done and the condition is still the same, then you're not encouraging anybody to even come to the facilities, so the best thing is that let's get more qualified people at the lower level who can actually address the issues because people like seeing results people like seeing results.

**Interviewer:**

And what would work against this kind of approach, what are the likely.

**Respondent:**

My friend you know me and you know already (laughing). You know to get some of the people to those level, to get human resource to get there that would be a big challenge otherwise we can start first by introducing these mobile clinics where you have senior officers, psychiatrist and you go and spend a week in a community or something to manage cases and move out and then people follow up to go and do reviews and things and if people are seen there, you would be surprised some of the officers who go out, some may develop the interest let me go to this area because there is work to do. it may touch some of them that's how we start getting them dotted round rather than the emptiness we have now. yeah.

**Interviewer:**

Wow I am almost done with virtually with my questions if there is anything you want to add that would enrich this discussion that probably we have not touched, I would be happy to hear it.

**Respondent:**

Oh, mental health issues I don't think, I think we have talked most about it. The only challenge some of us had was the issue of the creation of authority without having ehm developed structures below it. that's the only concern some of us had but if you have ehh an authority who rely on another organization to prove that it's working, then there's a challenge. So I do to know how soon they intend to make sure they develop authority, mental health authority. If they, the earlier they do that, the better for all of is.

**Interviewer:**

I was talking with one of the Ghana health service headquarters staff and one of the things she mentioned was that ehh you should also depends on a lot of the leadership(s) across the regions and districts with regards to what level of interest they have with mental health. How do you see that?

**Respondent:**

That one is a non-starter. You see the issue is that we have people who have trained as in mental health, if you say you have created a mental health authority, you have a regional mental health coordinator and you feel that you want regional directors for mental health people would apply or you don't believe so? people would apply to take that position because some of them are just crowded in facilities in Accra so if they have to come and be in charge of mental health in a region at their regional level, they would be happy. Because its a different authority altogether. Its a different body from the Ghana Health Service because they would be solely, it's just like we have the Teaching hospital, they have their CEO. Originally, we were together and then they created Teaching hospitals have they not gotten CEOs for them with a lot of staff? its the same thing. so its not like people are not interested in this thing. So they shouldn't start complaining about whether people create it and you will see people taking up the challenge. yeah. So as for the excuse that whether people at the regional level will show interest that's a non starter. I don't think those are excuses that should not be accepted.

**Interviewer**:

Okay, laughs... and then do you have any question for me with regard to this study ehh I know I may be able to answer but, laugh...

**Respondent:**

Okay, so maybe this is a usual thing at the end of it you have written, you have found out some key issues, how are you going to make, ensure that they are implemented.

**Interviewer:**

Ehh actually my quest is that it would be a source of reference for advocacy depending on the quality of the end product . ehh it could also serve as some reference document for mental health services in Ghana.

**Respondent:**

Okay

**Interviewer:**

so it depends on how I am able to produce good thesis

**Respondent:**

Okay

**Interviewer:** Worthy of somebody trying to even use as a reference and then being in the sector, I hope it would also broaden my own understanding of things so that when I am speaking, it would even better for my, better informed position than now.

**Respondent:**

laughs.. So do you have any background in ehh in mental health?

**Interviewer:**

Not at all except working in it for close to 16, 17 years now. I would not attempt to diagnose and treat but I know a lot about schizophrenia, psychosis, depression and some few disorders. I know a lot about medicines, Haloperidol, Olanzapine, Amitriptyline etc. but I will not attempt to diagnose and prescribe treatment for you.

**Respondent:** Yeah, okay

**Interviewer**

But as it is the case I see myself a public mental health service specialist.

**Respondent:**

Okay

**Interviewer:**

I have gotten knowledge a bit of what about mental health policy and services. In fact the masters from this...1: 13: 18

**Respondent:**

okay

**Interviewer:**

yeah, and I have been involved in a lot of the WHO mental health prints.

**Respondent:**

Okay

**Interviewer:**

MH Guide, Intervention Guide.

**Respondent:**

ehm, I think they are going to do a training..

**Interviewer:**

Yeah

**Respondent:**

Next week

**Interviewer:**

Next week. In Bolga I was there to observe the Bolga training.

**Respondent**:

Yeah

**Interviewer:**

And I would be in Accra next week for the Accra training.

**Respondent:**

Okay

**Interviewer:**

So I have quite some way of understanding of what it looks like but knowledge you can never have all the knowledge.

**Respondent:**

Laughs, we are always searching.

**Interviewer:**

We are always searching to improve upon.

**Respondent:**

The consent forms how many are they?

**Interviewer:**

ehh they are too. Information leaflet and the consent form. The information leaflet says you have read and understood, the consent form also say you have accepted to freely give the information.

**Respondent:**

Laughing...

**Interviewer:**

(laughs too) So thank you so much I would stop the recorder.

**Respondent:**

Okay.

| **Name of Interviewer/Facilitator** | xxxxxxxxx |
| --- | --- |
| **Name of note taker** | xxxxxxxxx |
| **Type of participant** | Health Professional (xxxxxxxxxxxxxx) |
| **Gender of participant** | Not indicated |
| **Disability of respondent(s) (physical or psychosocial)** | N/A |
| **Age of participant** | Not indicated |
| **Community Name** | Not indicated |
| **Interview date** | Not indicated |
| **Duration of interview** | 28min. |
| **Recorder number** | VN870107.MP3 & VN870108.MP3 |
| **Name of file (follow convention below)** | Transcript individual interview. Mental Health Professional.docx |

**Introduction**

This individual interview was held with a Health professional (Acting xxxxxxxxxxxx Regional Director) at the Ghana Health Service in the xxxxxxx region of Ghana. The participant was selected for this interview because he is a health professional. The interview was in English. A digital voice recorder was used to record the discussion and which were complemented by handwritten notes. The details of the discussion and written up below

**Interviewer:**

Thank you for the permission granted me. My name is xxxxxxx, I'm doing a thesis research on integration of mental health care at the community level in Ghana the role of the health system framework of the WHO. As a leader in the region and one of the regiment that am carrying out the research I wish to get your perspectives on this eh subject. So...

**Respondent**

Yeah

**Interviewer:**

I wish to kindly ask you to (silence, Participant having short conversation with another) so if you could kindly introduce yourself, your position and ehh what you do on a day you day basis and for how long you've been working in this role.

**Respondent**

My name is xxxxxxxxxxxxxxxxxx, Am currently the xxxxxxxxxxxxx of health services for the Bono region. My role in health service delivery is actually can be found at three levels, As a regional director, I actually help the national, Ghana health service national headquarters in coming up with policies and guidelines for the service. I also, I report to the Ghana Health Service on daily basis as to health activities in the region. I also report to the Regional Minister and then the regional health committee as to the day to day procedures on service activities. I also give report that's what I said already to national, Ghana health service national, then I make sure I disseminate explain and disseminate the activities and policies to the district levels. I plan and draw and assist the districts to implement the policies of the Ghana Health Service. I also make sure that prudent management of the resources since the resources are limited, prudent management of the resources at the regional level and also guide the districts to do likewise.

**Interviewer:**

Thank you Sir

**Respondent:**

We resolve issues at the districts level and we also make sure there is harmony between the staff working at the various districts, we build up capacity of the staff by implementing policies and workshops that are geared towards of quality of care and also geared towards prevention of diseases, all sort of diseases. Really it's my vision to make sure that preventable and avoidable deaths are brought to the minimum to the very minimum using the structures in the Ghana Health Service.

**Interviewer:**

Thank you so much

**Respondent:**

Basically that's my role

**Interviewer:**

Thank you thank you. I will be touching on this because ehh I want to explore Mental Health Service provision, human resources, legislation, financing, ehh research development, information, data generation, leadership and governance, and integration of Mental Health Services.

**Respondent:**

Good

**Interviewer:**

So I wish to start with the services. What services are available for treating mental health conditions ahh in community based health facilities in the region?

**Respondent:**

The Ghana Health Service has a structure and the unit of the structure is the CHPS concept and in this region we've made sure that, almost all the sub units or subdistricts have mental health personnel and which have been termed as community mental health personnel taking care of the CHPS or taking care of the communities. So it’s, the that idea is cascaded or is replicated at the community level the ehh eh this thing what do you call it the district level, no from the community we come to the sub-district level, the district level, now to the regional level and we've paid particular attention to that and make sure that even at every facility at the CHIPS compound we designate a bed for the mentally ill. The sub-district a bed for the mentally ill, the district hospitals they are advocating that they have at least four beds for the mental health, dedicated to the mentally ill. And then at the regional hospital level, ten to twenty beds. Currently at this regional hospital we have about twenty beds capacity that have been designated for the mentally ill patients and the structure is such that we have a mental health coordinator for the region and every district likewise in this region is also being manned by a mental health, district mental health coordinator. That's the governance aspect and they actually control or delegate and manages the mental health activities at the district level supervising the sub-district who intend also go to supervise the CHPS compounds. So the structure is well laid and we receive report, I receive quarterly report from the regional Mental Health coordinator and during every annual report, and he is featured and he brings about their challenges so far as mental health care is concerned, so far as mental ehh quality of care for the mentally ill is concerned. Well to add it to, I'm the chairman for the Region Mental Health Committee; so we came up with a solution that ehm all the committee members had districts allocated to them as parents, so they are parents of some districts where they will go around look at the mental health situation in those districts and come and report to the mental health committee at the regional level when we ever have a meeting. Also we were in collaboration with all the faith based facilities as well as non-governmental ehm institutions and healing places, eh places of healing. Whenever we have meeting, the Mental Health Committee has a meeting we make sure we invite people who deal with ehh, who use herbs, the herbalists and the faith based healers to our meeting annually whilst we listen to them, listen to their challenges and in so doing we also impact to them. The fact that they should deal with the mentally ill with respect and the fact that we should have a collaboration with them, we show them that there is a ehh there are two aspects of the mental health some being very physical and will need health care at the health facilities and the spiritual aspect they can take care of that. So we encourage them to refer their patients to us and we try to tell them not to abuse the mentally ill by chaining them to trees and sometimes starving them, not giving them food for days and sometimes even trying to beat out according to them, the devil from them because they think the devil has possessed them that's why they are mentally ill some instances. In this region also, we also came out with a suggestion actually, it wasn't a suggestion it was a proposal, a written proposal budgeted for and everything to really take all the mentally ill patients from the streets. We were basing that on the fact that research had shown that 80% of the mentally ill patients who are walking on the streets can be cured and reintegrated into society. So based on the findings on that research, we put up a memo and we drew out activities to take out the mentally ill from the society in bits.am sorry to say that we never had funding we sent the proposal all over the place but up to now we've not had the funding to kick start that proposal. The idea was to take, you see this region have about 30 something mentally ill patients, we counted them and then we look, we are located or we look out for wherever they are staying to renew their sleeping places renew their eating, renew where they go to rest in the night, so we were going to use that to with the you know the ehh the the necessary laws backing us , and the police collaboration we were going to take them out of the streets about five at a time at the regional hospital we treat them, and when they get better, we move them to where we designate as a half way home where they will be taught trade and other aspects of reintegrating into the society and from there you know through the community health nurses were going to send them back to communities that they come from. Having used the community health nurses, to engage the communities in durbars, to actually make sure stigmatization is minimized or eradicated completely before we send the patient in and also to make sure that the family in which they are going actually embraces them back and takes them in as if they were like people who had just had any other disease like malaria and they have been cured of the disease how we receive they receive them back. We had all these plans in place but is unfortunate that we have not been able to ehn really push forward the plans because ehh all the institutions were sensitized the police, the army, the market women, the teachers, everybody RCC ehh this thing ehh the veterinary, forestry, they were all sensitized as to the plight of the mentally ill and the need for us to take the mentally ill patients as people who have suffered any other disease like malaria and so on. So that's what we do.

**Interviewer:**

Thank you so much Sir. I'm very grateful, that's very comprehensive. Sir, I want to focus on the eh community level and I guess that's CHPS and sub-districts level

**Respondent:**

Yes

**Interviewer:**

What exactly are the mental health services provided at that le, at those two levels?

**Respondent:**

Those two levels, we provide creative as well as preventive services at those two levels. As I was talking, I mentioned durbars, normally durbars are created you know at that level and education given to the community. As to what is mental health? what are the ramifications of mental health?, what are the inducers of mental health? and so on. So those are the services provided and then also, at the community level those who have been treated; who are known to be having mental illness and have been treated and are in the community, the community health nurses and the community health officers make sure that they do no you know, what is the word?,ehm they did not, they always take their medication and do not to and they do not there's a word,

**Interviewer:**

default

**Respondent:**

ehhm default from their medications and also we sort of sometimes give pieces of advice when we see that one particular patient is defaulting and maybe going back to the old habits we call them in and give them specific advises. And also when there is acute sicknesses mental health sicknesses those are the people at that level, they've been taught how to give them medication you know to bring the ehh so we call the acute illness as some sort of emergencies you know we've taught them to actually give them medication that would calm them down and then refer, so the referrals system takes place between the CHIPS compound to the sub-districts, the sub-district to the district hospitals who so if unable to manage, they also bring it to the regional hospital, regional hospital we have, even though we don't have a psychiatrist, we have mental health officers and psychologist there who will put the patient through. And we also have the services of psychiatrist from the ehm this thing the Teaching Hospital who normally Kumasi ehm Okomfo Anokye who normally comes to visit us to render services. So those are the services that are rendered at the sub-district and district level ehh and then the CHIPS compound with the help of CHOs and then community mental health nurses.

**Interviewer:**

Thank you Sir

**Respondent:**

mmm

**Interviewer:**

Very clear

**Respondent:**

Alright

**Interviewer:**

So ehm you've talked about a bed for the CHPS and a bed at the subdistrict level and then ehh extra beds about four to five at the district

**Respondent:**

Yeah, yes

**Interviewer:**

And over twenty at the district level

**Respondent:**

Yes

**Interviewer:**

With this approach, is it based on on ehh a certain document or policy requiring of ahh the various health managers to comply or is just based on what you see is appropriate to do?

**Respondent:**

No, no there was a document, a document was sent that stated that all regional hospitals should have between ten and twenty berth capacity allocated for mentally ill. District hospitals have up to five berth capacity and in every community at least there should be one berth allocated for the mentally ill. So is not from my whims and caprices that am bringing that, there was a document sent from the this thing ehm Mental Health Authority, in fact they brought that when they were looking at the ACT 526 or so for the Mental Health act when they brought it and they were disseminating the contents of the ACT.

**Interviewer:**

mmm

**Respondent:**

that's when I might have gotten the number, the figure a little

**Interviewer:**

846?

**Respondent:**

846 yeah. I know is 8 and there's a 6 but what was in the middle (both laughs) 846 yeah. When they brought it, and they were asked to disseminate the content,

**Interviewer:**

Yeah, yeah

**Respondent:**

A letter came also informing the region to make provision for all this thing.

**Interviewer:**

Okay, how about the levels of service provision from CHPS to subdistrict to district to regional. Is there a document or guideline like standard treatment guideline available?

**Respondent:**

Well, ehh you just a minute. (phone call)

**Interviewer:**

okay Sir

**Respondent:**

Mm him so the level of service provision have been spelt out as to what can what type of drugs can be given at the levels of CHPS compound, the subdistrict, the district and then at the regional level. I think they have the documents to support it.

**Interviewer:**

Do you have a copy?

**Respondent:**

No, I don't have a copy. But I think the regional mental health coordinator would have such a copy and it explains how and even the staff who are there have a limit, they have a limit to the way they structured their education. We have the community mental health nurses and from there we have the mental health nurses themselves, mm then we have the mental assistants. You see? and then we have those who have diploma in mental health

**Interviewer:**

Services

**Respondent:**

And I think we have those who have, even, degrees, mmm?

**Interviewer:**

Yes Sir!

**Respondent:**

Before we come to the psychiatrists who are medical officers themselves and unfortunately, we don't have them at the district level, but some regions have them but we don't have them in this region. We use to have one man who died hmm Dr Jones, I think you might have heard of him.

**Interviewer:**

Yes Sir

**Respondent:**

Techi Jones

**Interviewer:**

Yeah, Techi Jones

**Respondent:**

mm yeah

**Interviewer:**

Thank you Sir, so how accessible are mental health services to the population in the region?

**Respondent:**

I think in this region we've done a lot and mental health service is as accessible as any health service delivery. Is they are all along the lines I told you about the CHPS which is in the communities then the sub-districts mm then the district level. So every you go service is available

**Interviewer:**

mmm

**Respondent:**

And it's accessible

**Interviewer:**

mmm

**Respondent:**

You see? and even we've gone further to make sure that ehm we minimize the financial challenges associated with accessing health in general by making sure that ehn all the mentally ill patients have been enrolled onto the National Health Insurance we did that district by district and I think some MPs, here I must say that some MPs did well help out in giving out the money to enroll them onto the National Health Insurance.

**Interviewer:**

mmm

**Respondent:**

So mental health is accessible as any other health service delivery. But I must quickly add, that the knowledge in the communities as to how accessible mental health is not actually there even though we've done a lot we've done a lot (someone walks in).....

| **Name of Interviewer/Facilitator** | xxxxxxxx |
| --- | --- |
| **Name of note taker** | xxxxxxxx |
| **Type of participants** | Caregivers and people with mental health and psychosocial disabilities |
| **Gender of participants** | Females |
| **Disability of respondent (s) (physical or psycho-social)** | **N/**A |
| **Ages of participants** | 35,64, 34,53, and 35 respectively |
| **Community name** | Not indicated |
| **Interview date** | 24^th^ August,2020 |
| **Duration of interview** | 1hour 12 Minutes 3Seconds |
| **Recorder number** | VN870100.MP3 |
| **Name of file (follow convention below)** | Transcript individual interview. Caretakers of mentally challenged clients. |

**Introduction**

This interview was held with caretakers of mentally challenged patients at Sunyani in the Ahafo region of Ghana. The participants were selected for this interview because they had people they were caring for who were mentally challenged. The interview was conducted with both English and Twi language. A digital voice recorder was used to record the discussion and which were complemented by hand written notes. The details of the discussion are written up below.

**Interviewer**

Thank you very much giving me the opportunity to have this conversation with you. We apologize for keeping you waiting till this time. What we are doing is a research trying to understand community mental health within the health system in Ghana Ghana. And how mental health delivery at the lower level, primary care, community level can be better enhanced. Because of that we are asking a whole lot of people. From people who are using mental health services to people who are caring for mental health patients to people who are providing mental health services to people who are managing mental health services and to government officials and so on and so forth. So now it is your turn, there's no wrong or right answer. All we want is to understand their experience and what they know. Because they say experience is the best teacher. So we cannot sit down and write the report without talking to people like you. So, errhh, to start the conversation, we will like each of you to introduce himself or herself. Your full name, if you know your age you tell and who you are taking care of and what the condition of the person is. So I will pass the microphone around. And because of the nose mask it will be better for you to speak louder.

**Respondent**

I am xxxxxxxx, 35 years of age I am here because of xxxxxxxxxxxx. She has a mental illness. She is 15 years of age.

**Interviewer**

Do you know the name of the condition? Is it psychosis, schizophrenia or depression?

**Respondent**

She has depression.

**Interviewer**

How long has she been suffering from this?

**Respondent**

Since last year

**Interviewer**

Okay.

**Moderator**

Madam please your name?

**Respondent**

I am xxxxxxxxxxxxxx, 64 years of age.

**Moderator**

Please who are you caring for?

**Respondent**

I am here because of my son by the name is xxxxxxxxxxxxxxx. He is also 34 years old. He is mentally challenged. He had it in November 2016

**Moderator**

So that's about 5 years.

Madam, please your name?

**Respondent**

I am xxxxxxxxxxxxx

**Moderator**

How old are you please?

**Respondent**

Am 34 years old

**Moderator**

How old is the person you are caring for?

**Respondent**

Her name is xxxxxxxxxxxxx

**Moderator**

How old is she?

**Respondent**

15 years

**Moderator**

15 years?

**Respondent**

Yes please

**Moderator**

Please what is wrong with her?

**Respondent**

She suffers epilepsy

**Moderator**

For how long now?

**Respondent**

For about 5 years now

**Moderator**

Please your name?

**Respondent**

I am xxxxxxxxxxxxxxx

**Moderator**

How old are you?

**Respondent**

I am 53 years

**Moderator**

What is the name of the person you are caring for?

**Respondent**

Donkor Moses

**Moderator**

How old is xxxxxxxxxxxx?

**Respondent**

35 years

**Moderator**

How long has he being sick?

**Respondent**

For about a year and half

**Moderator**

What is wrong with him?

**Respondent**

He suffers mental illness

**Moderator**

Do you know what actually his condition is?

**Respondent**

I know he is mad

**Moderator**

You know we have something like malaria as a sickness. Can you tell what his is please?

**Respondent**

No please

**Moderator**

Thank you so much. So the first question I like to ask is, when they come here, do they all get their services here?

**Respondents**

Yes please

**Moderator**

So when you come here, what kind of services do you get?

**Respondent**

Please, when we come to meet them here, we give out our cards to them so they get us our folders. My first time coming here, they asked my name, where we came from and what is wrong with him. The doctor asked many questions and he answered them but there were others he answered inappropriately. When we were done that we were given drugs. We were asked to return a month later after completion of the drugs given. When we returned for the third time that I was told I could come in place of him to take the drugs. Since then, I’ve been coming to take it for him and there has been much comfort in this rather than coming with him.

**Moderator**

Do they do any temperature check or laboratory tests for them?

**Respondents**

No please

**Respondent**

For my own, when you try it he can injure you.

**Respondent**

With my case, after I came for some time, I was asked to then be coming monthly to take the drugs without the patient.

**Respondent**

I was also referred to the regional hospital to do some labs after which I was asked to be coming for the drugs on monthly basis. At times the patient herself comes for the drugs.

**Respondent**

Can’t you help us with issues such as laboratories and other things because some of us travels for distances before we can access those things?

**Moderator**

They didn’t give you a form to go for the lab?

**Respondent**

Yes they gave me a lab form and I went for the labs

**Moderator**

Do they do it anytime you come here?

**Respondent**

Not every day, from the beginning they gave me the labs form and I went and paid some monies.

**Moderator**

So that was at the start

**Respondent**

Yes please

**Respondent**

My own when we started, it was around his knee whenever he wakes up. So they referred us to the hospital. and the doctor requested we do some investigations and from there that they detected that the brain has developed a fault. They check blood pressure whenever we come around and other investigations of which we have to do others outside the facility. When it happened that way and we met the doctor that he requested we bring him to Kumasi so we could take a picture of the head once again. He also said it is a little costly and because of financial difficulty we've not being able to do that. He also added that we should come back to that hospital if we are not able to do what he said. But for the exercises we've been going every month to partake.

**Moderator**

Do you bring him here for drugs?

**Respondent**

I've been the one coming for it after we came for some consecutive times.

**Moderator**

Do they provide them other kinds of education apart from medicine?

**Respondent**

Yes please.

**Moderator**

Do they give you education concerning the condition and how to improve it?

**Respondent**

Yes please

**Moderator**

What do they talk about?

**Respondent**

Last year when we came, they taught us how to manage him at home and the things that he needs to dissociate with.

**Moderator**

Did they talk about the condition the last time you came, concerning what you need to do and what not to do?

**Respondent**

When we came, Gideon like this he takes weed. At the beginning, they told us the weed would help him but all to no avail. He can’t stop taking the weed and the cigarette. But he doesn't know how to take alcohol. If you mention it too, he denies it saying he doesn't know how to take those things except malt. So, if you could help me put an end to this.

**Moderator**

Okay, thank you so much. What I want to also ask is, how available are mental healthcare service? Does anyone who require mental health service get it based on your experience?

**Respondent**

No please because we don’t have access to one at where we are from. We are at Sehwi, Kyereponi border. We’ve been coming here since and after some time I was told I could always come for the drugs on his behalf. So I now come every month to take them for him.

**Moderator**

Which part of western region?

**Respondent**

Sim, Dwaaboso border.

**Moderator**

Okay .how about the general public? Do the general public know where to go for medical service?

**Respondent**

I think it is not all who know. Some don’t even know we have hospital to send them for treatment while others send them to church premises to be catered for. But those who are aware there's a hospital are those few who send their relatives for care. That’s what I also know.

**Respondent**

Please oo, I came with a sibling and the sibling been here before she asked that we come here. And she then brought us. I had not been here before but we say a nurse immediately we alighted and asked her where they cater for the mentally challenged people and she asked that we come with her for direction here and that's how we came.

**Moderator**

Thank you so much. We will like to move on, we will like to talk about the medical staffs catering for the patents, do you know them?

**Respondent**

Yes please

**Moderator**

Those you been meeting who do the consultations and other services, do you know who they actually are?

**Respondent**

Please, we came to meet her here and we know she is a nurse.

**Moderator**

And as a nurse, do you know the kind of work she does in particular?

**Respondent**

As here is a psychiatric health Centre, I know they are also giving nursing care to the patients. But when I always come to meet them that way, I normally greet them and now inform them that I came for a folder and anyone that will come, I will know he/she also helps provide care here.

**Moderator**

So do you know that this is the work that this person does and so on?

**Respondent**

I know that the fact the she is in the mental hospital, that's what actually she is trained to do.

**Moderator**

Next please

**Respondent**

Me when I come, I known that all those sitting at the table are nurses so I provide them with my card and they reach for my folder for me. One will then get up without delay to get your folder for you with immediate effect. After which they will ask your reason for being there. You will then provide them with the reason why you are there. They will have to check blood pressure and temperature other petty things then you will be asked to go and see the doctor. The doctor will then ask what has been happening to the patent and after the information is given, then he will write drugs for you. Those that you are to buy will be purchased outside and those to be given will be given you from the facility.

**Moderator**

So you hold on and we will come back to it. Have we met a psychiatrist? Are they in this facility?

**Respondent**

What I know is the doctor is the psychiatrist to care for the child for me.

**Respondent**

What I also know is Doctor George, he is the doctor I know at Sunyani here. Because when the sickness came, by the grace of God and the love of this doctor for my child he has been able to do a lot for us. I will recommend if there's any award to be given, you give it to him for he has done a lot.

**Respondent**

It is not been quite too long since we started coming here. Nut truth be told, when you meet the doctor, how he attends to you makes you relax and brings smile on your face causing you not forget of some problems. Frankly speaking I don’t know of any doctor apart from him so whenever I meet him, I accord him with the needed respect.

**Respondent**

Me too it is the same Doctor George I know as my colleague before me has said. When we came, we met many nurses who gave us a warm welcome and before you meet the doctor you are already relaxed and feeling okay. They are actually good in providing health care here.

**Moderator**

Have you heard about clinical psychologist?

**Respondent**

Please, whenever we come here, all education and guidance concerning your relationship with the person is always given by this person so as to prevent a relapse or aggravation of the condition.

**Respondent**

Me too when we were admitted at the regional hospital, the doctor came and really spoke concerning my child's intake of weed. He also gave me a certain woman who also gave advice. Its her name that I’ve forgotten. She spoke with him for more than an hour of which I thought my son will not even take the weed again but he still does. Since I started coming here after we were discharged from the regional hospital, how to even give him the drugs, he claims he is well. I've also worked in a health facility before so am aware psychiatric medications are taken for quite a long time and so if he claims he is well, he should be there. So how to get him take the drugs is between myself and my God. So anytime we visit the health facility, the doctor insists that we let him know if not it may become worse but am always like oh no, it won’t be a problem because the person will not desist from taking the weed and cigarette. I am also a female staying with her. So if I don’t take care and he hits me with something, what will I do and for that reason I do my best to let him take his drugs. The doctor keeps saying I should let him know though but am always like when it is time for God in his own wisdom to let him know, he will and will heal him.

**Moderator**

Thank everybody. Do they know the kind of professionals who should be taking care of the mentally challenged patients?

**Respondent**

The last time we came to take drugs that he said some NGO’s came to assist so the drugs are given free of charge. Some NGO’s also assist the government to provide to us the drugs for free.

**Moderator**

Does anybody know?

**Respondent**

As for me the only thing I know is the psychiatrist, I don’t know any other thing aside this. So if there is any other then you have to let us know because am not aware.

**Respondents**

All of us don’t know, abi it is not our fault

**Moderator**

Do they know how they are trained?

**Respondent**

I can’t tell,

**Moderator**

So who oversees these professionals?

**Respondent**

What I know is Doctor George is their head

**Moderator**

Do you know who oversees Doctor George?

**Respondent**

I know it is the government who trained him and assigned him, here to care for the mentally challenged patients.

**Moderator**

Okay. So we talked about the services we get, medicines and you also spoke of sometimes waiting for like 1 hour long. We also spoke of occupational therapies. Do they get home visits?

**Respondent**

To me truth is one, am a hospital staff, I took a day to come and ask, I’ve been in active service for 42 years, my child became sick before I went on retirement. Not even a day that you will come and check on me. The doctor gave his contact and a nurse also gave hers. I told them I will cater for their transport fee for them to come and have a talk with the child because am being frustrated. Not even a single day has one come to check on us and if you could help us to that effect.

**Respondent**

I've witnessed that before. Doctor has ever come home to check up on my sister with some staffs. We also receive calls occasionally from him checking up on us. Am testifying of what I’ve seen.

**Respondent**

It is not been quite too long since I started coming here. At some point I wasn’t around but my sibling right after me was around so they had time to come around. I also have their contact while they have mine. They called one day and asked of direction and the child also came to stand out to enable them locate the house. So they came and had a conversation with them even in my absence. They had time to teach us certain things and the discussion was a fruitful one for that matter.

**Moderator**

They came home or it was outside?

**Respondent**

They came home

**Respondent**

Please, what someone has done you need to say it. Personally I wasn’t at home when they came. But the doctor called and I told them I wasn’t at home. But truth be told, where the house is located, it is a new site and the kind of hills you would have to climb before getting there isn't an easy thing. But they still went to the house. As at when they came, my sister was around and they came and had a chat with them paying a visit. Once in a while, as I even came here, it is the doctor who called me to come. He keep calling to check up on us. Left unto the follow up he is doing very well in that.

**Moderator**

How regular is it?

**Respondent**

Oohhhhh, if am to check, that they in addition to today makes a 4^th^ time.

**Moderator**

For how many months now?

**Respondent**

Ohhhh, with that, please like a year and a half.

**Moderator**

And errrhhh, aunty …Indistinct chatting followed with a laughter. Okay, with that I understand. Okay so we want to proceed since time is not on pour our side. Errrhhhhh, have you heard of the law concerning mental health?

**Respondent**

For me, I’ve heard of when they come to the hospital and they cater for them and give them drugs among others. Aside that I don’t know of any other thing. If not for this particular case I wouldn't have had any knowledge concerning mental health.

**Moderator**

I want to continue to ask whether there have been instances where they invite you people, the health workers here invites you so that they can hear from you how their services can be improved and benefit your relatives.

**Respondent**

Please, for that, aside yesterday and the previous Saturday that they called me and I told them if I had received the information earlier, like my sister herself would have come because they said there was some meeting to deliberate on some few things which I said my sister herself wasn't around.and he said even if she’s not around, as for me they know me well to the extent that without the card they can get me my folder because I’ve been coming. So the last time he called me that he said I should come since some dignitaries are coming around if not I hadn't received any invitation as such.

**Moderator**

Has any of you attended any meeting of that sort?

**Respondent**

Please, I’ve not been here for that long and this is my first time coming here and with this he called me on the Thursday and said it.

**Moderator**

So that have to do with this meeting, apart from this, have they been calling you for any meeting?

**Respondent**

Yes, they've been calling frequently to ask of our well-being

**Respondents**

Indistinct chat………as we’ve gathered here like this that he’s talking of.

**Respondent**

I’ve not been invited for such aside today. There is no participation. You are given what is available. The nurses don’t ask how things can be improved for us.

**Moderator**

What about the patients, have they ever invited them for a meeting?

**Respondent**

Me my son in particular, this man here, he has been inviting him severally but he wouldn't come.

**Moderator**

What about you who are caregivers, have you ever come together to meet to say you want to go and put before them some suggestions you think it will help improve their services that are being rendered?

**Respondents**

No please. Me personally if not today that we’ve come together like this, like I’ve never come to meet any like this. Eheee, so its today I got to realize this my sibling this is his/her relative.

We never hear of meetings called to plan for mental health service delivery. We are not invited. It is we the SHGs who go to the district assembly to present our challenges. Sometimes we are supported with funds under the district assembly disability fund and other times we are told is not there because central government has not released the Common Fund.

**Moderator**

Okay, thank you. So I want to go forward to the next stage of our discussion. Funding of mental health services. Do you know where funding comes from for the service they are getting here?

**Respondent**

Please, the groups that help, as in the building of the houses or?

**Moderator**

As in cases like those building the houses, DOVVSU, UNICEF or any group you know.

**Respondent**

DOVVSU or what? We’ve only heard of such on a radio broadcast as a group that help them but I know there is something you have to do before they can assist you. That’s what I’ve heard.

**Respondent**

It is the name he wants to know

**Respondent**

Please you said its the name he wants to know? Participants laugh out.

Indistinct chat.

**Moderator**

MIHOSO

**Respondent**

MIHOSO

**Respondent**

MIHOSO, participants laugh.

**Moderator**

So errhhhh, how does health insurance work in mental health provision?

Participant

When I was admitted, I had an insurance errhh, it was free and we were also given food which was also free. Some drugs were also free while others had to be bought. I was there more than a month.

She herself or the son?

**Respondent**

My son but we both were there.

**Participant**

Please oo, we have not been admitted there before, we were admitted right here, what do we call it, municipal here but we paid small. We didn’t get things for free. I don’t know whether its regional that all things are free. With the insurance, I was only the bed we were admitted on that was for free but with the drugs we had to pay for so I will plead you communicate with them because the drugs are unbearable on us. Help us so the drugs can be made free. If it had gone down a bit for us like we will be appreciative. But all the time insurance which you will have to keep in your bag and if you are not fortunate it may get lost. Meanwhile its in the bag for fun, when you get there it doesn't have any impact unless you get to the pharmacy to get the drugs you want. Its very bad too meanwhile we all vote but we aren't covered by the insurance.

**Moderator**

By their view, by your view, do you think there is adequate funding for mental health or its just enough?

**Respondent**

Please, for the funding its very low. If you was to be higher, I don’t think we would've had such challenges as with the drugs and other things. But because its low, they are compelled to manage so they can also provide at least some little kind of service. So please, because you are more heard, kindly speak put for us for the government to come to or aid or the NGO’s to come and support to push this agenda forward.

**Respondent**

For the funding its very low. But what I know is, when you come here, your card will be given you for free but the drugs will have to be purchased by your self. We plead that you help us, we bought the drugs for quite too long and we are almost fed up. And the patients too, they need to get where to keep them so they can stop these smoking habits and other unimportant activities. But because they are roaming about in town, they can easily enter anywhere and get access to them with less stress. When they even decide to carry luggage, at least others will give them something to be taken.

**Respondent**

Please ooo, we not been admitted there before but when we came that we were asked to go and see the doctor and when we went there, we were made to do some labs and other things of which we paid for almost everything. From there we've been coming monthly for the drugs whenever they finish.

**Moderator**

Thank you very much. I will also like to ask, the materials, the stationary and other things, do you know how they plan for them?

**Respondent**

Doctor please what we know is we think maybe the government is responsible for all those things.

**Moderator**

So its the government?

**Participant**

Yes please

**Moderator**

I also want to know, the information they provide here, do you know where they take them to, when you come and you are asked your name, your condition and other details. Do you know where they take them to?

**Respondent**

Please, what I personally know is when you visit the hospital, you cant just go and sit there like that. They need to know what is wrong with you, why you are there and its the doctors responsibility to do so or who else? Even when you get to the doctor he asks you so we know its their responsibility to interrogate you so you can tell what is wrong with you to enable him get you the particular drug needed for condition but with the money issues, I thought its the government that supports with the money issues among other things, that’s what we think. So this is what we know and if there's any other think you can let us know.

**Moderator**

I think I’ve asked this question but let me be sure, is there a process where they give feedback on information given from them?

**Respondent**

Please, as for me, I’ve not been here for long though but I’ve not also seen such.

**Moderator**

Has somebody ever invited you to have this type of discussion?

**Respondent**

The only think is now that you have called us and we are here. So you are the first to do so.

**Moderator**

Okay, So going to our last set of questions, and I thank them for their patience. So all that we have been trying to understand is how mental health service can be widely available for all at our various health facilities. How do you think that can be achieved.

**Respondent**

Please oo, the little idea I have is if there was to be one in our village, we wouldn't come this distance to take drugs here. So if by God’s grace they've come and have realized what is wrong with us and where we stay. Like if it would be possible, then our group that came to help earlier should keep helping and with the little effort that the government will so we those at the remote areas can access it. This condition is such a way that, if there's someone but due to financial challenges, they person will just be roaming about and finally it will be worse. But if there's one nearby, such a person will be cared for and the worst will not happen. So for today, we really appreciate you for getting in touch with us and teaching some things for us to know how to go about it but we also plead that the group that came to support us earlier should also try and reach out to we those in the remote areas and with the support of the government make is available to help alleviate our stress, thank you.

**Moderator**

In what particular way do you expect us to help achieve this?

**Respondent**

Please ooo, its my thought am expressing. If only it will be possible, for us to know there's the need that they make an announcement on TV stations or where they will be situated so at any point in time they can be reached out to. Even if you are not able to go but goes to the regional hospital, that’s okay. That’s my thought too.

**Respondent**

What I also think is, in the cities, bigger hospitals have been built that in any case you are mostly referred there. So if you could support us like you did in the construction of this building with the support also from government. So we plead you to also make available in the Brong Ahafo region such a hospital as big as that. We need to get a hospital as that because going to Accra hasn't been an easy thing at all. My child is mostly referred to there. My child's father is late, how will I manage to get to Accra? So if such a hospital could be built here because going to Accra hasn’t been easy at all. That’s the little I will also have to say.

**Moderator**

So what challenges affect this? Like you want this big hospital, what is likely to prevent this from happening?

**Respondent**

Please, what I think will be the challenge is money. Money is strength. When money is in hands, nothing you intend doing will be impossible. So its money that can hinder these from materializing. An id those the government has trained are adequate enough, then they should send them to the villages for us. I hope that's our greatest challenge.

**Moderator**

And what else?

**Respondent**

I think that's all. If we have these nurses available, they can be sent to the remove areas so in any case we can easily reach out to them. You know the rate of madness is rapidly increasing. Everywhere you pass, there's a mad person there. If they were to be available like the mad people wouldn't be roaming aimlessly. So the nurses should be sent to the remote areas since the mad people are too many in Sunyani here. We know you can do t. ………. Indistinct chatting and laughter……………..

**Moderator**

Any other suggestion or any other thing you want to add or ask that has not been asked or added.

**Respondent**

What I also think is, to my interest they should bring a laboratory here so we can always get there for check when we are referred. And if we could also get a place that, the way the person came, when you give him an injection he wouldn't be able to go home immediately but could take some rest there before going home.

**Respondent**

Building a structure is also important to me much. You need to get one, especially we those who stay with people who are mentally deranged, when they come and are being injected, they can rest there small. When my son was injected some time back, had it not been the intervention of the police personnel, we wouldn't have been able to send him. They had to handcuff him before we could send him. So if there's a room to keep them for like 3 days to see if it subsides then we send them home.

**Moderator**

So if you have any question for me since I’ve been asking all the questions since we started.

**Respondent**

Please, what this my mother said that I want to reiterate it because it is said that mallet split the wood (an Akan proverb). its something that is important to us all not only my mother here. So we plead that as you have had time to come to us because of the love you have for us, kindly also do so for us. Continue to place value on us so you will keep helping us. If all personnel and equipment are available here, it will help fasten things for us and go a long way to improve our general well-being. With all humility this is what we need most.

**Moderator**

Please is anyone having a question?

**Respondent**

Please, mama………….

**Moderator**

Say what you want to say

**Respondent**

All the needed have been said by them. Please, we didn’t know certain things. That’s what I’ve been saying, I’ve not being here for long though but the little time you’ve spent with us has exposed us to a lot. And the little I want to also say is about our home visits, it is something that is very important and very dear to us. If they were visiting the house and advising our brother as our mother said, I think by this time he would've changed for better so the home visit is very dear to us. How we see it, it be more frequent that it used to be. Its also our pleasure that whenever we come here, the manner in which they receive us and attend to us, we really applaud them for that. They are really doing a good job but our emphasis still remains on the home visits, its also part. In addition, our drugs, we are pleading, with all humility so we can have access to all our needs that the duration from time to time that you’ve given us to come for the drugs, even if you have no money you can have it free of charge. That’s the little I also have to say.

**Respondent**

Please, you asked us many questions. You asked what protects mental health patients, I want you to tell us about it. Also, errrhhh, I had two questions but it seems am forgetting of one….hmmmmm…… protecting mad people, and what again?………

**Moderator**

Okay, we have the mental health law that protects the mentally ill persons from abuse from errrhh, dispossession of his material or his possession which maybe his house or money or bank account. He has a right to be treated and and government is responsible in ensuring thayt he is treated. So, He is not supposed to be abused especially even by police. Police can only guide but they are not supposed to arrest or beat him……or beat him down, its against the law. The police cant do that. And even force treatment has to go under a law court. ……………Indistinct chats……….. we are hoping. And because services are not everywhere some of these things are overlooked.

**Respondents**

Laughter within participants…….all the stress and frustrations………..

**Moderator**

So I will like to say thank you so much to them for the patience since morning and for this good conversation, am very grateful.

| **Name of facilitator** | xxxxxxxx |
| --- | --- |
| **Name of note taker** | xxxxxxxx |
| **Type of respondent** | Mental Health Policy Official/ Service Provider (Psychiatrist) |
| **Gender of respondent(s)** | Male |
| **Disability of respondent(s) (physical or psychosocial)** | None |
| **Age of respondent(s)** | The respondent was not asked to give his age |
| **Community Name** | Accra (Ministries) xxxxxxxxxxxxxx Mental Health Authority of Ghana |
| **Interview date** | 24^th^ August 2020 |
| **Start Time** | 12:05pm |
| **End time** | 1: 50pm |
| **Recorder number** | VN870118.MP3 |
| **Name of file (follow convention below)** | Transcript_Key Informant Interview_Mental Health Policy Official. Male.Accra.Ministries.docx |

**Introduction:**

This interview was held in the office of the xxxxxxxxxxxxxxxxxxxxx Ghana Mental Health Authority. This was a Key Informant Interview (KII).

The interview went smoothly except for occasional interruptions xxxxxxxxxxxxxxxxxxxxxxxx.

The interview was a cordial interaction and the responses were frank detailed.

The details of the interview are as below:

**Interviewer**

Please, xxxxxxx thank you for granting me opportunity for this interview and permission to record this interview. As explained, this is a thesis research project that is looking at the integration of mental health care in the community level in Ghana, the role of the health system framework. So I have a number of questions ranging from service provision to human resources, to legislation, to financing, information collection and data management, leadership and governance, and your personal perspective of what integration of mental health services at the community level is.

For the purpose of this interview, please, kindly give me your full name, your position title and where this interview is being held.

**Respondent:**

Thank you very much. You want to test to see whether the sound is ok?

**Interviewer**

Yah, it is working.

**Respondent:**

So, I am xxxxxxxxxxxx. If you want me to go further, [I am] xxxxxxxxxxxxx Mental Health Authority. ‘nice to be part of this study. Thank you.

**Interviewer:**

Ok, could you say where we are having this interview.

**Respondent:**

This is at the office of the Chief Executive of the Mental Health Authority, within the Ministry of Health in the ministries enclave, Accra, Osu.

**Interviewer:**

Thank you sir. Please, sir, what you do, is it your fulltime work or you have some other primary activities [that you do in addition]?

**Respondent:**

This is my fulltime job.

**Interviewer:**

Ok. So, sir I’ll like to understand mental health organisation and coordination in Ghana. What mental health services are available for treating mental health conditions in community based public health facilities in Ghana?

**Respondent:**

So there are two question in one. So, what is the organisation of mental health services and what services are available in the community?

**Interviewer:**

Yes, we will start with what services are available

**Respondent:**

So what services are available, we have curative services. Well, let me start by promotional and preventive services, curative services, [and] palliative [care services]. So, all these services are available to a different degree depending on the location. But these services are available. By promotion, I mean our mental health workers are there in the community, they give a lot of public education to promote people to maintain their mental health [mental illness] while they have it. And the preventive also to let them know the risks, the chance of becoming mentally ill what are the things that can precipitate mental illness or how can they avoid it [them]. So those are the preventive services. Curative, some happens to contract a mental illness, the services are available for that persons to be attended to, at least at that level, until he is requires to be sent to a specialist clinic. So curative services are available and then the palliative, somebody has had the illness in such a way that he probably cannot be brought back to his normal self so how can we ensure that he lives with the condition. So these services are available. Curative range, we have a range of them, we have medications and we have decentralised our mental health work. Well, let me say we have delegated our prescription habits to the psychiatric nurse and the community so that what could have otherwise been prescribed by a specialist could to a large extent be prescribed even by people in the community. So, all those services are available in the community – medication, counselling, yes these services are available.

**Interviewer:**

Thank you sir. So, to the best of your knowledge, can you describe how mental health care services are structured in Ghana

**Respondent:**

The structure of mental health services is very much like Ministry’s [Ministry of Health] own structure of health care delivery. So, the national, the regional, the district, sub-district and the community. So what we’ve done is that we tried to get mental health at all these levels. So, at the national level for mental health we have the psychiatric hospitals which are the tertiary and the specialist care at that level and there are three psychiatric hospitals all located with the southern regions or the southern part of the country [Ghana] – Accra, Ankaful and Pantang. Then for our purpose, we by community all mental health services outside of the psychiatric hospital are community but again we have different levels of them. So, the general hospitals, like to teaching hospitals which are specialised in themselves but with respect to psychiatric hospitals with respect to mental health services once they are outside of the psychiatric hospital we consider them community [health facilities]. So, we have all the regional and big hospitals also having mental health care. Some of them have psychiatric wings of ten to twenty beds where they can admit where necessary. Then you come down, you to the district hospitals. There they don’t have specialised wings for mentally ill patients but they can admit into the wards. We call that virtual beds. They can admit to them for let’s say a week or two and if the care requires much longer time then they will refer to a higher level. Then you go to the community level, the health centres, all health workers are trained to be administer/ dispense first aid mental health care. Some actually have community psychiatric nurses there and then community mental health officers who are not exactly nurses but [*like*] they are technical officers. Then you go to the CHPS [Community Health Planning and Services], again in the CHPS there those who man the places have basic training in mental health care. So, at all these levels we have some care activity or presence there. That is how it is structured.

**Interviewer:**

Thank you sir. So, to you how would you describe CHPS, are they much lower than the sub-district or they are sub-district facilities?

**Respondent:**

CHPS are exactly the community [level health facility]. In fact, they are much lower than district sub-district. In fact, they are the lowest level of [health] care ] facility]. So, you have a district and then, the districts, by the Ministry’s (Ministry of Health] organogram, the districts are the district hospitals of about 60-150 beds [capacity], and the sub-districts are the health centres, plus also general private/ general practitioners. So, the health [centres] they are the sub-districts and then you go lower in the community, that us where the CHPS zones and CHPS centres. CHPS zone for where they don’t any structural facility there but just a zonal division and the CHPS compounds where you have the structure physically located there. Yes, so the CHPS are the lowest of them.

**Interviewer:**

Ok. Thank you very much. These structures that you have described, is this documented for use? Is there a document around what level of services should be provided at each level?

**Respondent:**

Ministry’s policies, yes it is there and we are also within our policy document, yes we have.

**Interviewer:**

Ok. So, how accessible are mental health services across the levels, especially at the community level?

**Respondent:**

We are still making efforts to make it widely available. Now there are the human challenges. Not human challenges in the sense of human resource but the attitudinal. So, if you go to the regions for instance, we have huge mental health presence. If you go a little lower than the regions, then that is where we begin to have the challenges. So, in theory the people are there but you still have some attitudinal issues of people wanting to refer people with mental health issues to others when they themselves should be able to treat. So, it will not be surprising for you to see a [medical] doctor at the district level referring a patient with mental illness to a psychiatric nurse. When the [medical] doctor has basic training, pre-service training, medical training, he has it, and even outside the medical training he’s also been given [training on managing mental conditions]. We have the [WHO] mhGAP, they’ve all been trained, not all but most of them have been trained in that. So, they have that knowledge but the attitude of not wanting to be associated with person with mental illness, in fairness, not just the not wanting to be associated but they [medical doctors] recognise it takes a much longer time to attend to a person with mental illness than a person with physical illness. Somebody comes with malaria, one minute you can dispense the person, somebody comes with mental illness you may need to sit, if you really want to do a good job, may be 20 to 30 minutes and where the [medical] doctors are few at the district level, they may not have that time so they may easily dismiss you or else send you to somebody they think should have all the time. So, at the district level one wouldn’t say that it [mental health services] is widely accessible. Well, it is available but not widely ~~[accessible]~~ and you may not get it the way that you want. But I must also say that another reason is that we have regional mental health coordinators and they are ensuring that within the regions mental health [service] is being provided. We need to go lower and get district mental health coordinators. We don’t have them now even though we have people acting as such they really don’t have what they think a major responsibility to ensure [*that the services are organised?*] so that is a little lacking but we are trying to sort that out so this year or early next year when we have district mental health coordinators they will be able to coordinate and make sure that the [mental health] care is provided the way we want it.

**Interviewer:**

Thank you very much sir. And how affordable are mental health services to the population especially at the lower levels?

**Respondent:**

In theory, it is supposed to be free and that is what the policy and law say. So, in theory it is supposed to be free but being free means, somebody must provide the resources, like medications, like all those things and it is the government that is supposed to provide and since the resources are not there then people will need to find some means to provide the medications and retrieve the cost. So, you realise that though it’s free in theory, in practice they have to pay something. Depending on how much they pay it may not be easily affordable to them. So, these are the issues. So, what I am saying is that yes they are there, it is supposed to be free but it practice they pay something. I must however say that even what they pay cannot recover the full cost. So, if they went outside [the public/state/government] to buy the services it will be much more expensive than what they pay.

**Interviewer:**

Thank you so much. Sir, I want to move to the area of human resources, errh, please tell me about mental health workers in the community health facilities providing mental health care services. I know you talked about specialists, community psychiatric nurses and the rest but what are the mix of mental health professionals providing mental health services at the community level.

**Respondent:**

So, at the community level, and remember by community we mean any all mental health care outside the psychiatric hospitals. So, in the regions, now, errh, in fact we don’t even have; ok the teaching hospitals they have psychiatrists, but generally we don’t have psychiatrists there. So, what we have; we have the psychiatric nurses offering services and they go down and even to the community. So, at the district level, we have psychiatric nurse there, so that’s one. We have clinical psychiatric officers, and these are nurses who have gone for further training to be psychiatric nurses and thereafter they go for further training to be more or less assistant psychiatrists, where there are no psychiatrist they are supposed to offer. Unfortunately, we don’t have a number of them in the districts. We’ve produced about 50 and they are still within the big hospitals, but we are trying to take steps; so, if we are able to succeed, they will be within the regions and the districts. We also have community mental health officers who are technical officers like the foot-soldiers of mental health workers/ mental health nurses. So, they are also there, and we have a good number in the community. And then, as I said, we have the general practitioners of all categories who also have some basics in mental health care [training]. Another cadre of what we call informal community mental health workers are the traditional and faith-based healers. They are there practicing their trade but what we are trying to do is to rope them in as informal frontline community mental health workers. So, as patients go there, they will be able to say that, aah, your condition is not spiritual you go and get the support [*from the mental health facility?*]. Or where they need to give you some basic counselling at their level they can give. So, these are all the structures that we have.

**Interviewer:**

Ok, thank you. I’m quite interested around areas of district to sub-district and any other lower levels. For these categories of mental health service providers you have just described, what are their numbers at the district and lower levels of health care services?

**Respondent:**

The numbers, I may not be able to give you accurate numbers but if you go to, let’s say; as I said, to start with at the very lowest level, we have all health workers who have some basic training and therefore all health workers will necessarily [be able to provide mental health services]; and I think they should not be less than forty to seventy thousand; let’s say about forty thousand nurses for instance and they all have basic training in mental health so, all these are there. Now if you go to psychiatric nurses you will have about two to three thousand, about three thousand psychiatric nurses, a lot of them in the community. And then the clinical psychiatric officers, as I said 50, about 50 them. In fact a cadre of health workers I didn’t mention, two cadres, the clinical psychologists and you have some of them in the regions; clinical psychologists. We have about 20 in the regions; let’s say about 16 in the regions. Then we also have we call ‘psycorps’. ‘Psycorps’ are graduates with basic knowledge in psychology like they majored in psychology at the undergraduate level and when they come out and they doing their national service, they are given some orientation in mental health care and we use them as ‘psycorps’ and we have them scattered in various places. So, all these are cadres that we have there. Community mental health workers or community mental health officers, they should be about, not less than 600 in the community. So these are the cadres.

**Interviewer:**

Thank you sir. Is there a document, if I may say, that says, that describes what mix of staff should be available at each level [of the health care service system]?

**Respondent**

We are now developing the staffing norms. So, it’s not fully developed yet so once we have the staffing [norms completed]. But again, even then we have a document that shows implementation of integration of mental health care into the community. So, we’ve given some very basic figures that if you want to establish a mental health clinic, psychiatric wing in a district hospital, the number of staff that you should have. So, we have some basic figures. Later we can get you [those details].

**Interviewer:**

Ok

**Respondent:**

That’s right.

**Interviewer:**

But if you have all the staff that you need, in a typical community mental health unit, what should be the mix of mental health professionals?

**Respondent:**

Generally speaking, if you take a hospital for every one patient you’ll require about four nurses, every one patient at a time on rotation. So, [in the] morning, you should have one, four psychiatric nurses to a patient; yes that’s right. So for afternoon and for evening. So, that means twelve per one patient per day. So, you multiply that by the number of patients there and that should give you the number, but this is really something that you have to be rich enough to get. But, if you get, let’s say about; at any time if you go to any ward, we have about six nurses on a rotation irrespective of the number of patients there that will be adequate for us.

**Interviewer:**

So for example, will you subscribe to a situation where for every mental health unit at the community level, district and below, at least should have a psychiatrist, a psychiatric nurse and a clinical psychologist.

**Respondent:**

For now, it will be difficult. We really cannot envisage a time that we will get a psychiatrist to every district hospital. So, for now, we are aiming at getting a psychiatrist to every regional hospital and now there are 16 regions so they are going to have 16 regional hospitals so psychiatrist will be there plus clinical psychiatric officers and then as you go further you will have clinical psychiatric officers. Eventually, we are hoping that we can train more. I can easily envisage that in the next three or five years you may get about over 100 psychiatrists at they rate they are getting interested in coming in we can easily produce that, let’s say five years. Then we can get some to go into the districts and eventually, some may even go on their own, private practice and they will be in the communities.

**Interviewer**

Do you know if the WHO [World Health Organisation] has any, errh, should I say, document, directive or suggestion as to what mix of mental health professionals should be any typical community health facility?

**Respondent:**

Not any typical [community mental health facility] but where you have in-patient facility. When I said one to four, that is a WHO recommendation.

**Interviewer:**

Ok, alright. Thanks very much. How about training and accreditation, licensing of mental health professionals, especially those that work in the community [health] facilities. How is that done in Ghana?

**Respondent:**

Three categories. The doctors are accredited by [the] Medical and Dental Council. So, whether you are a psychiatrist or not you are accredited [the Medical and Dental Council]. In the past, Medical and Dental Council only accredited you as a [medical] doctor but now they’ve agreed that yes, your specialisation is also attached so you can get registered as such. So, Medical and Dental Council gives accreditation. Likewise, Nurses, and Midwives Council gives accreditation, and they regulate psychiatric nurses as part of nursing generally. Then other cadres, in fact, physician assistant, clinical psychiatric officers will be also accredited by Medical and Dental Council. Then, like Community Mental Health Officers, as I said, they are supposed to be as technical officers so they are accredited by Allied Health Council. So that is where / these are largely the three; of course, Psychologists are also accredited by Ghana Psychology Council so four accreditation bodies.

**Interviewer:**

And what does their basic training entail?

**Respondent:**

Which of them?

**Interviewer:**

For those categories of mental health professionals?

**Respondent:**

One, to start with, your profession must recognise you as a professional and then, so for instance for a psychiatrist you must have not less than three years training for your membership. And there you go further for two years as fellowship. By the time you are through with your fellowship, you would have spent about six years in training. For psychologist you must have your basic training at the undergraduate level and then go for some two years masters, philosophy, MPhil training plus one year attachment. So, you should have some three years post degree level [training]. [For] Psychiatric nurses, three years post-secondary. Now they are even making it fours [training] equivalent to university level. So, that is that. And then Community Psychiatric Nurses, post-secondary. Also, community mental health officers, three years at least diploma level, post your basic Senior High Secondary school certificate. So these are the categories.

**Interviewer:**

Thank you sir. And, errh, mental health workers providing services at the community level. How are they managed?

**Respondent:**

Well, at the community level you are under the Ghana Health Service. So, once you outside the psychiatric hospital, you are within the agency that is managing you. For instance, if you are in teaching hospital, you are managed by the teaching hospital’s board. If you are in a general hospital, let’s say Tema General Hospital or Fanteakwa Hospital, you are managed by the Ghana Health Service. So, the regional director there is your direct boss or district director as the case may be. So, generally, you are managed by the Ghana Health Service.

**Interviewer:**

Are there any other health workers managed by the Ghana Mental Health Authority?

**Respondent:**

Well, we collaborate with them. But largely, once outside the psychiatric hospital it is not Mental Health Authority that manages you. We collaborate with them. For instance, if we have a psychiatric nurse doing some practice that is not right, the regional coordinator will let us know and we will liaise with the Ghana Health Service. They will eventually have to, if there is any sanction, they will have to offer. So in terms of direct supervision, it is by the Ghana Health Service but we collaborate with them. That is why we say that the Mental Health Authority offers two levels of services or two kinds of services, regulatory and service delivery. Service delivery directly at the psychiatric hospitals and regulatory that is indirectly and all the other agencies outside the psychiatric hospitals by our ensuring that they do the right thing. For instance, we are going to have, eventually, visit committees. The visiting committee will go round to ensure that they, the agencies, are providing quality care, no human rights abuses. If there is any infringement they [the visiting committee] will report to us and we’ll liaise with the Ghana Health Service. So that is the kind of relationship.

**Interviewer**

Thank you very much sir. I know when it comes to the Mental Health Act inside out it’s your baby so how do you see the Mental Health Act promoting community-based mental health services.

**Respondent:**

Mental Health Act promoting community-based mental health services. It does promote. Very good. On paper it does promote. In practice we need to get the structures called for by the Law and that is where I am saying that we need to get the district coordinators in place, which we haven’t got in place yet. So, it leads to a certain gap. We have the regional coordinators they are there and they are functioning very well but the district coordinators need to be put in place. We have the visiting committees [which] need to be put in place too to ensure that they are supervising [and] monitoring quality of services provided and practices. That is not yet in place. We need to get the mental health review tribunal to make sure that people who are admitted to hospital(s) against their will [*are*] we don’t violate their will that is not in place. So, the structures that we don’t have, there are a limitation for us now but, at least, on paper its there and we are hoping systematically to implement them all. Then again, the mental health law calls for a levy which is a sustainable source of financing which we haven’t got yet. So, that also leads to a certain gap because without that we are always at the mercy of other agencies which are providing the services. But if we get it and we are hoping that we will get it, then we be able to provide the funding to ensure that the structures are there, the services are provided at all levels of the [health] care [service].

**Interviewer**

Thank you sir. I should have asked this question. I’ve spoken to a few of the regional mental health coordinators and the district mental health focal persons and there is a mix of responses. While some see themselves as part of Ghana Health Service, there are others who say they are still waiting for the Mental Health Authority to streamline their roles because they are expecting to see a regional, district coordinator straight to Mental Health Authority in terms of the structure of work.

**Respondent**

We need to go on further and give education. I must admit that the education has not gone down very far. We need to get them [to] understand. I keep saying for instance even in school you think people /students know why they are in schools. They are some students if you ask them why they are in school they may not know. All that they know is that I am here to pass my exams and go. Why is he in school? Not everybody knows. In the same way, even people who are employed, not every employee knows why he is there. So, in the same way, people may be there and they may not fully understand. But there’s, as far as the law is concerned it’s clear cut. I must also admit that even the regional directors some of them are a little confused because they think that, some people are thinking that we were creating a parallel structure through and through. Mental Health Authority should have its own structures down at the community level. So, if we have appointed somebody as regional coordinator or district coordinator then he is for the Mental Health Authority. That is not so. We don’t want to create a parallel structure outside of the psychiatric hospitals. So, there it is fully integrated. So, yes, we recruited you as a mental health coordinator but you’ve been recruited for the region. So, you are responsible for the region, you are responsible to the region. So regional director has full control over you. If you are in a district, the district director has full control over you. However, your report should also come to us. And even there we say your report should be signed by the region director so [that] we’re sure that you are giving us the right picture and that, means that you are responsible to regional director. [But] these are educational issues that should go on and we’ve not, admittedly, been able to fully do that but that is the situation. So, if they have a mixed feeling as to where they belong I don’t blame them but it’s clear-cut. In our [Ghana Mental Health Authority] minds there is no confusion. They are recruited by us [Mental Health Authority?] but for the regions and they have allegiance there. And again, as I said, the regional directors, some of them while not having this proper understanding they think they don’t have any control over them [the mental health coordinators] and not prepared to supervise, to give the supervision that they are supposed to give. That is still a wrong conception.

**Interviewer:**

You talked about the mental health policy. What does it entail?

**Respondent:**

The policy is actually detailing what the law says. The law gives a framework of how mental health care is to be administered, the policy takes it a step further. So policy talks about, you are talking about the six building blocks of the WHO, the policy talks about all those things to make sure that they are there. Ensuring human resources, ensuring infrastructure, health technology, ensuring decentralisation, demystifying mental health care, removing the institutionalised conception of mental health care. So, these are the issues that the policy does. And eventually if we are able to implement them, it really lift [up] mental health care [in Ghana]. The policy is also in phases. It is a twelve-year policy phased into three. So, four years, short-term four years, middle-term four years and then long-term. Now this one also takes the policy further into implementation process and therefore every four years we have one-year plan which will take them into one year, one year. This is all largely funding mechanism challenges. We were lucky to have DFID funding some parts, well funding them largely but this year DFID funding does not seem to be able to support the way that we are hoping it will. But even there; at least there’s opportunity for us, while being able to implement them to take mental health care to the quality decentralised lower levels that we want.

**Interviewer:**

Is this policy in place now? Is it active and working?

**Respondent:**

Yes. We are implementing it anyway but it needs to be launched. In fact, it went to cabinet for cabinet approval and cabinet told us that this one does not violate any existing law and therefore it should only have ministerial approval. And the Ministry [of Health] is delaying the approval. So, we’ve told them that we are going [ahead] to launch because they’ve been part of it. So, we are going to launch it within the next two weeks. Then we will publish it [and] it will be online, everything.

**Interviewer:**

Thank you sir. So, I want to look at the issues around financing and budgets for mental health services at the community level. Please, kindly describe the sources of funding for mental health care at the community level.

**Respondent:**

At the community level sources of funding is largely from Ghana Health Service which is not adequate because they do not often prioritise mental health care. So, what they get is very little. And then once a while you may also get some NGOs to support, BasicNeeds and others to support. But we are looking at where you will have government budgetary support allocated to the district, to the community. For instance, if the government makes budgetary support to the Mental Health Authority, even if scanty, and makes budgetary support to the psychiatric hospitals, we are looking at a situation government will make budgetary support to the community level mental healthcare services. It could be given through the Mental Health Authority so we give to the community. That one is not yet forthcoming. So, once a while when we get a little money like, as I said, those days when we had DFID money then we will give some to the community. So, the community mental health funding has been sporadic. It’s not been adequate the way we want it to be.

**Interviewer:**

Please sir, I can always pause if there is an urgent to call to pick up. I can always pause.

**Respondent:**

No problem

**Interviewer:**

Thank you. How is the existing Health Insurance Scheme paying for mental health services provided at the community level.

**Respondent:**

It [health insurance scheme] is not paying and it has a little historical background. Before the [health] insurance scheme came on board, we had the out-of-pocket payment, cash-and-carry. And we managed to argue with the government that our people are not capable of paying out-of-pocket. So, they [the government] managed to get us out of the cash-and-carry system and the government was paying for us. Those days money was not bad, it was quite coming and we were happy. And then the government said no we cannot let people pay out-of-pocket so insurance scheme will be put in place. And so, when the insurance law came in then they were also looking at how not to over burden the insurance scheme. So they looked ta areas where government in already supporting and they say mental health you are already being supported by government so stay out of insurance. We didn’t mind because we were happy because we were getting the support. But with time government support for mental health was not coming and therefore we began to envy the insurance scheme because if people had paid the premium while they were well then at the time they were not well, we could have fallen on them [the scheme contributors] but that has been the situation. So, by Law, [with] the insurance scheme, we are not covered and they explicitly state that we are not covered. But granting the challenges we already have in mental health financing we are trying to see how we get mental health to come under them [the health insurance scheme. It’s been a long walk but we are working on it, making the efforts.

**Interviewer:**

Thank you sir. I know you did mention that some payments are made towards utilising mental health services and these relate mainly to medicines but are they are any other payments that are made for services that you know of?

**Respondent:**

Yes, yes. For instance, if you go there, you will be charged consultation fees the way they are charging everybody. A little lower but, in fact, even the psychiatric hospitals they charge consultation fees, they will charge you for your folders, documentation and they will charge you for admission even though, as I said, all these are all supposed to be free and I have had a number of times to tell Parliamentarians that this is what is happening. The law says free but because you are not giving us the enabling environment, we have had to charge. So, those services are paid for. I must quickly add that what the pay is not full cost recovery. If it were full cost recovery they will not be able to pay that much. But even this little is having adverse effect because not everyone can afford to pay. So, once they know that I go to the hospital I am going to pay admission fees. For instance, if you are going to be admitted you might probably pay not least than [one] thousand Cedis and how many persons with mental illness can afford that. If you are a cardio patient thousand [Ghana] Cedis is no big deal [but for a] mental patient [one] thousand is a lot. So, this is really posing a problem and so it has the tendency to reduce OPD attendance and admissions.

**Interviewer:**

Thank you sir. Errh medical products for mental health service provision. What are the main medical products and devices bought and used and how are they planned for and procured?

**Respondent:**

The medicines. Psychotropics largely [is/are] the main thing. Psychotropics. So, government makes provision for psychotropic procurement and it [the government] adds psychotropics to the general procurement system. That is where we have a little challenge. The general procurement system is every two years. I think the do their procurement every two years or so. Ehm, one. So, there are two challenges there. One is that the procurement cycle by the Ministry [of Health] does not favour us. For mental health products, psychotropics, you virtually need to make your procurement every six months, because it takes, [and] you need to notify [for] unlike other medicines where they don’t need to manufacture for you. You are going to buy Amoxicillin or something like that, it is not only you who are purchasing. Other people are purchasing so they don’t need to manufacture for your specifically. So, they are always producing. So, you can go and order and you get it quickly. So, the two years cycle is for the tendering process. Once you finish with three months, even if you are through within three months money is released, within three months. You can get your medicines. In our case, not many people are using, especially locally, if you are going to get [the psychotropic medicines] locally not many people are procuring [psychotropic medicines] and therefore it needs to be on order. So, you need to order them, give the order then the manufacture for you. And so you need a cycle of not less than [every six months].Every six months you need to start. Once you start it you need to go through the process. By the time one is on the high seas coming, you’ve have started the process again. The Ministry [of Health] have difficulty appreciating this point so we are always behind. So, we have medicines coming and it turns out that may be for the next three to four years we don’t have psychotropics. Indeed until, in the last eight years or so, it’s only three years ago that we had, last nine years, so for seven good years we had no medications [medicines] given to us [by government] and that is a big problem. Fortunately, things seem to be changing a little bit in the last three years. But that is one part of the problem. Our procurement cycle does not seem to favour us. Two, the quantum of money given to us is no where near adequate. For instance, we require not less than twenty-eight million [Ghana] Cedis worth of medicines and you might lucky to get five million [Ghana] Cedis worth. So it is no where near what is adequate, But again, that is part of our predicament.

**Interviewer:**

Is this per annum? The twenty-eight million?

**Respondent:**

Actually, for the two-year cycle. For the two years. Twenty-eight million [Ghana Cedis] for two years. This year for instance, we submitted twenty-eight million and they have just decided to give us five million [Ghana Cedis]. Even the five million might not come. But as I said, in the last three years we are lucky that we can push and get a little. Last year, they budgeted 7.3 million [Ghana Cedis] and we had five million [Ghana Cedis] worth. So actually, it was 5.9 million [Ghana Cedis] worth so it was 1.4 million that we didn’t get. In fact, in a very long time that has been our highest orders that we’ve got. This year, instead of 7.3, they gave five million. And this money is actually is being given through the national health insurance though we don’t benefit from them directly they can make some budgetary allocation for our medication procurement. So, those are the two things but eventually if we are able to get our mental health levy and we have all the money we can do our own procurement without having to go the Ministry’s procurement. So the problem of the cycle, we will be able to offset that plus the quantum of money. So, until then these are the challenges we have.

**Interviewer:**

You talked about government does all the procurement then you also mentioned the Ministry, I guess you mean Ministry of Health.

**Respondent:**

Sorry.

**Interviewer:**

You talked about government does all the procurement. I guess you mean Ministry of Health.

**Respondent:**

Yes, Ministry of Health.

**Interviewer:**

Ok, I just wanted to be sure

**Respondent:**

And they are supposed to involved us anyway but often before you realise they say this is what we have done, bring a little input. We are really supposed to sit down on the same table and do the procurement.

**Interviewer:**

Are there other medical products that are required for providing mental health services?

**Respondent:**

Routinely, it is the medication but on and off we have other things. Like, for instance, ECT machines. ECT machines for a long time [the] government was not [providing]. I think the last time government bought [ECT machines] for us was about two years ago. These are broken down and last year we had a religious organisation procuring three for us and we are even having challenges getting all three functioning. So, once a while we get some [assistance]. DFID for instance also procured laboratory equipment, x-ray equipment [and] laundry machines, so they managed to support us with some equipment. So, once a while we have some such support but otherwise government is support to be responsible and theirs is largely psychotropic medicines.

**Interviewer:**

And how are all these medicines, ECT machines and the rest distributed down to the community level?

**Respondent:**

So far, as I said, with the community, the Ghana Health Service is responsible. For instance, the equipment. Now the psychotropic medicines they all get including the Ghana Health Service. They all come to the central medical stores, in fact we are responsible for the [psychotropic] drugs that come. So when the drugs come and they are at the central medical stores, we distribute not only to Ghana Health Service, in fact even the teaching hospitals, the quasi organisations, the Prisons, Police they all come [for the medicines]. That is why when you are ordering we want to make sure that we order not only for the psychiatric hospitals but we order for them all. So, that one it goes down there but the challenge as I said is often that we don’t have it [the psychotropic medicines] and therefore it does not go. So if you go the communities the nurses find a means of buying and then sell to the patients.

**Interviewer:**

So, yes, yes, you also did talk about the procurement process [and] how they are planned. The 28 million budget for mental health services, is this usually based on data generated right from the community level?

**Respondent:**

Data generated. Yes and No. Yes, in the sense that over the years we’ve known the usage. So, based on how much they use that informs us how we give it. As I said, not just the public sector but even the quasi-government. So, based on their usage [and] their requisitions within the year that informs us how to make it. But as I said, largely, if you don’t get based on what you need and you get based on the ceiling and you cannot provide according to their needs, you will provide as you have it.

**Interviewer:**

I am also curious. Do you think there is an over-use of medicines?

**Respondent:**

In some sense yes. And what I mean by in some sense yes is that until recently and that was one of the things that the policy is trying to change. Until recently, mental health care was overly medicalised. In other words, it was like if talk about mental health, we are talking about the psychiatrist or the doctor and the nurse and then medicine. As if every illness requires medicine, which is not so and that was because we didn’t have the other compliments of mental health workers. In fact, until three years ago, we didn’t have a single occupational therapist. Ministry of Health did not even recognise the clinical psychologist, so they were not recruiting them. So everything was just the doctor, the doctor, and if you came from outside that I am a clinical psychologist, I’m a family therapist, bla, bla, psychotherapist I want to offer my services, the Ministry didn’t have any category for you. You didn’t fit. So, all that we knew or the government knew or the Ministry knew was that simple category doctor or psychiatrist, psychiatric nurses, medicine. So, by default not by design, by default we were overly medicalising and therefore using medicines where probably some simple counselling could have done. In that case, you over-using medicines. Then I must also say if you go to the districts where not only are we, yes ok, apart from the fact that we might be using medicines, not only were people using medicines they are not supposed to use but even the type of medicine that they were using. I have been to a community in the remote area where a community mental health officer who is not supposed to prescribe was prescribing clozapine which is a highly specialised drug. So obviously this is [mis use?]; and that was because there was no medication and that was what is available so you just give. And clozapine is dangerous that is why it is a highly specialised drug to be used by specialists. So, yes, there is that tendency of over-using medicines. But again, even those who require it [the medicines?] where the specialist is supposed to provide, the medicine will also not be there so in some sense where medicines are supposed to be provided, they are also not there. So, these two issues are there. And we can only get the right balance, if we have the right mix of staff at the various places where the should be and the right mix of staff I am not just talking about psychiatrists but the psychologists, occupational therapists, counsellors and what nots, all there and providing what they should be providing. And then the right instruments and medicines and logistics there then you will able to ensure that people are provided what they should be provided.

**Interviewer**

Thank you so much sir. So, I want to look at information flow and feedback chains across the mental health service levels. What is the level of information flow, from community to district to national [regional[ and regional [national] and vice versa?

**Respondent**

Again, there is a beautiful system within the Ministry [of Health], information flow system. So, at the community level you provide to your sub-district and the sub-district provides to the district director and the district director provides to the regional director and the regional director provides to the Ghana Health Service.

*Note: There was a pause to allow an official of the Mental Health Authority hold a brief discussion with Prof. Akwasi Osei, the Chief Executive Officer of the Mental Health Authority of Ghana.*

Now our district focal persons are supposed to give the information not only to the district director [of health services], horizontally, but also vertically to the regional mental health coordinator and the regional mental health coordinator is supposed to give information, horizontally to the regional director [of health services] and also to the DCC, Mental Health Authority headquarters level and then we are also supposed to collate that and pass on the information to the Ministry [of Health]. But apart from that there is the DHIMS, [District Health Information Management System], so even wherever they are they can code it directly into that. I understand there a few challenges. If you code it, it is difficult even for you to get access back [into the system] but everything is working well, you are supposed to code it and get information to see what is happening. You can’t see somebody else’s information. You are supposed to see what you yourself keyed in. So, there is that kind of information system which we are trying to improve upon it but that is there. What else we are trying to do is that we are trying to work out a register which we will widely distribute to the localities because it is the register [that] they will use to fill and based on that they will put in into the DHIMS and then you can tap it.

**Interviewer:**

How about the feedback? How is feedback provided? Performance? Issues of challenges that may be there or reporting emergency? What is the feedback process?

**Respondent:**

Again, they are supposed to provide information to the district directors, district focal persons who are supposed to give the feedback to them. But again, the district focal person don’t have allegiance to them [regional mental health coordinators] because we did not appoint them. That is why we are trying to get [them appointed] because the district focal persons are like acting district focal persons so if we get our own district coordinators the feedback will be better but now at the regional level they get all that information [from] the regional coordinators and the regional coordinators give us any support [that] we can give them. We’ve even been using telemedicine and giving them the support but all that information is supposed to be collated by the regional coordinators and they will give the feedback to them [and] also tell us and if there is any feedback we also give. So, that system is supposed to be there. How effectively it is implemented is another thing, I must say but that system is supposed to be there.

**Interviewer:**

Performance appraisals. Are they working and [*interrupted*]?

**Respondent:**

Performance appraisals. Again, at the agencies where they are, they are supposed to do that. But there is a general problem with performance appraisals in the country where. People only go for appraisal only when they are going for promotion interviews. Sometimes, two, three years they’ve not done appraisals but now that they are going for interview you need to reconstruct from memory so really, it’s not been very effective. But we are looking at how we can make it effective. In fact, at headquarters level how we can make appraisals effective. On a daily basis we meet with you we decide on what you are supposed to do then we give that appraisal based on that. For now, it’s really not that effective. It’s more like a needs-based appraisal.

**Interviewer:**

So, errh, in terms of service provision performance is there a process of trying to collate views, feedback from service users, caregivers, from [the] general public about, I wouldn’t say service quality but about service satisfaction and what else can be done to improve especially at the community level?

**Respondent:**

We are looking at, in fact our indicators in designing service. As part from [the WHO] QualityRights. In fact, quality services, they are supposed to have surveys where they do. The psychiatric hospitals they do, but not [regularly] may be once a year. But they are supposed to have surveys and these surveys should go down. So where you can get them, at that point, their satisfaction levels, their challenges and you solve them. The challenge with these surveys is that if you identify problems to what extent are you solving [them]. So, you are not solving [the problems]. Next] so, So, text time we come to you for a survey, they are even not interested. These are real structural issues we have. So, you come to me to ask me, indeed right now there is a work we are doing with Accra Psychiatric Hospital, the psychiatric hospitals, Humphrey is leading that aspect and they are saying after all you come to get this information, of what benefit is it to me. And so, that makes them reluctant. But, otherwise, the system is supposed to be there but implementation becomes a challenge.

**Interviewer:**

Yes, so have you been privy to any information voluntarily provided or unsolicited from say service users or caregivers or just interested stakeholders and how it has been used to improve services?

**Respondent:**

We get through the NGOs. NGOs, BasicNeeds, MEHSOG, MindFreedom, so we get feedback from them and we see how we can improve upon them. But again, as I said, a lot of this feedback are structural issues, which until we are able to get hands on our funding mechanisms, it is difficult. If you are not able to provide funding for anybody it is difficult to tell him [or her] to do this or that. But we get feedback from them. What we are also doing is we’ve built a research agenda is to be able to embark on [a] survey. Survey, not only household survey to see what is going on but we also our own staff survey, national survey, so that we will get directly we will get directly the picture of what is happening. That is not yet taken place.

**Interviewer:**

Ok, thank you so much sir. I want to look at the issue of surveillance and research, which have just touched on. What are ongoing research going on around or ongoing research on community mental health services or mental health services provision at the community level?

**Respondent:**

That is an area that we’ve been very weak as a system and we are trying to source, we are trying to populate our research department and when that is populated, we will be able to embark on that. So, now it’s quite sporadic or some other body comes and says that let’s collaborate and do some work then we do. I just mentioned the case of [the WHO] QualityRights that we are trying to do some survey at Accra, Ankaful and Patang. So, that is also the area we are collaborating and you realise that all these ones are not generated from us but others because they have the source of funding. But if we have our own funding then we decide. We have the research agenda then we go according to that. So, for now, I will say our research has been very weak. Or once a while somebody wants to conduct more clinically-based research that’s also done but what you get cannot translate to nationwide. So, it is an area that we’ve been very weak. But we are trying to, otherwise what we are trying to do is, as I said, household survey, national survey of, epidemiological survey of conditions that we have [in Ghana]. Right now if you ask me what is the prevalence of schizophrenia, what I will quote to you is the textbook one. One percent, it may not necessarily translate to what is in Ghana. If you ask me for depression, [I will tell you] either textbook one or clinical based, but we need to go to town and do that survey. We are liaising to see how that can be done. Errh, that’s one possibility and then, in fact, we say we are doing a lot on public education against stigma, to what extent are succeeding, what’s the baseline, how well have you improved? We need some survey to be able to look [at] that to say public perception on this condition has changed. We need to do that. We need to do [research on] people’s attitudes, behaviour [and] PKA [**P**erception, **K**nowledge and **A**ttitude], perception, behaviour attitude. We need to do all these. So these are all [possibilities for the], as I said the research agenda. Traditional medicine, what is their own uptake on our education? To what extent are they able to and prepared to compliment or comply with what we want to do? These have been outlined but to take them on board we need, one, to populate our office, and then to get the funding for them. We are hoping that will be done.

**Interviewer:**

Thank you so much. So, I will go into my last set of issues. Issues of leadership and governance. What is the leadership of the mental health system like in Ghana?

**Respondent:**

Exactly in what sense? What is it like?

**Interviewer:**

Yes?

**Respondent:**

Exactly, what do you mean?

**Interviewer:**

Like, how is it structured? You’ve talked about it following a certain laid down levels

**Respondent:**

Ok. So, if you come to the Ministry [of Health], the Ministry [of Health] used to be until 1996, Ministry of Health used to be the policy maker and the policy implementer. And we realised that it was like the referee on the football pitch being the player at the same time. So, it didn’t help much, and so you could not.

***Note:***  *there was a pause for xxxxxxxxxxxxxxxxxx to receive a telephone call*

So, we had the Ministry [of Health] deciding now to be responsible for policy-making and supervision and then cede implementation to agencies. So, [at] that we had Ghana Health Service and Teaching Hospitals as the first two agencies within the Ministry of Health to take up the policy implementation. And since then, we now have about twenty-five agencies, including the original two, and Mental Health Authority is one of the agencies. So, Mental Health Authority has the mandate of the developing or proposing policies to the Ministry [of Health] and Ministry [of Health] agreeing and giving it back to us to implement. Policy on how to ensure mental health care is given everywhere [in Ghana], quality-wise, that’s right., that’s right. So, that has been the mandate. So, we have the Mental Health Authority, and at the highest level of the Authority, we have the Board, the Governing Board of the Authority which advises the Authority how to implement the policies the Ministry [of Health] would have given to us. so, below the Board, we have the CEO [Chief Executive Officer] who is also part of the Board. The CEO is directly responsible for the implementation of the policies. Then under the CEO, we have Communication which will ensure we communicate what we want to do and we have some six directorates. PPME (Policy Planning Monitoring and Evaluation), Technical Division, we have Finance, we have Audit, we have Collaboration, we have Quality Control and QualityRights and I think one more.

**Interviewer:**

Community?

**Respondent:**

Community level mental health care comes under Technical.

**Interviewer:**

Technical. Ok

**Respondent:**

So, under Technical, we have the hospitals responsible for institutional care and community. So, all the these are [there]. The Community, go below, we have the regional community / regional mental health coordinator, committee and coordinating team. You go below, district. So this is the organogram there [*pointing to it pasted on the part of the wall of the office above the chair of the desk of the CEO*] that shows exactly how it [the Mental Health Authority] is supposed to be structured. But as I said, outside the psychiatric hospitals, within the regions, the agencies responsible there take care of them [mental health services] but we collaborate with them so that we ensure [service delivery] and that is our regulatory mechanism to make sure that quality care is being given to them. So, that’s the structure existing.

**Interviewer:**

So, that’s the structure. How about the quality of leadership. What is your impression of the quality of leadership across board?

**Respondent:**

Across board, what is left, in fact, the regional coordinators for instance grumble because we don’t give them the resources. If you are not giving them the resources then, in fact they say to whom much is given from him much is expected and if you turn it around from whom much is expected to him much must have been given. So if you don’t give much then you really can’t expect much. And for two years we’ve not given the regional mental health coordinators anything. We are hoping that regional directors [health services] will provide them something. But the regional directors [of health services] they also operate on programmes. So a programme comes with money and they support you but if mental health care is not coming with any money then they are not getting. If we had at the Authority level, we had money then we would be able to give them. Three years ago when we had DFID supporting us, we gave them [the regional coordinators] up to a total of about [one] hundred thousand [Ghana Cedis] then they were able to work and give us results. So that has been the [result?]. so quality is not so much the persons, they are all very good but do they have the resources to operate? And since they don’t have the resources it gives the impression that they are not doing very well but even there, the little they are doing, they are doing very well, I must say. So that has been basically our issue. Mental health remains an afterthought and is excluded so leadership and governance is remote, not there. It needs to be consciously crated and directors accountable.

**Interviewer**

Ok, Errh I was speaking with another national level person and the position was part of leadership is level of interest and commitment and that if community mental health services must work, the leaders at the helm of affairs, especially at the lower levels need to really show commitment to mental health beyond funding. What is your position [on this]?

**Respondent:**

It’s like marriage. Is it love or money? You need both. So the musician will tell you *Ɔdo kakra sika kakra* so they tell you that they can’t eat love. So, you need to give money to support. So yes, the passion is there the commitment is there and I always tell them, behind them, in front of them that they are doing a lot. Given what they are not being given, if the passion was not there, they would have stopped totally. For instance, let me tell you, if you go to psychiatric hospitals and I know our focus is on the district but just to give you an example, yes focus on the community. The psychiatric nurses, all psychiatric nurses, and including those at the community level are supposed to be given uniforms, two uniforms a year. I don’t remember the last time we gave them. Over eight years. If they didn’t have any passion, you have not given me the uniform, basic thing for me to come to work, so, that will even be a reason why not to come to work but because of the passion they are doing the little [that] they are doing. So the passion, I think it is there but that passion needs to be supplemented with the logistics and the logistics, once it is not flowing, it makes you not see the real passion. So, I think that’s the point. I think that for the passion they have it. If at our performance review the little they are doing with the little they have you’ll realise they are doing a lot.

**Interviewer:**

Thank you sir.

**Respondent:**

Welcome.

**Interviewer:**

So, I now want to get your personal perspective and of course technical perspective of what integration of mental health services at the community level really mean

**Respondent:**

Ok, I will tell you my [perspective] what lack of integration is and then we go there. So for the lack of integration as used to be the situation before we tried to put in place measure to integrate. Even at the highest level, doctors for instance will see a patient behaving strangely [and] without even touching the patient they will say abnormal behaviour, diagnosis psychosis, refer [to the] psychiatrist or psychiatric hospital. They won’t even touch. When these patients come and you realise that for all you care it was meningitis. If they [the doctors] had touched, simple touch, they would have seen that this person has high temperature. Now the COVID-19 is making things worse but even those days when COVID-19 was not there, if they were to touch you, the would have realised that you have high temperature, therefore [it] couldn’t have been simply psychosis. There is something organic behind it. That was the situation where they were seeing themselves as separate from you. Now with the integration, ok that was the point. So, you go to all levels, they see mental health as something strange, a different animal so they won’t touch. But now, they are beginning to appreciate differently. So, with integration, two things, one, people should see mental health as part of health. So, wherever and whichever health worker as long as you are seeing anybody, recognise that person may also have mental illness so treat him in totality so holistic care. So, that is very important. Two, don’t think somebody sitting somewhere should see him [or her] when you have not done your part. Look at your part and see [and] even if you realise that it is [a] mental health [condition] you have some basic mental health training, at least, first aid mental health training. What can you do basically before you refer. So, if you can get them appreciate that we are gradually integrating mental health care into general health care. So that is one level. Two [three?], now we also want them, every health facility, to have, not only to see malaria and all those other things but also to see mental illness as part of your routine treatment and where possible to have a unit. So, if you have, at the district level, you have a unit that see diabetes, you have a unit that sees hypertension, you have a unit that see whatever, you should be possible for you to have a unit that sees mental illness. At the district where you have about 60 to 100 beds you don’t expect you to create a wing for persons with mental illness but integrate them into your ward. So, virtual beds. So where there is, let’s say you have allocated 5 beds and there is no mentally ill patient to occupy that you can take it to [for] any other patient. But as long as you have not gone to your level of 5 [beds] a person with mental illness cannot come and you say there is no bed just because you don’t want to take him. So, this is something we want them to understand. So, if they have that we are integrating.

Then, if you go to the bigger hospitals, as you have a wing for the department for maternity, department for paediatrics so also get a department for mental heal health. So, a psychiatric wing of 10-20 beds. And we have, at least, three regions which have that and now the government talked about regional hospitals that are being built and we managed to get them to appreciate that they are going to incorporate that. So, if we get that then we are integrating mental health care. So, by integration we want to make sure that mental health is not seen as different animal but part of our health care system. If you need specialised care just the way you require specialised care for any other physical condition, fine, then you require that but don’t take it as some different thing which should not be mixed. They should be provided together.

A third level of integration is that even the psychiatric hospitals should also see general cases and Pantang has started that very well. So Pantang, they have a maternity unit, they have an eye unit, they are having all kinds of things [health care services]. So you don’t go there and everybody thinks that once you are seen there then you are somebody with mental illness. You could be there [and] it could be [for] maternity, it could be eye or anything. So, these are the three areas of integration that we are looking at.

**Interviewer:**

Thank you. So what opportunities exist to make this work better? This integration that we are talking about, having wings alongside all [other] units, ensuring that general health workers see, even if it is a first line [treatment] and the generally predominantly psychiatric facilities also see general [physical] health conditions, what opportunities exist to make this work well.

**Respondent:**

One opportunity is that for a long time we were having challenges getting an MoU with Ghana Health Service. Now we have an MoU. It’s not fully signed. We need to get the Chief Director to sign. So, once we have that signed then we will sit down with them and the areas that we need to get to and get these issues done we will sit down and ironed them out, tease them out. Once Ghana Health Service at the highest level have understood and they have sent out information top the regions and districts, then that will be it. So, that is one thing, that’s an opportunity. We’ve managed to get it signed. In the same way, we have a similar MoU signed with the other agencies, the teaching hospitals. Then they will understand that this is how we understand integration. The concept of integration that I have told you will be part of the MoU with them. Then they will be able to incorporate them. So, that’s it. And then plus of course the education. We need to embark on education. You’ve given the MoU [that’s] paperwork but you need to educate the people on the ground, the nurses, the doctors so that they will get this understanding. For instance, epilepsy, we started epilepsy training because epilepsy was being seen by us and epilepsy is not really not a psychiatric disorder. It is a neurological disorder. But when we started giving them training on epilepsy during the Fight Against Epilepsy then they began to treat patients and not having to refer them [to psychiatrists]. But we were nit able to sustain that programme so they are going back they are still referring epilepsy patients to psychiatric units. But this requires constant training. Once you start training people, it looks like [and] I keep saying it, it’s like once you start gym you need to continue to reduce your weight. You will need to continue. If you stop it you put on weight again. We need to continue that training and this’ all funding mechanism.

**Interviewer:**

Thank you sir. What can you say about private sector participation in mental healthcare service delivery in Ghana. There are also a number of psychiatrists and churches with rehabilitation centres that I have become aware of in mental health care at the community level?

**Respondent:**

Welcome

**Interviewer:**

Describe private sector participation in the delivery of mental healthcare at the community level

**Respondent:**

Generally, there’s not involvement of private sector in the mental healthcare service provision. The services provided by traditional healers makes me say that there is private sector participation in mental healthcare service delivery in Ghana. There are also a number of psychiatrists NGOs and churches with rehabilitation centres that I have become aware of. That is what can be said about participation of private sector. It’s really not there or structure as with general health care services.

We can include traditional herbalists and faith-based healers as part of private sector so there is some involvement. However, if it is involvement of private orthodox / formal or western medical services, there is low involvement in mental healthcare service delivery at the local level. The few that are there are in the urban areas, and this includes the religious bodies that ran rehabilitation centres.

**Interviewer**

How about general community level participation and ownership of mental healthcare at that community level, how is that?

**Respondent**

**Interviewer**

How do you see the growing international attention to mental health? Has it got any opportunity for Ghana?

**Respondent**

Yes. It has lots of opportunity. Lots of opportunity in collaboration, in fact research collaboration [and] that’s very, very good. Lots of opportunity for us to interact to see where they can even support us [in] training. So, lots of opportunity and may happen. What we have not been able to take advantage is even funding mechanism. So, with international interest there is also funding mechanism, but we have not been able to tap it. That is an area that we are also fallen short. So, as we are able to beef up our own proposal writing skills and all those things, we will be able to tap them. But it’s good that we have, not only international attention on mental health but also on Ghana. That is something very good and we need to take advantage.

**Interviewer**

Well, if we have opportunities, we will surely have challenges. What are the potential challenges that could go against this effective integration of mental health at the lower levels?

**Respondent**

The challenges are still the fact that it is not everybody who understands and you need to do a lot of education. People still see you as errh with some cynicism. Even the concept that, when I say that patients at the district level, they don’t need a unit. They don’t need a ward, but we can treat them at the general wards. People are even resisting that [saying] how can bring somebody aggressive but we’ve all been trained. And you can get somebody from operation, from theatre, who is also aggressive, what about that? You can get a woman from after labour can also be aggressive. So, in the same way you can also get some with mental illness [being] aggressive on the ward. But that requires further training further education. So, resistance of some sort by persons, both the implementers, the workers and also the policy makers at the community level. So, we need to work a lot on them. And two, integration also means providing resources for them to take care of your patients there. So, if you are not providing resources then they think that their limited resources are for patients who are dying and your patients are not dying then they will not give them that attention. So, resources, we need to do that and this requires constant education [and] providing the resources. These are obvious challenges.

**Interviewer:**

This reminds me about the saying that mental health does not generate anything why should we put our money.

**Respondent:**

Precisely.

**Interviewer:**

How can that be gone around?

**Respondent:**

Once we get them to understand. For instance, it’s like when the WHO came out with the Millennium Development Goals (MDGs), mental health was not mentioned. But as we got people to understand that yes, you are talking about reducing infant mortality but you did not talk about maternal mental health so until the mother’s mental health is well, mortality will still be high, infant mortality [will still be high]. So, if we get them to understand that they will realise they will need to spend part of their resources to attend to the mental health component of the mothers. So in the same way as we give the public education eventually they will appreciate that you need to be mentally well to be totally well. Even health generally, you know in the past, at the bank we are seeing money, you we are not seeing money from you so [you are not our priority] but now they understand that health is wealth. And I think I will give credit to [Courage] Quashigah. It was Quashigah time that we got people to understand that health is wealth. So if you want to see wealth being created ensure that health [is prioritised]. In the same way, we need to go a step further and say that if you want to health creating wealth then make sure that mental health is taken care of so that you can have the totality of health to create wealth. So, it requires constant engagement and education.

**Interviewer:**

Thank you so much sir. I think I have asked my questions but if there is anywhere that I have not touched on that you think you want to add on to or a recommendation that you have not already talked about, I will be very grateful to hear.

**Respondent:**

May be the only thing to say is that we talked about the mental health law and you’ve talked about how has the law being implemented? May be a question implied is what would have happened without the law and what is the role of the law?

**Interviewer:**

Actually, it was asking how the law is promoting community mental health service provision.

**Respondent:**

That’s right. That’s right. So, without the law, the status quo would have been as the past. Things will have been disintegrated

**Interviewer:**

Yes, doing it the way you want or some thing like that.

**Respondent:**

May be. The other thing is that are we hoping the law will be revised. In fact, the WHO requires that a mental health law is revised about every ten years, latest twenty years.

**Interviewer:**

And now to be compliant of the UNCRPD.

**Respondent:**

With the CRPD. Our Law is already eight years. And I have been telling my people that, in fact, it was passed in 2012 so by 2032, we should revise it. So twenty years. Now knowing how long it takes a law to be revised, it takes about seven years. If we are going to get a new law in 2032 and you take seven years out of it [by] 2025 we must begin to work on it. So, five years from now we must begin to work on the new law. And our board has said we should document the challenges we are currently facing with that law. One that if we are going to revise the law, [and] I think we should do is, and if by that time we are not yet ready to do it then the law has really not worked. If at the time that Ghana Health Services was being created by the Ministry of Health if we had been errhh proactive enough to get them to establish a mental health directorate there wouldn’t have been need for the mental health authority but you then create a mental health directorate [in the Ghana Health Service]. Mental health was not taken care of. Then later when they realise the need to care of mental health, they were looking at where to place menta health so the placed menta health under ICD (Institutional Care Division). And if you go to some of the regions, some of the regional directors rightly agree that ICD is not the right place, so they put it under public health department. But all these two, both issues have challenges. If you put mental health under public health, you are saying that mental health is all public health issue, but you need some curative measure/ clinical measure and that cannot be taken care of. If you put mental health under clinical care division [then] you are saying mental health is all clinical issue, institutionalised issue, which is not. What about the public [health] component? So, you need a directorate that can combine both. So, if we have had mental health directorate, to take of care of [the] totality of mental health issues, the [Mental Health] Authority would not have been needed. So, at the time of the Mental Health Authority, we would not have been needed a mental health commissioner to serve only the regulatory mechanism and not service delivery. The teaching hospitals would have been under the Ghana Health Service alright. Now that we missed that opportunity and we have had to create a Mental Health Authority, I am hoping that we can get the uptake fully so that by the time we are revising we can then say psychiatric hospitals now go and be taken care of by the Ghana Health Service for now the understand, they can take care of you. So, that they [mental Health] Authority can now turn into a Commission and only regulate. So, our next law, and I must say not many people understand this concept. When I say so, they you people want your own authority, why are you now making this proposal? They don’t understand. But if by five years we want to revise the law and we have really been able to implement the law properly, that is where it should go. So, that is the relation. In fact, Nigeria they are writing a law [and] they are hoping to pass their law and that’s the way they are looking at it – a Mental Health Commission and not an Authority.

As for Cape Verde, there is a seamless mental health care so they don’t even have a psychiatric hospital. What I am not too sure is that to what extent are they really taking proper care of the persons with mental illness. I am not too sure, but they claim that’s [what they are doing]. So, that’s the direction that eventually you should move to. So, if we pass a new law, let’s say mental health Act 2032 and it is still as it is, with an authority, it means that we have not been able to fully integrate. If we can fully integrate, you asked about what kind of integration, if we fully integrate, and you have asked about what kind of integration, if we can fully integrate, we should not have anybody overseeing mental health. It should be the same system overseeing it. That’s right.

**Interviewer:**

Thank you so much. And in case you have any questions for me myself regarding the study?

**Respondent:**

So, for me [the] question is that to what extent is your study going to benefit mental health in the country?

**Interviewer:**

Well, errh, the idea is that once it is written up, may be with a few publications, it will get to the attention of the power that be but I am also intending to do a dissemination of whatever I have gathered, hoping that it will reach the attention of government. Similarly, working with my supervisors, I guess if there are any fora, say the health summit or whatever, where we could work together to request to make some presentations to the Mental Health Authority or the Ghana Health Service or the Ministry [of Health] directly and we have such opportunities, we will do that. So, we’ll continuously use the information in peer review journals and the thesis report itself. And you know from my background, my other side is (being) in working in mental health with BasicNeeds[-Ghana], we will also try to make use of whatever findings in our advocacy.

**Respondent:**

Good. Thank you.

**Interviewer:**

Thank you. I am most grateful

| **Name of Interviewer/ facilitator** | xxxxxxxx |
| --- | --- |
| **Name of note taker** | xxxxxxxx |
| **Type of participant** | Mental Health Service User |
| **Gender of participant** | Female |
| **Disability of respondent(s) (physical or psychosocial)** | Not indicated |
| **Age of participant** | 40years |
| **Community Name** | Not indicated |
| **Interview date** | Not indicated |
| **Duration of interview** | 40min. 56sec |
| **Recorder number** | VN870111.MP3 |
| **Name of file (follow convention below)** | Transcript individual interview_Mental Health Service User.docx |

**Introduction**

This individual interview was held with a person with mental health condition from a community (name of community not indicated) within the xxxxxxxx of the xxxxx. The participant is a member of a Self-Help Group (SHG) known as the xxxxx.

The participant was selected for this interview because she was receiving regular treatment of the mental illness from the mental health units of the xxxxxx hospital.

The interview was in Twi, the local language of the area and widely spoken language. A digital voice recorder was used to record the discussion and which were complemented by handwritten notes. The details of the discussion and written up below

**Interviewer:**

So, tell her that I thank her for agreeing we should do the recording (to interpreter). The discussion is about research. We are doing our investigation on mental health services to understand how the services are provided and how they can be improved. So, there is no right or wrong answer to this discussion. She has to just tell us what she knows and feels. The other thing we want her to know is that we are not going to be mentioning her name and say she said this and that. We are going to give a code. We are not going to be mentioning her name. So, if she agrees that we can proceed we will proceed. We further want you to tell us about yourself; that's your name, your father's name or surname, your age, the condition you're living with and for how long have you been living with that condition.

**Participant:**

Okay, my name is xxxxxxxxxxxxxxxxxx.

**Moderator:**

How old are you?

**Participant:**

I am 40 years of age

**Moderator:**

Who do you stay with?

**Participan**t:

I stay with my mother, xxxxxxxxxxxxxxxxxx.

**Moderator:**

What did they say is wrong with you when you went to the hospital?

**Participant:**

Actually, they didn't tell me that something was wrong with me.

**Moderator:**

Can you describe what made you to go and see the doctor?

**Participant:**

Initially, there was a problem and I think it was spiritual.

**Moderator:**

Okay, so how was it affecting you?

**Participant:**

Actually, I was hearing voices but now it has stopped.

**Moderator:**

For how long have you been in that condition?

**Participant:**

For 7 years now.

**Moderator:**

For how long since it stopped?

**Participant:**

For 1 year now.

**Moderator:**

Okay, thank you. So when you go to the hospital, what kind of services do they give you?

**Participant:**

They usually give me some injections.

**Interviewer:**

What process did they take you through before giving you the injections?

**Respondent:**

They didn't take me through any process.

**Interviewer:**

Did they take your temperature?

**Respondent:**

No, they didn't.

**Interviewer:**

Are there people with the same condition and where are they getting treatment?

**Respondent:**

Absolutely not, because the condition is spiritual.

**Interviewer**:

Were you able to afford for your treatment?

**Respondent:**

Yes, it was. Actually, whether it was expensive or affordable, all I know is that I was able to pay because I needed treatment.

**Interviewer**

So, do you think anybody in your situation can be able to pay for the services?

**Respondent:**

Yes, anybody in my situation can go for treatment but if you don't have the exact amount then you can make part payment and complete later. I know of one guy in this area who was also in the same condition as mine but when he paid for the treatment, he's not cured.

**Interviewer:**

Please do you know his name?

**Respondent:**

He's called brother Kojo.

**Interviewer:**

Have you heard of clinical psychologist?

**Respondent:**

No, I haven't heard of it.

**Interviewer:**

What of psychiatrist?

**Respondent:**

Yes, I have.

**Interviewer:**

What of social worker?

**Respondent:**

No, I haven't.

**Interviewer:**

Do you know that people or the health personnel that are treating you?

**Respondent:**

Yes, doctors and nurses [treat me].

**Interviewer:**

Have you ever had the services of a psychiatrist?

**Respondent:**

No, I have never received services of a psychiatrist.

**Interviewer:**

What of the services of a clinical psychologist?

**Respondent:**

No, I have never received the services of the psychiatrist or this psychologist because it isn't that. My condition is not mental illness. It is something they have caused to afflict me. They have tried to use for money making spiritual powers. It is not that I am mad, but my condition is spiritual.

**Interviewer:**

Okay, do you know the people who are looking after Seth and co, who supervise them?

**Respondent:**

No, I don't.

**Interviewer:**

We want to move from here and talk about the training of health workers but we now understand that you are not mentally disturbed.

**Respondent:**

Okay

**Interviewer:**

So do you know how the health workers are trained and licensed to operate?

**Respondent:**

As for that I can't tell or in short I have no idea about that.

**Interviewer:**

Okay, have you heard of the mental health law?

**Respondent:**

No, I haven't. I was part of workshop where they told us about a new mental health law, but I really do not know the details of it or how it can help us.

**Interviewer:**

Have you also heard of any mental health policy? That is how did should be handled when brought to the clinic and how did should be treated when they're not well.

**Respondent:**

No, I haven't heard of it either. Except the meeting [workshop] we attended and they told us about all of these and our rights.

**Interviewer:**

We now want to go into how the treatment and services are paid for. So do you know how the treatment and services are paid for?

**Respondent:**

Actually, I don't have much to say concerning this. All I know is that the doctor has to prescribe my drugs for me and I also have to pay.

**Interviewer:**

Do you use the health insurance for your treatment services?

**Respondent:**

I don't have a health insurance.

**Interviewer:**

So how do you pay for your health services?

**Respondent:**

They usually prescribe drugs for me to buy at the pharmacy because the drugs are not usually available at the hospital always.

**Interviewer:**

What of the injections?

**Respondent:**

For the injection, I usually take it in the hospital with my own money.

**Interviewer:**

So how are you always able to pay your bills for the injections?

**Respondent:**

Actually, I don't know, but I think it's by the grace of God Almighty.

**Interviewer:**

Do you get assistance from anyone for your injections?

**Respondent**

Yes I do. But not all the time and I also get the assistance from my father.

**Interviewer:**

Do you also get assistance from your mother?

**Respondent:**

No, I don't get any assistance from my mother because my mother doesn't have money to assist me.

**Interviewer:**

How about your sister? Do you also get assistance from her?

**Respondent:**

Actually, I have never imagined my sister paying or assisting me in paying my hospital bills.

**Interviewer:**

Okay, how about the doctors and the nurses who are treating you, who pays them?

**Respondent:**

I don't know but I guess it's the government who pays them.

**Interviewer:**

Okay, is there any other way by which you get support from other sources from mental health coordination?

**Respondent:**

As for this I can't tell.

**Interviewer:**

To you, I mean I want to understand from you does anybody who needs service from you gets it?

**Respondent:**

As for this, it depends on the person because everyone has his or her personal problems.

**Interviewer:**

Okay, on the average is it more people who will be able to afford for hospital treatment or less people?

**Respondent:**

Actually, the opportunity is there for everybody but it's possible some will get financial back up and others will not get the financial back up.

**Interviewer:**

Okay, we are [I] very grateful for your responses. Do you know the information they collect from the hospital because they usually ask you some questions so do you know what they use it for?

**Respondent:**

Laughs.... I feel well treated at the hospital by the health personnel's over there.

**Interviewer:**

Okay, I assume that, to the best of your knowledge that's what you have in mind.

**Respondent:**

I also have a question for you (laughs)

**Interviewer:**

Don't worry, when we get there we would give you the opportunity to ask all your questions. Has the doctor who is looking after you ever asked you the way they treat you and whether they can improve on it?

**Respondent:**

Actually, the doctors are really trying their best for instance, whenever I go to the hospital, Doctor xxxxxxxxxx always treats me well as the way he should.

**Interviewer**:

Okay. So is Doctor xxxxxxxxx a senior doctor on mental health aspect or he is a doctor in general who treats all diseases?

**Respondent**

He is a doctor who treats all diseases. The problem I have is that, whenever you don't have money and you go to the hospital, you don't usually get the treatment as required.

**Interviewer:**

Have you ever been to the hospital to give them a feedback, so they can improve on it?

**Respondent:**

No, I haven't

**Interviewer:**

Have you also been to the hospital to seek for financial assistance?

**Respondent:**

Yes, I have been to the Korle Bu Teaching Hospital.

**Interviewer:**

Was your illness about mental health problem?

**Respondent:**

No, I have never gone ill with mental illness. Sometime ago I went to the hospital but I didn't have money at that time so the doctor assisted me at that moment.

**Interviewer:**

So did the doctor give you money?

**Respondent:**

He didn't give me money but he rather gave me all the drugs I needed.

**Interviewer:**

The services that are here, I mean the new integrated hospital, do you think its available in the community so that everybody can get or even other communities?

**Respondent:**

As for this, it is not common because it is not all the doctors that are good. Some will see you and frown their faces but for those who are God fearing, they usually give you their attention.

**Interviewer:**

How can we make the service available to all other people who needs it?

**Respondent:**

What I have to say is that, the health personnel's should be considerate in a sense that if a patient doesn't have enough money to pay his or her bills they can permit the person to make part payment so that when the person gets the money the person can then complete the payment.

**Interviewer**:

Okay, Have you gotten any other suggestion that you want to make?

**Respondent:**

What I have to say is that, you should walk with God, be considerate to people and also help the helpless. Laughs...

**Interviewer**

Okay, apart from walking with God, being considerate and also helping the helpless, I want to find out from you how you involve stakeholders in the organisation and delivery of mental healthcare services at the community level?

**Respondent**

We do what we can. Where it is possible to include other nurses, we do. But as you know they are usually not interested. A few times, when we participate in workshops, we try to ask for increased support, especially from the [district] assembly. In our community visits, we encourage families of people with mental illnesses to be more supportive of the clients.

**Moderator**

Please, explain more how you get families to be empowered and actively participate in how mental healthcare services are organised and delivered?

**Respondent:**

Hmm, we do our best even though more can be done. For individuals and families who are already our clients [mental health service users], we engage them and work with them to support themselves and their families and communities to ensure they support their recovery and functioning.

**Interviewer:**

Thank you so much. Do you have any other thing to say?

**Respondent:**

In addition to what I said earlier on, I want to say that we should always think good concerning one another. So that God will bless us.

**Interviewer:**

Have you ever had a conversation with someone like the way we are doing?

**Respondent:**

I have ever had a conversation with pastors and you know pastors can also educate me about things in the spirit.

**Interviewer:**

Has there been anyone here conducting research and wants to interview you?

**Respondent:**

No, there hasn't been anyone as such.

**Interviewer:**

Okay. We are now ready for your questions.

**Respondent:**

My questions are based on financial assistance, so I need money(laughs)

**Interviewer:**

Okay, we are hoping that the report we are going to write will draw the government attention to people like you so that they can support you. We thank you so much for your time but for spending your time with us we just have a token of GH¢10.00 for you.

**Respondent:**

Okay. Thank you very much and may God richly bless you.

| **Name of Interviewer/ Facilitator** | xxxxxxxx |
| --- | --- |
| **Name of note taker** | xxxxxxxx |
| **Type of participant** | Health service manager |
| **Gender of participant** | Male |
| **Disability of respondent(s) (physical or psychosocial)** | N/A |
| **Age of participant** | Not asked |
| **Community Name** | Sunyani |
| **Interview date** | 25^th^ August 2020 |
| **Duration of interview** | 28min. |
| **Recorder number** | VN870107.MP3 & VN870108.MP3 |
| **Name of file (follow convention below)** | Transcript individual interview. Mental Professional.docx |

**Introduction**

This individual interview was held with a Health professional (xxxxxxxxxx) at the Ghana Health Service in the xxxxxxxxxxx Region of Ghana. The participant was selected for this interview because he is a health professional and responsible for the xxxxxxxxxx. The interview was in English. A digital voice recorder was used to record the discussion and which were complemented by handwritten notes. The details of the discussion and written up below

**Interviewer:**

Thank you for the permission granted me. My name is xxxxxxxxxxxxxxx, I'm doing a thesis research on integration of mental health care at the community level in Ghana the role of the health system framework of the WHO. As a xxxxxxxx and one of the regions that am carrying out the research I wish to get your perspectives on this subject. So...

**Respondent**

Yeah

**Interviewer:**

I wish to kindly ask you to (silence, Participant having short conversation with another) so if you could kindly introduce yourself, your position and ehh what you do on a day you day basis and for how long you've been working in this role.

**Respondent**

My name is Doctor xxxxxxxxxxxx, Am currently the xxxxxxxxxxxx the Bono region. My role in health service delivery is actually can be found at three levels xxxxxxxxxxxxxx.

**Interviewer:**

Thank you Sir

**Respondent:**

We resolve issues at the districts level and we also make sure there is harmony between the staff working at the various districts, we build up capacity of the staff by implementing policies and workshops that are geared towards of quality of care and also geared towards prevention of diseases, all sort of diseases. Really, it's my vision to make sure that preventable and avoidable deaths are brought to the minimum to the very minimum using the structures in the Ghana Health Service.

**Interviewer:**

Thank you so much

**Respondent:**

Basically that's my role

**Interviewer:**

Thank you thank you. I will be touching on this because erhh I want to explore Mental Health Service provision, human resources, legislation, financing, erhh research development, information, data generation, leadership and governance, and integration of Mental Health Services.

**Respondent:**

Good

**Interviewer:**

So I wish to start with the services. What services are available for treating mental health conditions ahh in community based health facilities in the region?

**Respondent:**

The Ghana Health Service has a structure and the unit of the structure is the CHPS concept and in this region we've made sure that, almost all the sub units or subdistricts have mental health personnel and which have been termed as community mental health personnel taking care of the CHPS or taking care of the communities. So it’s, the that idea is cascaded or is replicated at the community level the erhh erhh this thing what do you call it the district level, no from the community we come to the sub-district level, the district level, now to the regional level and we've paid particular attention to that and make sure that even at every facility at the CHPS compound we designate a bed for the mentally ill. The sub-district a bed for the mentally ill, the district hospitals they are advocating that they have at least four beds for the mental health unit, dedicated to the mentally ill. And then at the regional hospital level, ten to twenty beds. Currently at this regional hospital we have about twenty beds capacity that have been designated for the mentally ill patients and the structure is such that we have a mental health coordinator for the region and every district likewise in this region is also being manned by a mental health, district mental health coordinator. That's the governance aspect and they actually control or delegate and manages the mental health activities at the district level supervising the sub-district who intend also go to supervise the CHPS compounds. So the structure is well laid and we receive report, I receive quarterly report from the regional Mental Health coordinator and during every annual report, and he is featured and he brings about their challenges so far as mental health care is concerned, so far as mental ehh quality of care for the mentally ill is concerned. Well to add it to, I'm the chairman for the Region Mental Health Committee; so we came up with a solution that ehm all the committee members had districts allocated to them as parents, so they are parents of some districts where they will go around look at the mental health situation in those districts and come and report to the mental health committee at the regional level when we ever have a meeting. Also we were in collaboration with all the faith based facilities as well as non-governmental ehm institutions and healing places, eh places of healing. Whenever we have meeting, the Mental Health Committee has a meeting we make sure we invite people who deal with ehh, who use herbs, the herbalists and the faith based healers to our meeting annually whilst we listen to them, listen to their challenges and in so doing we also impact to them. The fact that they should deal with the mentally ill with respect and the fact that we should have a collaboration with them, we show them that there is a ehh there are two aspects of the mental health some being very physical and will need health care at the health facilities and the spiritual aspect they can take care of that. So we encourage them to refer their patients to us and we try to tell them not to abuse the mentally ill by chaining them to trees and sometimes starving them, not giving them food for days and sometimes even trying to beat out according to them, the devil from them because they think the devil has possessed them that's why they are mentally ill some instances. In this region also, we also came out with a suggestion actually, it wasn't a suggestion it was a proposal, a written proposal budgeted for and everything to really take all the mentally ill patients from the streets. We were basing that on the fact that research had shown that 80% of the mentally ill patients who are walking on the streets can be cured and reintegrated into society. So based on the findings on that research, we put up a memo and we drew out activities to take out the mentally ill from the society in bits.am sorry to say that we never had funding we sent the proposal all over the place but up to now we've not had the funding to kick start that proposal. The idea was to take, you see this region have about 30 something mentally ill patients, we counted them and then we look, we are located or we look out for wherever they are staying to renew their sleeping places renew their eating, renew where they go to rest in the night, so we were going to use that to with the you know the ehh the the necessary laws backing us , and the police collaboration we were going to take them out of the streets about five at a time at the regional hospital we treat them, and when they get better, we move them to where we designate as a half way home where they will be taught trade and other aspects of reintegrating into the society and from there you know through the community health nurses were going to send them back to communities that they come from. Having used the community health nurses, to engage the communities in durbars, to actually make sure stigmatization is minimized or eradicated completely before we send the patient in and also to make sure that the family in which they are going actually embraces them back and takes them in as if they were like people who had just had any other disease like malaria and they have been cured of the disease how we receive they receive them back. We had all these plans in place but is unfortunate that we have not been able to ehn really push forward the plans because ehh all the institutions were sensitized the police, the army, the market women, the teachers, everybody RCC ehh this thing ehh the veterinary, forestry, they were all sensitized as to the plight of the mentally ill and the need for us to take the mentally ill patients as people who have suffered any other disease like malaria and so on. So that's what we do.

**Interviewer:**

Thank you so much Sir. I'm very grateful, that's very comprehensive. Sir, I want to focus on the eh community level and I guess that's CHIPS and sub-districts level

**Respondent:**

Yes

**Interviewer:**

What exactly are the mental health services provided at that le, at those two levels?

**Respondent:**

Those two levels, we provide creative as well as preventive services at those two levels. As I was talking, I mentioned durbars, normally durbars are created you know at that level and education given to the community. As to what is mental health? what are the ramifications of mental health?, what are the inducers of mental health? and so on. So those are the services provided and then also, at the community level those who have been treated; who are known to be having mental illness and have been treated and are in the community, the community health nurses and the community health officers make sure that they do no you know, what is the word?,ehm they did not, they always take their medication and do not to and they do not there's a word,

**Interviewer:** default?

**Respondent:**

ehhm default from their medications and also we sort of sometimes give pieces of advice when we see that one particular patient is defaulting and maybe going back to the old habits we call them in and give them specific advises. And also when there is acute sicknesses mental health sicknesses those are the people at that level, they've been taught how to give them medication you know to bring the ehh so we call the acute illness as some sort of emergencies you know we've taught them to actually give them medication that would calm them down and then refer, so the referrals system takes place between the CHPS compound to the sub-districts, the sub-district to the district hospitals who so if unable to manage, they also bring it to the regional hospital, regional hospital we have, even though we don't have a psychiatrist, we have mental health officers and psychologist there who will put the patient through. And we also have the services of psychiatrist from the ehm this thing the Teaching Hospital who normally Kumasi ehm Okomfo Anokye who normally comes to visit us to render services. So those are the services that are rendered at the sub-district and district level ehh and then the CHIPS compound with the help of CHOs and then community mental health nurses.

**Interviewer:**

Thank you Sir

**Respondent:**

mmm

**Interviewer:**

Very clear

**Respondent:**

Alright

**Interviewer:**

So ehm you've talked about a bed for the CHPS and a bed at the subdistrict level and then ehh extra beds about four to five at the district

**Respondent:**

Yeah, yes

**Interviewer:**

And over twenty at the district level

**Respondent:**

Yes

**Interviewer:**

With this approach, is it based on on ehh a certain document or policy requiring of ahh the various health managers to comply or is just based on what you see is appropriate to do?

**Respondent:**

No, no there was a document, a document was sent that stated that all regional hospitals should have between ten and twenty berth capacity allocated for mentally ill. District hospitals have up to five berth capacity and in every community at least there should be one berth allocated for the mentally ill. So is not from my whims and caprices that am bringing that, there was a document sent from the this thing ehm Mental Health Authority, in fact they brought that when they were looking at the ACT 526 or so for the Mental Health act when they brought it and they were disseminating the contents of the ACT.

**Interviewer:**

hmmm

**Respondent:**

that's when I might have gotten the number, the figure a little

**Interviewer:**

846

**Respondent:**

846 yeah. I know is 8 and there's a 6 but what was in the middle (both laughs) 846 yeah. When they brought it, and they were asked to disseminate the content,

**Interviewer:**

Yeah, yeah

**Respondent:**

A letter came also informing the region to make provision for all this thing.

**Interviewer:**

Okay, how about the levels of service provision from CHPS to subdistrict to district to regional. Is there a document or guideline like standard treatment guideline available?

**Respondent:**

Well, ehh you just a minute. (phone call comes in)

**Interviewer:**

okay Sir

**Respondent:**

Mm him so the level of service provision have been spelt out as to what can what type of drugs can be given at the levels of CHPS compound, the subdistrict, the district and then at the regional level. I think they have the documents to support it.

**Interviewer:**

Do you have a copy?

**Respondent:**

No, I don't have a copy. But I think the regional mental health coordinator would have such a copy and it explains how and even the staff who are there have a limit, they have a limit to the way they structured their education. We have the community mental health nurses and from there we have the mental health nurses themselves, mm then we have the mental assistants. You see? and then we have those who have diploma in mental health

**Interviewer:**

Services

**Respondent:**

And I think we have those who have even degrees, mmm?

**Interviewer:**

Yes Sir!

**Respondent:**

Before we come to the psychiatrists who are medical officers themselves and unfortunately we don't have them at the district level but some regions have them but we don't have them at this region. We use to have one mm who died mm Dr Dones I think you might have heard of him.

**Interviewer:**

Yes Sir

**Respondent:**

Techi Dones

**Interviewer:**

Yeah, Tachie Jones

**Respondent:** mm yeah

**Interviewer:**

Thank you Sir, so how accessible are mental health services to the population in the region?

**Respondent:**

I think in this region we've done alot and mental health service is as accessible as any health service delivery. Is they are all along the lines I told you about the CHPS which is in the communities then the sub-districts mm then the district level. So every you go service is available

**Interviewer:**

mmm

**Respondent:**

And it's accessible

**Interviewer:**

mmm

**Respondent:**

You see? and even we've gone further to make sure that ehm we minimize the financial challenges associated with accessing health in general by making sure that ehn all the mentally ill patients have been enrolled onto the National Health Insurance we did that district by district and I think some MPs, here I must say that some MPs did well help out in giving out the money to enroll them onto the National Health Insurance.

**Interviewer:**

Mmm

**Respondent:**

So mental health is accessible as any other health service delivery. But I must quickly add, that the knowledge in the communities as to how accessible the mental health is not actually there even though we've done a lot we've done a lot (someone walks in).....

**Interviewer:**

So we were talking about accessibility,

**Respondent:** mm

**Interviewer:**

You said accessibility is available, I mean mental health services are accessible,

**Respondent:** mm

**Interviewer:**

Virtually throughout

**Respondent:**

mm hn

**Interviewer:** So I wanted to find out, how many of your sub-districts happen to have units?

**Respondent:**

You mean mental health units?

**Interviewer:**

Yeah

**Respondent:**

I said it already

**Interviewer:**

mmh

**Respondent:**

That all of them have mental health units

**Interviewer:**

Okay

**Respondent:**

Being manned by mental health officers and community mental health nurses

**Interviewer:**

Thank you Sir, and how affordable are they to the population? How are mental health services affordable to the population?

**Respondent:**

I think I answered that question also separately because, you know mental health services are not being are charged for mmh and the little other diseases that the mentally ill may encounter which can also be taken care of by the health insurance, we've to make sure that the mentally ill are enrolled onto the Health Insurance scheme. So virtually, they have their treatment free of charge at no cost even though the mental health medicines like the anti-psychotic medicines are very expensive, but those are programmed drugs that are given free of charge and they are not supposed to be sold. So eventually or definitely it makes the mental health treatment for mental health illnesses free.

**Interviewer:**

Do people pay for mental health services?

**Respondent:**

Not here, not that I know also.

**Interviewer:**

So I want to look at your human resources which have touched on we've talked about community mental health nurses, ehh mental health nurses, mental health assistants, degree nurses and the psychiatrists.

**Participant**: mmh

**Interviewer:**

What are the numbers of people who are operating at the subdistrict level providing mental health Care services?

**Respondent:**

Really really ehm, what I know at least there a mental health nurse in every electoral area

**Interviewer:** or in every

**Respondent:**

or a mental health nurse is attached to at least to every electoral area or every community

**Interviewer:** mmh

**Respondent:**

And then at the district hospitals, there are mental health units where we can find two or three or more mental health officers, one two three or more mental health officers as well as other cadre of workers who help the mental health officers at those facilities.

**Interviewer**

mmhh, thank you Sir

**Respondent:**

But I can't come out with the numbers but ehh the mental ehh regional mental health coordinator has the data and I think he will be able to provide you how many are where at any particular time.

**Interviewer:**

Yes sir

**Respondent:**

mmm

**Interviewer:**

How about training and accreditation of community mental health or mental health professionals who provide services at the community level. How are they trained and accredited or licensed?

**Respondent:**

Oh they are trained from the, you know we have facilities that train mental health officers and mental health nurses because there were times where community health nurses applied for and they were given ehh study leave to go and do mental health nursing. Kintampo, Kintampo is one, which has the facility for ehh training mental health officers and when they come out, workshops frequent workshops are done. It used to be quarterly until the mental health, the subsidy from the mental health authority was no more coming in. But they were having workshops every quarter, to refreshen their memory. And then also, when reports come in, we see some gaps in their reports. We use that to form or to come out with a teaching tool to address those gaps. And we were doing it almost every month for them until the motivation no longer existed and went down. But still they are supposed to bring their quarterly reports. And whenever we see gaps, we send people who go down there for supportive supervision and deliver services on the job training, coaching services. As for their promotion, they go the same way as nurses, other nurses they get promoted to the senior principal and chief deputy chief and but then they will have to be also get, they will have to also get appraised by their our superiors. Mm. And the head of institution gives their final signature to the appraisals.

**Interviewer:**

So ehh I did hear you talk about, uh, the mental health workers in each unit. I was seen by a coordinator. Ah. I just want to confirm, uh, how are their appraisals and performance determined?

**Respondent:**

(phone rings)... Uhm just as I was saying, where were we?

**Interviewer:**

Their performance

**Respondent:**

Their performance are appraised by their, the head of units. Actually, I did not, you made something I want to correct but it skipped me. Now, their performances appraised by their coordinators who intend send them to the head of the units and they also appraise them mm. Two that's how it's ehm they get appraised of their, you know how appraisals are done, I don't know whether you know the strategy in Ghana Health Service, at the beginning of your, every year ehhn ehm the work employees eh, are supposed to meet with their superiors those who supervise them and come out with a working plan: what they have decided to do during the year. Like for instance this year I want to do about twenty home visits is just an example I'm giving, I have to conduct about thirty durbars mm at the CHPS compounds I have to conduct about thirty durbars, I have to give information to people about this, I have to check on this, you know you bring out the plan. A plan with objectives, measurable objectives. mmm So mid-year, you're brought in again and we access to see what are the achievements that has taken place, so we look at if you said at the beginning of the year you're going to forty eight home visits, we then check how many home visits have you done. Where is your book? your home visit book and how many people did you meet? What is their number? what was discussed between you and the people you met? Then you check to cross check by calling the people to see whether you actually visited them. And you give the talks, you did the durbars, the attendance sheet is important, we look at it. So let's say you said forty eight at mid-year, have you been able to do twenty four of the home visits? if yes, then there is a comment after we've found all the parameters to make sure that you really did and exceeded twenty four. There is a comment column. Ehm we think that if half years you've done twenty four or more, then is likely you would achieve your target at the end of the year. So we write at the comment during half year, target achievable mm. And we have a system of grading 1,2,3,4,5 it at the beginning, at the end of the year, you've not done anything at all about that objective, then maybe you're supposed to pick at least between three and five objectives and you know objectives that are measurable and you know objectives is normally broken down to activities and those activities are all coated mm and can all be measured to know that you wanted to do this and this activity; have you done them? How many have you done? How many did you say you will do? If you haven't anything at all we will give you 1, if you've done about 20% of them then we will give you 2, if you've done about 40% of them we will give you 3, 40%, 50% we give you 3, if you've done about 80% we will give you 4, if you've done all at the year we will give you the 5. And we have a way of computing it to know whether you fall within excellent, very good, good, satisfactory or poor. We have a skill for it. I wish I had the form here for you to see how it is structured. So we use that to appraise you. To see that this year, yes you did well, you did very well, you did excellently, you did satisfactorily, well you were good just good or you were poor, if you're poor, then at the end of the comment we say you're poor this year so you did not need to be promoted even if you're due, due you can't be promoted even if you're due. And if you did satisfactorily, we say yes you did okay, satisfactorily, so even if you're due for promotion you should for another year. But if you're good, from very good to excellent then we can say if you're due for promotion, ehh very good and good you can say if you're due for promotion, you should be promoted. And when we reach where you're excellent, then we can say this guy has can be promoted out of ten even though you're not due for promotion you're left with about one year for promotion but because of how you've worked and how the assessment has gone, you can be promoted before your time, you see, so that's how we work in Ghana Health Service here and those are laid down fundamental working tools. That we always do.

**Interviewer:**

Okay thank you, sir. I'm very grateful.

**Respondent:**

You're welcome

**Interviewer:**

Okay. This is the issue between mental Health Authority and Ghana Health service and staff? Are there any mental health authority staff that you manage?

**Respondent:**

Mental Health Authority staff that we manage here?

**Interviewer:**

umm

**Respondent:**

You see, mental health authority has not come out with so to say it's own staff, you know. they really poached on Ghana Health Service staff. There is no budget line saying that these are Mental Health Staff. Those were the people, the people they depended on and they were using were already Ghana Health Staff. So it's like they're already there in the service, and then they were using them. But, you know, you and me know that mental Health Authority has not chart-out organizational chart. That's what they want a clear line. If they say they own staff, their own structures, everywhere they are depending and using Ghana Health Service staff and structures. So the mental Health coordinator, for instance, is under clinical care being supervised at this level under the clinical care department community. So is the clinical head, which is a deputy director clinical care who appraises and you that is what you call control, who appraises the mental health Regional mental health coordinator.

**Interviewer:**

Do you have, um, a sense of some staff feeling they belong to mental health authority in the region?

**Respondent:**

A sense of staff, some staff feeling they belong to. Well, well, well, you know, this is a funny question, but the mental Health Authority really has not come out boldly, but we have instances where the mental health coordinators in the regions feel they don't need to report to the directors. That is, in some regions, but not in this region. We've had that challenge through the regional clinical care team and also even through to the National Clinical Care directorate. They feel they owe allegiance only to the mental health authority because those days mental health authority was given bringing some small, small monies along for them to have their meetings and all those things. So they felt they had to report to them without recognizing made the mental health unit at the national level. We had that challenge, but not in this this region this region it never came to that. Nothing like that. So this region to tell you we don't have any staff who feels, he is a staff, he or she is a staff of mental authority they know they are staff Ghana Health Service working or collaborating with Mental Health Authority

**Interviewer:**

Mhm. Thank you, sir. One more question on human resources and we move on, in the ideal situation, what should be the staffing mix at each level for mental health care service delivery?

**Respondent:**

Then this question I will skip because I haven't thought of that and I don't think that we've had a policy on it and ehh a real directive as to what should be the mental health mix at a mental health unit. But I can hazard a guess or what I think should have been appropriate. But since we have not had a policy or if we have not sighted it, I cannot really speak to it. I will know that in every unit and for instance, we know that Ghana Health Service we do not draw clear lines between services, but at the regional level, if you want to look at the staff mix, then I'll say there should be a psychiatrist, there should be mental health assistant, which could be a degree holder and then we had one of them. The psychologist, uh, BSC psychologist in psychology. People came out with that idea. Yeah, those ones were they? And then we had the community health nurses, mental health nurses which could be for a hospital level. They could be about four or five, and then we also had supporting staff who were sometimes enrolled nurses who have not had this thing ehh the training to become mental health nurses then we have community health nurses also in that unit at the regional level. As you go towards the district and the CHPS, some of the people are dropped. For instance, here, if you go to the district, there's no psychiatrist but there is a mental health nurse assistant, and there could be a man ehh we used to call them psycho something psycho. Uh huh. In psychology, psychology or something from in mental health, uh, they are there. They at the district level. And as we go to the subdistrict, we have the community mental health nurses who are working there, as well as taking care of the CHPS supported by enrolled nurses and community health nurses

**Interviewer:**

Thank you, sir.

**Respondent:**

You're welcome

**Interviewer:**

We've touched on the mental health law.

**Respondent:**

mmm

**Interviewer:**

How do you see the mental have law promoting community mental health services?

**Respondent:**

Well, I think uhn it's actually touches the points that are I needed to be touched on, but as usual, Ghana we come out with fine laws, fine policies by the implementation on the ground needs much to be desired for often for all you know, the law promotes mental health activities and mental health service at the community level. But when they are not resourced, the community mental health nurses do not get the morale or they are not motivated enough to really do the services as has been spelt out by the law. For instance, they lack motorbikes, and, you know, some of our communities are very hard to reach. You see, they lack motorbikes and sometimes they even the book the notebook to write in their findings. You know they need to have this back sack where they can carry basic medications. Sometimes the torch light the Wellington boots, the raincoats are not available and even resources is to buy fuel if they have the motorbikes is not forthcoming. So the laws are there and people are willing to actually do practice the service or take up the service to the doorstep of individuals. But then the motivation, the funding is the problem.

**Interviewer:**

How about a mental have policy? Is there a mental health policy espousing the community based care.

**Respondent:**

That have been sighted? The law I have seen that is the one we talked about. But the policy I think it is there. I think I saw something like that, I didn't take time to really glance through so much. But I think it should be there. Well, let's talk to the mental health coordinator and then we see how each also

**Interviewer:**

Yes, Yeah. And do you see the law promoting human rights of people with mental health conditions?

**Respondent:**

Yeah, it is the in the law, if you look at it that I said that respectful care for the mentally ill have been given by this thing and even the fact that I said because of the backing of that law, we were able to communicate Collaborate with ehh faith based healers and traditionalists who take care of the mentally ill advising them of the, you know, respectful treatment, avoidance of the chaining, the beatings. You know, making people go all the way to fast un-end on then, you know, trying to beat the devil. You know, I've mentioned those things we had those things all in mind because the mental health laws spelt it out that mentally ill patients should be treated with respect, just like any other person who is having any other disease of soft mental of sound mind.

**Interviewer:**

Thank you sir, I am very grateful. I know you also touched on funding. You always said you have made sure people have National Health Insurance

**Respondent:**

Yeah, Sure.

**Interviewer:**

As far as care is concern, no payments are made.

**Respondent:**

mm uh

**Interviewer:**

But where are the main source of funding for mental health? Especially community mental health?

**Respondent:**

The main source of funding is non existent. We thought the mental health commmun, authority was going to really resource those things, you know, But they fizzled out after one or two years. There's no funding, so there's no source of funding. And since you meant in mental health, you cannot I mean, generates any income then is slowly is ehh, you know, pushing the activities towards good and quality mental health service delivery to the ground.

**Interviewer:**

Okay, I want to touch on medical products

**Respondent:**

mhm

**Interviewer:**

And equipment for mental health care services, especially at the community level. How are they planned for, procured and distributed, particularly medicines?

**Respondent:**

As I said, medicines, uh, are normally considered as a programmed drugs so.

**Interviewer:**

What does that mean?

**Respondent:**

Programmed medicines means that the government settles and buys them and nobody else imports them and buys and distributes. Some NGOs do same, some development partners do same just like programmed drugs like, um, a this thing HIV drugs, TB drugs and all those things those are programmed drugs and even, you know, when it came, the Greenleaf for malaria was supposed to be a programmed drug but now material because it's so common, people have money to I mean get some aspects of it that are being sold on the industry. But then the Greenleaf are also subsidized. When it came in, they were supposed to be not more than five Ghana cedis, if you remember. Yeah, so what I'm saying is that for the funding and procurement of drugs and equipment, some funding came from the mental health authority at the beginning, when they brought in the law and the this thing, they brought in some funding for some minimal equipped, uh, Weighing scales and all those things. And then they try to procure some offices for some people. You know, the at the community level, some accommodation for community mental health nurses. But it fizzled out. Now there's no laid down level of procurement of any medicine or equipment for the mental activity. No, there hasn't been that for the past three four three years.

**Interviewer:**

Why is it so?

**Respondent:**

Well, I think ehh after the shouting, the fanfare about the Mental Health Act and the this thing, you know they are finding it difficult to get support either from central government or from NGOs. And also, they were unable to carve themselves out of the mainstream health services delivery. Mhm but that was their plan to carve themselves out to do their to have their own budget lines and to be able to create them for themselves, a service like Ghana Health Service, like the CHAG services. So we're going to have mental health service, the insurance, the CHAG and then Ghana Health Service all coming out from the ministry enforcement. But I think that didn't go through. And that's why they are cash-strapped and they don't have any funding for for all you know, they draw their budgets and everything, but the funding normally does not go through.

**Interviewer:**

Thank you, sir.

**Respondent:**

You're welcome

**Interviewer:**

Information and data collection.

**Respondent:**

Mmhm…

**Interviewer:**

What kind Of data is collected at the mental health facilities at the sub district level?

**Respondent:** What type of data?

**Interviewer:** Data or information around mental health level

**Respondent:**

Yeah, there are their level, at that level they come up with the total uhm number of people who are mentally ill in the community, the type of mental illnesses that they have, the number of drugs that got supplied to them and how those drugs were used. Then how many of them have recovered or how many of them have their this thing their, uh, disease states, you know, improved? Mm hmm. And then how many of them have defaulted. I mean, how many of them have died. So all those data are brought to us to analyze.

**Interviewer:** mmhm

**Respondent:** uhm

**Interviewer:** So what is the effort of the…

**Respondent:** the new cases new mental illness

**Interviewer:** …that have been registered?

**Respondent:** that we have been registered are also added.

**Interviewer:** I was about to ask uhn what kind of information is collected in at improving services?

**Respondent:**

In, oh well, they send information about that in the form of the gaps that are identified in their reports. When the gaps are in their reports, we use them uhn as activities that has to be done to improve the services. If let's say, it's recognized that yes there are some patients mentally ill patients that need to be visited but they couldn't be visited because of lack of motorbikes. In order to improve the visit for the person to render the service at that level you have to get the person the motorbike. If there are shortages of drugs, it comes out as a gap then we put in efforts so that there are no shortages of drugs. If hygienic materials are not available to bring in the this thing so that those mentally ill who are brought in and are supposed to be taken care of; you've to bath them, you've to do this do this you know make them hygienic and also you've to feed them, mentally ill can eat they eat a lot especially after the psychotropic drugs, they eat so much. So you need to really be there to provide them with the needs. So you see resources need to be put in.

**Interviewer:** mmmm

**Respondent:** yeah

**Interviewer:**

So is there an effort to get feedbacks from those who use uhn mental health services at the community level to understand how much they appreciate their services and what else can be done?

**Respondent:**

You see, we get ehm we get that from the caregivers but the mentally ill we do not get the feedback from them. We do not but the caregivers are those who can sometimes give us the feedback oh thank people your activities have made my brother become well and we appreciate that but then these were challenges that we are facing now maybe he is too quiet, locking himself in the room then we go in knowing that that is a sign telling you that something is not well, then if we get the feedback that he is now integrating into the community well, playing with friends, going out, doing the things they used to do together then you know even though you know that things are getting well, you still keep an eye on them. So the feedback are mainly gotten from the relatives and this suggests sometimes the things that they need or sometimes think if we can improve the services.

**Interviewer:** mmhn

**Respondent:** mmhn

**Interviewer:**

Is there any reason why they don't directly from the people who are using the services?

**Respondent:**

You mean the mentally ill?

**Interviewer:** mmmm…

**Respondent:**

No, sometimes you might not be able because ehhn you know, they don't have intuition into their state of mind at least when they were sick some of them will not know how they were feeling how they were face what they were facing (someone walks, little interruption) so they don't have insight into their condition so they might not be able to give you a full feedback. Some when they get better, yes they have their feeling they can give you feedback on that but when you know when we want a proper feedback accessing the person how they were, what how they have become and therefore the improvement thereof or the deterioration the health we can't depend on that.

**Interviewer:**

Okay. Is there is there any on going research on improving service provision at the community level that you know of?

**Respondent:**

No, non that I know of.

**Interviewer:**

Okay.

**Respondent:** Yes!, there is one I know of, this one you're starting.

**Interviewer:** (laughs)

**Respondent:** (laughs as well)

**Interviewer:**

Thank you Sir. As for monitoring, evaluation, we talked about supportive supervision earlier on but if there is need you, how are community mental health officers involved in supportive supervision activities?

**Respondent:**

Well, ehhn you know, they are mostly supervised and supported to deliver services. Mental health is not considered in matters at this level. Supportive supervisions take place, but mental health is not included. We are just sitting and watching. They may include you, but it is not because of mental health but that you an available staff. And we have grades of them, so those who are seniors support the juniors to deliver services. As for the juniors community health nurses, they can only do supportive supervision to the clients, to the clients they have you know, helping to take their medications regularly, giving them education so that they don't go back to their old ways you know and trying to help them assimilate into the community. And also helping the community, supporting the community to reduce stigmatization or maybe the community health nurses actually support and supervise the communities in which they are and the relatives are ramped the mental region.. So that's how I think, I understand.

**Interviewer:**

Thank you Sir. So sir, what is your estimation of the level of leadership and governance in the development of community mental health in the region?

**Respondent:**

Yea, there is a strong leadership and will but the push is not there. The push has been derailed because for one to be able to really exhibit your leadership properties on them, you need the push, you need to go down there, supervise them, you know, but the leadership they know there is a very high level leadership by way of the regional director and his ehm team as well as the mental health committee chairman and his members. We cannot say there is no leadership and governance of mental health in place at the Primary Health Care level. Increasingly, directors are taking an interest and through that the situation will improve.

**Interviewer:** Thank you Sir

**Respondent:** You're welcome

**Interviewer:** To my last set of questions, I know you're (laughs...)

**Respondent:** Set of questions, last set?

**Interviewer:** last set. It's on integration (laughs)

**Respondent:** Ohh then, maybe tomorrow

**Interviewer:** yeah, okay my last question

**Respondent:** uhn we continue tomorrow because ehhn is been too long a time, I need to work through these things and

**Interviewer:** laughs... No problem, so I you thank you so much for giving me the opportunity..

**Respondent:** Why do you have too many questions?

**Interviewer:** Uhn because I am following the framework

**Respondent:** Ohh

**Interviewer:** but that's fine I just wanted to

**Respondent:** Is too much, we've spent more than one hour

**Interviewer:** Yeah. Your perspectives on the integration but I think you've spoken in various ways

**Respondent:** Yeah I have spoken in various ways so when you go listen carefully you can get that one out

**Interviewer:** Yeah, Okay. So thank you so much. Do you have any question for me?

**Respondent:** Yeah, I will, one question is that when you finish the stuff, how are you going to disseminate the findings?

**Interviewer:** Ehn, my idea is that I will do a number of seminars on the study at the university on the study.

**Participant:** uhn

**Interviewer:** I also hope to do a regional dissemination of what I found

**Respondent:** mmh

**Interviewer:** Ehh in the target regions, this region will be one of them to give me some sort of validation that I thought if I captured everything well(laughs)

**Respondent:** mmh

**Interviewer:** So that it will help in my report. Ultimately, I'm hoping that the report maybe of interest to government as part of getting evidence based to developing mental health services at the lower levels. Is one thing providing evidence to government and is another they taking it and work with it but we hope that they will find it a useful document to work with. Yeah, so these are. So I'm still mobilizing and applying for funding for dissemination, regional level dissemination. Is important but sometimes is the funding

**Respondent:** laughs

**Interviewer:** you have to give them snack, rent a venue,

**Respondent:** yeah

**Interviewer:** people who are coming from far away you have to give them something so all that have to be captured in.

**Respondent:** uhm (yawns) do you intend to publish it?

**Interviewer:** Yes, it is a requirement

**Respondent:** Is a requirement?

**Interviewer:** Yes

**Respondent:** to publish?

**Interviewer:** yes

**Respondent:** So which journal are you aiming at?

**Interviewer:** There are several journals ehn

**Respondent:** I am saying which journal are you aiming at?

**Interviewer:** Am aiming at uhm at least two. I am aiming at BMC, am aiming at journal of social psychiatry,

**Respondent:** mmh

**Interviewer:** And I'm also actually looking at the Lancet.

**Respondent:** Why the BMG or BM

**Interviewer:** BMC

**Respondent:** British, is it the British Medical journal?

**Interviewer:** yeah, journal. BMG is a British Medical journal ehh, it's actually

**Respondent:** Which one is the BMC? British what?

**Interviewer:** Is, let me get, is one of the common journals

**Respondent**: Is BMK

**Interviewer:** British Medical Journal

**Respondent:** Yeah

**Interviewer:** Pardon...

| **Name of Interviewer/ Facilitator** | xxxxxxxxxxxx |
| --- | --- |
| **Name of note taker** | xxxxxxxxxxxx |
| **Type of participant** | Health Professional ( CPN and xxxxxx) |
| **Gender of participant** | Male |
| **Disability of respondent(s) (physical or psychosocial)** | N/A |
| **Age of participant** | Not indicated |
| **Community Name** | xxxxxxxxxxxxxx |
| **Interview date** | 27^th^ August,2020 |
| **Duration of interview** | 52 minutes 32 Seconds |
| **Recorder number** | VN87010115.MP3 |
| **Name of file (follow convention below)** | Transcript individual interview. Mental Professional.docx |

**Introduction**

This individual interview was held with a Health professional (xxxxxxxxxxxxxxxxx) at the Ghana Health Service in the Ashanti region of Ghana. The participant was selected for this interview because he is a health professional and mental health care service provider. The interview was in English. A digital voice recorder was used to record the discussion and which were complemented by handwritten notes. The details of the discussion and written up below**.**

**Interviewer**

So thank you very much for giving me the permission to record this ehh interview with you my name is xxxxxxx am doing a thesis research on the integration of mental health service at the community level the role of the WHO health system framework in influencing eeh the integration of mental health services at the lower levels of health care services in Ghana and you have been identified as one of the key informant to share your perspective on this subject so I thank you very much for agreeing to speak with me eerh this interview eerh I want to assure you of confidentiality that your name will not be mentioned as one of the respondents as you will be given a code eeerh there is also no right or wrong answer it's more about your perspectives and experience of being a health mental health service provider within these years so to continue I would like you to introduce yourself by giving me your full name eerh your position tittle eerh what you do on a day to day basis and for how long you have been involved in this position.

**Respondent**

Okay thank you so much xxxxxxx eerh of l for a start my name is xxxxxxxxxxxxxx a CPN at the same time xxxxxxxxxxxxxxxxxx I have been doing this job for roughly 15 years now, yeah 15 years now and basically what we do here is to take care of the referred cases from our mother hospitals like Ankaful, Pantang hospital eeerhm Accra psychiatric hospital these three major hospitals and other private mental health institutions when they finish seeing to the patients they have to call on us if the patient is from our district and we take over in maintaining the patient as in eerh home visiting eeh tracing of defaulters being social eerh eh let me say we intervene on issues concerning our clients and eerh we virtually also take care of those who are eh first time having mental problems lemme say if they were not able to take the patient to our mother hospitals we will have to take care of them here if they respond very well that is it we continue with treatment after two weeks if they are not responding the way we wanted then we are supposed to refer back to our mother hospitals for further treatment and then brought back the community so basically eeerh eh that is the major thing so we basically treat all the mental health conditions, we do treat all the mental health conditions yah

**Interviewer**

Thank you very much, so on a day-to-day basis on a day to day basis in your role what exactly do you do

**Respondent**

Okay here let me say eeh you're talking about me not the unit as a whole

**Interviewer**

Yes

**Respondent**

So eeh yah I have a role by going round and checking on our sub districts to see if our nurses there who are in the subzones have had contact with any of our mental health eeh cases if they have I need to take as in a report because every month I will have to write a report and submit to my regional coordinator through dims because we need to accumulate records and we do what we call I do what I call home tracing for a very long time I can't find eeh a client coming to visit or they have not been coming to their normal routine review I will have to find out why so we do what we call defaulter tracing with the those who do not come on time like they don't come for review we call them defaulters so we trace them and locate them probably there might be a problem or eeh they are probably having financial difficulty so when you get there yourself you will get to know then any assistance you will give we do health education we go to the various schools within the district sometimes beyond the borders since we share borders with the eastern central and western corridor sometimes we have to go beyond our stretch and give health talk to those communities and schools closer to us and eeh I also go for what we call self-help group meetings where we have clients and caregivers coming together as a group and eh I join them so I could also hear their problems and some of the challenges that they are facing and we do these meetings quarterly but they do I go there quarterly but they do it according to their own time and schedule ooh and the normal consultation they come we assess patients and we clerk and put them on medication basically that is what we do social intervention like step in where client and relatives are having challenges or difficulty at home maybe a client is maltreated at home and needs our help the clients can come around and talk to us we will go there and we will sit the family down and talk to the family let them understand why the patient is behaving this way and what they can do to help the patient so basically that's what we do .

**Interviewer**

Thank you so much, so I want to understand what mental health services are provided at the eeh community psychiatric unit in the district or even at the lower levels of the health care services at the district level what are how are mental health services okay what mental health services are provided at your unit

**Respondent**

Basically that is what I spoke about as in the review of referred cases that is the major duty so that is the number one service meaning that you come we see to you we assess if you're not, if you're improving or you're probably relapsing then we we give due medications if there's a need to increase your dose or decrease your dose and those stuffs the we follow up and go and see probably ther might be an issue at home that is why you're defaulting or you're relapsing we follow up and go and check on those stuffs yeah

**Interviewer**

So describe to me eerh how are mental health services structured from the community to the highest level

**Respondent**

Mmhmm, from the community what I know is when we when we when I personally liaise with my regional coordinator, whatever activity that we undertake here I need to send a report to them and she'll also send it to the national so like that is the organogram that we use then in the sub district we have what we call the community mental health officers they also get into the community and report back to me acting as the district mental health coordinator then I'll also follow up and send a report there so that is the chain

**Interviewer**

Alright so I could come back but I just want to find out at each level of the health care services provision do you have levels of care you give in these in each level right from the local through to the or sub district to district to regional to eeh tertiary level

**Respondent**

If am getting you right you see eerh are you do you

**Interviewer**

What you have talk about is more of reporting but I want to understand eeh treatment

**Respondent**

Treatment, Okay so like just like you said in the sub district the community mental health cannot give certain medications unless at the district level so if you find a case you're supposed to bring it to the district level of it's critical where we'll have to come there then we'll but if not we'll guide you like telemedicine do this do this and bring the patient to us then I'll admit here observe and treat the patient accordingly but there are no protocols strict protocols to eerh don't give this medication unless in a general setting where in the sub district you can't go beyond certain medication here it's not like that eerh but eh I have to make sure as the head of the facility whenever a junior colleague is clerking it has to be done under supervision I have to supervise because should anything happen I'll be held responsible but we don't have any restrictions to medication as in you cannot prescribe this medication you cannot prescribe this medication.

**Interviewer**

Okay. In that case is there a document outlining what services can be provided at each level

**Respondent**

No.

**Interviewer**

Okay alright that's fine eeh I want to find out how accessible are mental health services to the population on your perspective?

**Respondent**

>Sighs< For the populace it is quite difficult quite difficult if I have to travel let's say eeh one and half hour to get to a sub district and I find it difficult it's going to be virtually very difficult very difficult for a client cos I'm using my vehicle she has to go through he or she has to go through the public transport and even the fare even involved is so high that fare can buy that client that same medicine with a change so why would that pa patient want to come so it is very difficult to access cos we're far apart the sub districts are very far apart, the villages are very far apart the roads are not are unmemorable very difficult to use

**Interviewer**

I realised that you didn't mention the district from where we're having this interview.

**Respondent**

Yeah, Adansi sub district

**Interviewer**

Adansi sub district

The last district in (laughs) Ashanti region, the poorest

**Interviewer**

Is that so?

**Respondent**

Yes per statistics the poorest district

**Interviewer**

I see I didn't know that (laughs)

**Respondent**

(Laughing) well I'm telling you today

**Interviewer**

I thought it's a cocoa growing area

**Respondent**

It is a cocoa growing area but their season it's seasonal eh yeah

**Interviewer**

And there's a lot of timber too

**Respondent**

Yeah but unfortunately eeh the community does not really benefit from it yeah

**Interviewer**

So that case are mental health services affordable by your estimations

**Respondent**

No it is not, I'm telling you the truth it is not affordable, if for about three four five years you get once medications passing through and those medications

**Interviewer**

Medications from government?

**Respondent**

From government and those medications finish in 2 weeks 3 weeks’ time cos I have 3 thousand plus patients how the district the community psychiatry was just started 2011 so look at the time frame let's say roughly nine ten years and is we have three thousand plus patients and we can't afford to take care of 5 hundred at a whole with medication supply fully for the month it tells you it's a problem, it's a big problem they'll have to buy most of the medications themselves so it's the private pharmacy shops that makes the money.

**Interviewer**

Are they any other non-drug services I do know you talked about home visits , you talked about sitting into self-help groups, but do they get do they get to benefit from clinical psychologist services

**Respondent**

Yes, that was some two three four years ago we had BasicNeeds in collaboration with CEDEP that were bringing in eeh psychiatrics to come and review virtually all the clients that I've seen to and then eh eeh at one point we had some clinical psychologist also coming around but you know it's a program when time is up time is up everybody packs bag and baggage and leaves so that was then and eh from all evidence you could see that those were the times that most patients were gotten on our list like we were really able to reach out to most of them cos it's not really easy getting to the villages it's very difficult it's it's very difficult it's difficult hmm

**Interviewer**

Thank you sir, so how about human resources what mix of mental health professionals do you have

**Respondent**

Apart from the community psychiatric nurses with community mental health officers we don't have any other auxiliary staffs eerh so I have here eerh two community mental health officers and then five CPNs yeah

**Interviewer**

Okay, thank you very much how about eerh training and accreditation and licensing of mental health professionals working here at the community level how is that done

**Respondent**

Aaah currently I think eerh they're now training direct community health nurses but previously it's the registered mental nurses that after eerh going through let's say some three four years in the ward can choose to come to the community then go through what we call orientation for six months before they're brought to the community but now they're training nurses for community mental health jobs currently yeah

**Interviewer**

Mmm Yeah, and eerh the mental health profession as at the sub district level or community level how're they how're they managing who manages them

**Respondent.**

Hah, (silent) I play the role as the general overseer but eeh because we don't have enough.

**Interviewer**

(Interjects) Because you're the district coordinator

**Respondent**

Yes because I'm the district coordinator, but because we don't have enough we can't really get them all in the sub district ahaa so they are all stationed that is the difficult part they're all stationed in here but because I have divided the zones so you're in charge so I expect that you get your work done and bring me report then I follow up too and check whatever I expect you to do you do yes that is how it works but...

**Interviewer**

Thank you sir, yeah you were talking

**Respondent**

Eeh because we don't also have accommodation options available so you can't ask them to go there when the person has no accommodation so they'll have to leave from here to that place every now and then .

**Interviewer**

Mmhmm Okay, I guess you have heard about and probably worked with the mental health law from your perspective does the mental health law as it is promote eeeh provision of mental health services at the community level if so how?

**Respondent**

Huh I don't well it's it's there I don't see it promoting I don't see it promoting because you might have you might pass a law but if the tools and the materials to work with are not available how do you do it so the law will be there but nothing goes on I don't see it promoting

**Interviewer**

Mmhmm and do we have a mental health policy

**Respondent**

>Chuckles< personally sitting here I have heard but I don't know about it I don't know that much about it must be frank

**Interviewer**

Mmhmm let me step back and ask a question I need to ask on the human resources the mental health professionals in the ideal situation what mix if you had all the mental health professionals that should be available what should be the mix of the mental health professionals at each level sub district district regional and tertiary level mental health professionals at the sub district and district levels

**Respondent**

Sub district we have community mental health officers. At the district the CPNs are are there. At least there is a CMHO in the majority of districts of Ghana but not below. The challenge is the tools and resources for them to work with. I mean funds, logistics like motorbikes, proper offices, and furniture. If these are provided, we can go to the sub-districts and into the hinterlands to provide services...

**Interviewer**

(Interjects) if you have all that you need all the required professionals available what should be the mix of staffing?

**Respondent**

Community mental health officers’ community psychiatric nurses clinical psychologist and a psychiatrist

**Interviewer**

For what level

**Respondent**

Eh it's for the psychiatrist even in the district it would have been better

**Interviewer**

And the sub district?

**Respondent**

Sub district if you have the community psychiatric nurses there it's good in combination with the community mental health officers it's good if at the district level too we have a psychologist perfect even if we don't have a psychiatrist in the district and we have a clinical psychiatric officer it would have been good

**Interviewer**

So eerh again just as I had asked about the eerh levels of treatment is there a document spelling out the mix of staff at each level that should be adhered to do you have a manual or...

**Respondent**

No we don't we don't

**Interviewer**

That's fine okay alright, so we were talking about the policy and you said you heard it exists but you don't know the mental do you see the mental health law promoting the human rights of people in mental conditions

**Respondent**

Huh personal opinion if they are doing it I'm not seeing it personally if they're doing it am not seeing it cos eerh I've really seen that much change in the lives of our patients at my level maybe in another level they're doing it but right here no am not feeling it am not seeing it

**Interviewer**

Okay and eerh again does it do you see in guaranteeing the participation of people with mental health conditions in their treatment

**Respondent**

A guarantee

**Interviewer**

Does it guarantee people with mental health conditions?

**Respondent**

No no it doesn't not at all

**Interviewer**

Why do you say so

**Respondent**

You see it's it is been the old story where eh because the law really does not give you what you really want to work with it's the same pattern where clients are either eh going to the prayer camps where you yourself have to follow up and go and catch up with the patient and eh probably speak to the prayer camp owner make the person understand but if the laws were really working aah it will difficult for you to even hold the patient there even because those who have been held there and we've sent reports and they're not ready to release patients and we've sent reports the tribunal that have been set they don't nobody even comes around to come and check so it's virtually like eh cos ninety erhmm so that's why I said

**Interviewer**

Thank you I want to touch on financing of mental health services at the district community level what are the sources finance funding for mental health care services at the district and sub district levels.

**Respondent**

The district because per the Act we're supposed to be autonomous but unfortunately, we're not we're still attached to Ghana health service somehow so we depend on them so if they give us small

**Interviewer**

(Interjects) When you say we who are they we?

**Respondent**

We as in mental health department and the mental health eh eh eh authority they're virtually dependent on them somehow cos we're using their facilities we're using their eh eh eh materials if I need syringes I'll have to go to my mother hospital which is with Ghana health service so if they don't have they don't give you have nothing to work with cos you don't get anything from mental health authority so finances we don't have any we don't have money coming from anywhere we don't if the hospital does not help us that's it we're done yeah.

You see, unless you are working in mental health, healthcare workers are not interested in mental health matters. Cos, there is fear and social stigma. It is only the CPNs [Community Psychiatric Nurses] and the CMHOs [Community Mental Health Officers] who do everything. A negligible number are really interested and involved.

**Interviewer**

Okay so eh do people pay for the services provided at the community level

**Respondent**

>Exhales with a sigh< payment if they'll pay that is when they're buying thier own drugs but as in the service we all know it's supposed to be free but it's not that free honestly it's not that free so eehm the client has insurance obviously the client will be seen to will go through the system that is the free that is for consultation and stuff but for you to get your medication it is not free you'll have to buy

**Interviewer**

Okay so you've already talked of the fact that to some extend national health insurance is working and covering consultation your card does it also pay for anything else the national health insurance

**Respondent**

One medication that I'm aware of that they have been paying for but it is on and off that is tablet carbamazepine or Tegretol so far that is the only the only drug that is on the national health insurance scheme that our patients here claim and because most of my clients here majority are epileptic patients so somehow it's it helps them a little it helps them but where insurance does not also pay the pharmacy shop that is where we have difficulties because they'll not bring stocks cos they've not had their money then the patient will have to come back and start buying again that's what happens

**Interviewer**

Eei, so when you say it's not entirely free what apart from medicines what aspects of payment do they also have to make

**Respondent**

Mmm if if if probably if we have to admit you at the ward due to one or two reasons you'll have to pay for it you'll have to pay even if you have insurance because you know most of our clients sometimes can be very destructive and taking care of them is not an easy job you'll do a down payment eh as eh an assurance so that is something that was instituted within our facility so you'll have to but if you're insured under the NHIS, it helps a little with your folder the other stuff too will be taken care of by the insurance but you'll have to pay something

**Interviewer**

Mmm, okay thank you I want to ask about procurement of medical products including medicines how are they planned for procured distributed for mental health services provision at the community level.

**Respondent**

Okay what we do here eh let me say we procure our medications from the regional medical stores and it is only when there's availability that your regional coordinator will call okay we're issuing this number of medications we're sharing it so go for yours that is how it is aha but if you if you the pharmacy the hospital pharmacy will go and then take it on behalf of the district then we come for it and distribute it to the various this thing but because sometimes we go on what we call aah routine eerh outreaches we send some of the medications there to see to them then we enter and record every medication that has been disbursed yeah

**Moderator**

So eh am interested I don't want to forget how many sub districts facilities do you have ( music plays in background) that render mental health services

**Participant**

Here we have four mmm we have Hwedeme Obonsu

**Interviewer**

Mmhmm yeah go on just go on

**Respondent**

Eeh Atwereboana and then Akutreso these are the places that we have

**Interviewer**

Hwedeme is spelt how do you spell Obonwo

**Respondent**

Obonsu o-b-o-n-s-u

**Interviewer**

Okay and Hwedeme which one else

**Respondent**

Obonsu Atwereboana

**Interviewer**

Atwereboana how is it spelt

**Respondent**

A-t-w-e-r-e-b-o-a-boana yeah

**Interviewer**

The last one

**Respondent**

Akutreso a-k-u-t-r-e-s-o

**Interviewer**

Out of how many subdistricts

**Respondent**

Out of five

**Interviewer**

Okay and they're each staffed with a CPN or community psychiatric officer community mental health officer.

**Respondent**

No. They're individually community like community nurses that we train and focal persons like community volunteers and train them purposely for that though they're community nurses within the this thing but we take them on training so they can function there and refer some of the cases to us alongside with the volunteers (noise in background) we're not enough to really get ourselves the the even here sometimes it's difficult handling one patient

**Interviewer**

(Interjects) so how many mental health professionals do you have in the district

**Respondent**

Currently five yes

**Interviewer**

Hah and they're made up of how many CPNs

**Respondent**

CPNs five

**Interviewer**

Oh all of you are CPNs so you have not had the benefit of

**Respondent**

Two community mental health officers two

**Interviewer**

That's seven

**Respondent**

So seven two community health officers

**Interviewer**

Okay okay

**Respondent**

Oh I think we're even short of one so we're now four one left for a transfer this year so we're four four CPNs so sometimes I don't even count myself cos since am at the district level at the same time here

**Interviewer**

So it's three okay that's fine and eeh so we're at the point of you you have never or do you plan for the year quantity of medications you want and other medical products you require you want procured for you

**Respondent**

Yes yes every year

**Interviewer**

So how does it how does it go where if you prepare where does it go and how does it end do you get it

**Respondent**

Yeah so with our we have something we call annual performance review and in that report we put all those things in there and within the hospital facility too we have eeh quarterly performance review where you'll also have to state the quantity and stuff that you want but you know we don't get anything so you keep on writing and writing

**Interviewer**

>Laughs< very interesting okay so I want to move on to information flow and feed back processes eh I know you collect a lot of or some amount of data eh on people using mental health services at the or what kinda of data is it that you collect

**Respondent**

Here it's more of eh if you have any diseased patient if you've defaulters the types of mental health conditions the types the number of patients in each category if we have eh relapse cases if there's been any eh like I did mention them eh what again it's a whole lot oo (chuckles) it's a whole lot yeah .

**Interviewer**

Okay alright but these are the common ones the ones that stand out

**Respondent**

Yeah they are the common ones

**Interviewer**

So the DHIMS you've heard of the district information management systems is the eeh mental health unit providing any information through the DHIMS

**Respondent**

Yes every month I need to enter my reports in the DHIMS

**Interviewer**

And what does it capture what does the DHIMS capture

**Respondent**

Virtually all that I mentioned eeh the number of cases we've seen to within that month eeh specifically if schizophrenia the number if bipolar the number if epilepsy the number and then defaulters how many eeh home visits we've done how many school health talks we've done church health talks we've done community eeh community ehh what we call is it the ones that the community comes in to assess eeh our performance we we like it's a durbar you give them the chance to ask their questions and see whatever they want to see those are some of the things that we report on and the self-help groups number of times we've had meetings and stuffs

**Interviewer**

Mhmm so can you describe the feedback process you have

**Respondent**

When we have the community meeting everything everything always boil down to the fact that they find it difficult to get their medication that has been the challenge

**Interviewer**

What kind of people come for these durbars

**Respondent**

Oh assemblymen clients the clients themselves and the caregivers

**Interviewer**

Are they regular?

**Respondent**

In fact sometimes we hold it once every eeeh half years it's not eh regular cos it takes time to plan that thing thirty people transported to the venue is no joke

**Interviewer**

And and do you do you get eh service users or caregivers or community members come with feedback or suggestions as to how you can improve your services to them solicited or not

**Respondent**

Unless under those durbars that you get the chance but one caregiver had suggested to us to create a suggestion box where they can input yeah and unfortunately most of the natives here are not well educated as you can when you went to the interview you can just see so eh really putting things on paper becomes a problem ehmm so unless the gathering that they can speak their mind apart from that we don't have any other means we use to

**Interviewer**

And do you always find them constructive or

**Respondent**

Ooh yes yes I've I personally have issues with customer care it's one of my eh my concern my major concern I I I don't really joke with my patients so when I come and my patient reports to me that he or she has not been treated well it it becomes a problem for me ahaa apart from that and that had been a major problem with medication issue I came and I went there and I didn't even get the medicine to buy those stuff it becomes a problem so they genuinely they take some of the things they're angry about they have the right because it's true it's true that they're having difficulties

**Interviewer**

Do you have any research going on eeh around mental health and eh mental health services provision at the community level

**Respondent**

Yes and no yes just recently Daniel came in and he started something but previously I've always asked that we need people to come in to come and do and I've spoken to a few people about our conditions here especially with the epileptic cases how come we have that high number but we're yet to see someone coming in to come and do that research but Daniel came in to eh work on one part and we're yet to see what we can get

**Interviewer**

Thank you so much eh I want to I've got some issues of leadership and governance of mental health at the community level what can you describe the leadership around mental health especially at the district sub district and lower levels

**Respondent**

As in eeh leadership from with mental health because like I rightly said because we don't have more of the CPNs in the subdistrict and eeh leasing with the eeh volunteers and other providers like mental health providers that we've trained is not difficult yes it's not difficult they easily give out information that they need to give cos that's one of our clients he wanted to come and check up on me yeah so they eeh easily like eh yeah give out information that needs to be given the the information flow is easy just that eh at my end where I also need information from the top but it's difficult to get it yeah

**Interviewer**

So how what how do you see the leadership of the health authorities when it comes to mental health they show leadership do they see do you see a sense of

**Respondent**

No I must be very frank maybe they do it in Accra but in and around the regions no no no look if for a whole year your your regional coordinator comes in just once because she doesn't have the means to come it tells you it's a problem it's a problem cos we all work under supervision it's always good to come and see firsthand information on what come and see how how your staffs are fairing cos [because] it's not easy you're it's like you're left alone to struggle for yourself and it's difficult if you're not that proactive to get individual financers involved to come in and help it means you're you're down I had to lease with a pharmacy shop the only pharmacy shop in this town to get some of the medications some of the psychotropics and anticonvulsants here because they for what reason should they bring those medications I had to lease it's it's not easy it's difficult so it's like you've been left alone emm you've been left to hustle

**Interviewer**

How about politically the political authorities is there any sense of interest and commitment to see development of mental health especially at the community level

**Participants**

Zero zero in the sense that when you even want to really go for a percentage of the common wealth fund that comes to the district that is meant for the care for the upkeep of the patient it's even a problem when we have eh eh patients who are the destitute ones who are roaming about who can be taken care of by the district and nobody seems to care uh-huh politically it's even weaker than anything their interest is not there nobody cares and am being frank you'll go there several times talk to them about some of the things you think you can do to improve mental health in the in the community and nobody cares nobody cares

**Interviewer**

So eh this issue of mental health authority and Ghana health service you know there was earlier on you were saying we and them and I said oh who are they how is it working the mental health authority Ghana health service and health professionals like you providing mental health services at the district and lower levels how are you managing it

**Respondent**

It is becoming even difficult than it used to be especially when they declared that eh eh what do I call it autonomous position aah some of them you're on you own so what do you want from us why would you need this from us so it becomes very difficult let me take a let me give you an example funds come and it's supposed to be managed by maybe the regional coordinator he has to go to the regional director of Ghana health service he'll determine where the amount that should be given to you so where lyes the autonomy so you're virtually like you're still being manipulated you're still being told what to do you draw eh eh CPNs wants to go to school they at the regional level have decided that this the kind of program that we want for our district for our staff and there's nothing like go and do Psychology anything that's in relation to mental so what are you doing so virtually the CPNs are running to general staff because they'll go and do ENT eye maybe PA and will leave psychiatry so right now the staffs are rather running away instead of staying so what good is it it means they're not on their feet still what if they were they'll they're the ones who'll manage our affairs and our human aspect the human resource aspect so if I need to go to school am going through them they need to approve me not Ghana health service so we're stack we can't go forward we don't know what to do so it's worse than before that's my personal opinion

**Interviewer**

So do you see yourself belonging to mental health authority or Ghana health service

**Respondent**

>Laughing< I'm here and there

**Interviewer**

What do you mean how's that

**Respondent**

I'm here because it is still me under Ghana health service am being paid not under mental health authority so sometimes when you're even filling a form you don't know where to put yourself >laughs<

**Interviewer**

What form is that?

**Respondent**

Maybe if you need to go to school or you need to there's a promotion and stuff that you need to fill out some details I mean you don't know where to put yourself you don't know whether you're a mental health authority or Ghana health service

**Interviewer**

I know you've talked about it and eeh and it's becoming protracted or there's some resolution is there some resolution of it that it's becoming eeh helpful or is it's just becoming more and more protracted

**Respondent**

To me it's becoming more and more protracted to me personally my opinion very protracted

**Interviewer**

Wow interesting okay so moving just to my very last questions and that is this issue about integration of mental health into primary care or community level or sub districts what is your understanding of it

**Respondent**

I only understand that we should treat all manner of patients in the primary setting equally where we don't say you people go there let's see to you here you people go there let's see to you here like the way we do with the HIV patients like we want to really get them all together in one fold so one aspect is when now we can easily admit a psychiatric patient in a general ward which is helping cos we don't want to really stigmatize or discriminate so it's it is helping initially it's difficult for you to admit but because now there's a bed allocated at least two so you have no choice than to accept me and sometimes it comes with difficulties but we still manage yeah we still get it done

**Interviewer**

What usually are the difficulties? <laughs>

**Respondent**

Oh but the patient is coming to spoil our things eh me I can't take care of this patient o am a general nurse I can't take care of this patient of you can't come and stay with the patient then please take your thing away your thing so it's my property oh your patient is in o come and see to your patient whereas when you call me I can see to a general patient at the same time but you've been trained to do both and you think it's only your own that you need to do it's kind of but the primarily they eh gradually it's picking up it is picking up we can bring them together maybe if they're going for aah for RCH we bring them together like if the RCH the reproductive child health promotion the stuff we go together with them for eh when they do eh together we also do our own give the talk and so gradually things are getting better there the primary level

**Interviewer**

So what can facilitate this better for it to?

**Participant**

More training for the auxiliary and the general nurses if you get to understand what it means to really get others eeh erh sectors integrated I mean the better it´s going to improve our services mmmh because if you can do it. I can do it, you can also see that patient and say this is for these people emm it is not that way ,intension training for them, staffs and in-service personnel

**Interviewer**

What other opportunities exist?

**Respondent**

As in mental health?

**Interviewer**

Eemmmm

**Respondent**

Laughing …we are still worry

**Interviewer**

For integration

**Respondent**

For integration……eerh if they real help the CPNs to get themselves educated more into this kind of psychotherapy, psychology etc those petty, petty things that help improve the quality of life of our patients I think it will along to help the primary sector.

**Interviewer**

Okay, thank you we are almost ended and the so what potential challenges are we likely to encounter in our request to help mental health better integrated?

**Respondent**

You have difficulties because the shareholders that will be involved know that mental health is a spending venture you don’t profit and that alone is a challenge they expending officers they don’t generate funds so is going to be one of the number challenge… yeah

**Interviewer**

Eemm okay, let’s go…ok we had have a good conversation are they others recommendations ...(a phone ringing) that you want to make from the discussion that you have thought I would have asked but I have not aske and you think it is important to share with us?

**Respondent**

They…I think mental health authority will have to wake up and empower the district mental coordinators because it is only when they come low to the grounds because we deal with those in the grounds it is only we focus on the primary aspect of it that would get the result at the top and only by empowering those sectors because if I don’t have the means how do I help our patients? I will have to sit here every day and wait for them but the real job is at the community not in the offices if the things I need to work with are not there, the motor bike, maybe the vehicle, the logistics are not here how do I work ? is rather frustrating for me and I then sit back every individual get tired at one point in time when you there is no help coming. They should come and empower us and they see things change, they will not….they will real change ..Some of us are willing to work seriously but the means to like like I..i.. look at how my car is? And that is what I use to do all the rounds not even fuel allowance and the small one they called me on have been sat ion it…I will not come

**Interviewer**

From the GHS or the MHS?

**Respondent**

From the GHS so

**Interviewer**

Because they don’t that you don’t deserve it ?

**Respondent**

They think that I don’t deserve it

**Interviewer**

or it is because the issue that you are not part of them?

**Respondent**

Personally, I think that’s it because we don’t generate funds for them.so we are spending office, we don’t generate anything for them so that’s it unless they help to reduce something ….is going to be difficult very, very difficult I have the means but how to get there is the problem ..is quiet difficult.

**Interviewer**

I see…..interesting, interesting……thank you very much.

**Respondent**

You are welcome my boss

**Interviewer**

Do you have any question for me?

**Respondent**

As far as questions I have plenty for you, but, I will not ask them now.

**Interviewer**

Laughing……….

**Respondent**

So I will not ask you now…eemm I will not ask you now.

**Interviewer**

Laughing….. Thank you very much.

1. Plaza is an open ground in the Bolgatanga township where gatherings are held. There is a clinic close by Plaza and sometimes psychiatrist coming on treatment outreach facilitated by BasicNeeds-Ghana consult from there. The space allow for many people to converge for the medical [mental health] treatment services provided
