# Supplementary material for: Leadership and governance for integrating mental healthcare at the primary healthcare (PHC) level: A mixed methods study in Ghana
Source: PLOS Glob Public Health. 2025 Aug 19;5(8):e0002672. doi: 10.1371/journal.pgph.0002672 (PMC12364371; doi:10.1371/journal.pgph.0002672)
Supplement: S1 Checklist — (DOCX) [file pgph.0002672.s003.docx]

| **Section and Topic** | **Item #** | **Checklist item** | **Location where item is reported** |
| --- | --- | --- | --- |
| **TITLE** | | |  |
| Title | 1 | Identify the report as a systematic review. | Not a systematic review.  Manuscript Page 1; Lines 3-4 |
| **ABSTRACT** | | |  |
| Abstract | 2 | See the PRISMA 2020 for Abstracts checklist. | Manuscript Page 2; Lines 20-40 |
| **INTRODUCTION** | | |  |
| Rationale | 3 | Describe the rationale for the review in the context of existing knowledge. | Manuscript Pages 3-5;  Lines 20-117 |
| Objectives | 4 | Provide an explicit statement of the objective(s) or question(s) the review addresses. | Manuscript Page 5; Lines 119-123 |
| **METHODS** | | |  |
| Eligibility criteria | 5 | Specify the inclusion and exclusion criteria for the review and how studies were grouped for the syntheses. | Manuscript Page 7; Lines 163-197 |
| Information sources | 6 | Specify all databases, registers, websites, organisations, reference lists and other sources searched or consulted to identify studies. Specify the date when each source was last searched or consulted. | PubMed, Google Scholar, University of Ghana online library, grey literature (WHO, Ghana Health Service, Ghana Mental Health Authority, BasicNeeds-Ghana). 2022 was the last searched time.  Manuscript’s list of references/ Bibliography.  Manuscript’s Pages 35-40; Lines 765-1002 |
| Search strategy | 7 | Present the full search strategies for all databases, registers and websites, including any filters and limits used. | Not Applicable |
| Selection process | 8 | Specify the methods used to decide whether a study met the inclusion criteria of the review, including how many reviewers screened each record and each report retrieved, whether they worked independently, and if applicable, details of automation tools used in the process. | Not Applicable  Manuscript Page 6; Lines 135-146, 162-197 |
| Data collection process | 9 | Specify the methods used to collect data from reports, including how many reviewers collected data from each report, whether they worked independently, any processes for obtaining or confirming data from study investigators, and if applicable, details of automation tools used in the process. | Manuscript Pages 9-11; Lines 222-284 |
| Data items | 10a | List and define all outcomes for which data were sought. Specify whether all results that were compatible with each outcome domain in each study were sought (e.g. for all measures, time points, analyses), and if not, the methods used to decide which results to collect. | Manuscript Pages 10-12; Lines 242-293 |
|  | 10b | List and define all other variables for which data were sought (e.g. participant and intervention characteristics, funding sources). Describe any assumptions made about any missing or unclear information. | Not applicable |
| Study risk of bias assessment | 11 | Specify the methods used to assess risk of bias in the included studies, including details of the tool(s) used, how many reviewers assessed each study and whether they worked independently, and if applicable, details of automation tools used in the process. | Manuscript Page 12; Lines 285-293 |
| Effect measures | 12 | Specify for each outcome the effect measure(s) (e.g. risk ratio, mean difference) used in the synthesis or presentation of results. | Manuscript Page 13; Lines 297-305 |
| Synthesis methods | 13a | Describe the processes used to decide which studies were eligible for each synthesis (e.g. tabulating the study intervention characteristics and comparing against the planned groups for each synthesis (item #5)). | Not Applicable |
|  | 13b | Describe any methods required to prepare the data for presentation or synthesis, such as handling of missing summary statistics, or data conversions. | Manuscript Page 13; Lines 305-315 |
|  | 13c | Describe any methods used to tabulate or visually display results of individual studies and syntheses. | Manuscript Page 13-14; Lines 319-328 |
|  | 13d | Describe any methods used to synthesize results and provide a rationale for the choice(s). If meta-analysis was performed, describe the model(s), method(s) to identify the presence and extent of statistical heterogeneity, and software package(s) used. | Not Applicable |
|  | 13e | Describe any methods used to explore possible causes of heterogeneity among study results (e.g. subgroup analysis, meta-regression). | Not Applicable |
|  | 13f | Describe any sensitivity analyses conducted to assess robustness of the synthesized results. | Not Applicable |
| Reporting bias assessment | 14 | Describe any methods used to assess risk of bias due to missing results in a synthesis (arising from reporting biases). | Manuscript Page 12; Lines 288-293 |
| Certainty assessment | 15 | Describe any methods used to assess certainty (or confidence) in the body of evidence for an outcome. | Not Applicable |
| **RESULTS** | | |  |
| Study selection | 16a | Describe the results of the search and selection process, from the number of records identified in the search to the number of studies included in the review, ideally using a flow diagram. | Not Applicable |
|  | 16b | Cite studies that might appear to meet the inclusion criteria, but which were excluded, and explain why they were excluded. | Not Applicable |
| Study characteristics | 17 | Cite each included study and present its characteristics. | Not Applicable |
| Risk of bias in studies | 18 | Present assessments of risk of bias for each included study. | Not Applicable |
| Results of individual studies | 19 | For all outcomes, present, for each study: (a) summary statistics for each group (where appropriate) and (b) an effect estimate and its precision (e.g. confidence/credible interval), ideally using structured tables or plots. | Manuscript Pages 14-28; Lines 329 - 643 |
| Results of syntheses | 20a | For each synthesis, briefly summarise the characteristics and risk of bias among contributing studies. | Not Applicable |
|  | 20b | Present results of all statistical syntheses conducted. If meta-analysis was done, present for each the summary estimate and its precision (e.g. confidence/credible interval) and measures of statistical heterogeneity. If comparing groups, describe the direction of the effect. | Not Applicable |
|  | 20c | Present results of all investigations of possible causes of heterogeneity among study results. | Not Applicable |
|  | 20d | Present results of all sensitivity analyses conducted to assess the robustness of the synthesized results. | Not Applicable |
| Reporting biases | 21 | Present assessments of risk of bias due to missing results (arising from reporting biases) for each synthesis assessed. | Not Applicable |
| Certainty of evidence | 22 | Present assessments of certainty (or confidence) in the body of evidence for each outcome assessed. | Manuscript Page 28; Lines 639-6433 |
| **DISCUSSION** | | |  |
| Discussion | 23a | Provide a general interpretation of the results in the context of other evidence. | Manuscript Pages 29-30; Lines 644-698 |
|  | 23b | Discuss any limitations of the evidence included in the review. | Manuscript Page 30-31; Lines 700-723 |
|  | 23c | Discuss any limitations of the review processes used. | Manuscript Page 30-31; Lines 700-723 |
|  | 23d | Discuss implications of the results for practice, policy, and future research. | Manuscript Page 32; Lines 720-723 |
| **OTHER INFORMATION** | | |  |
| Registration and protocol | 24a | Provide registration information for the review, including register name and registration number, or state that the review was not registered. |  |
|  | 24b | Indicate where the review protocol can be accessed, or state that a protocol was not prepared. | The study protocol is available on request |
|  | 24c | Describe and explain any amendments to information provided at registration or in the protocol. | Interview questionnaire, discussion guides were revised to provide clarity on the issues explored. |
| Support | 25 | Describe sources of financial or non-financial support for the review, and the role of the funders or sponsors in the review. | None. However, the lead author is a paid employee of BasicNeeds-Ghana |
| Competing interests | 26 | Declare any competing interests of review authors. | None |
| Availability of data, code and other materials | 27 | Report which of the following are publicly available and where they can be found: template data collection forms; data extracted from included studies; data used for all analyses; analytic code; any other materials used in the review. | Available as Supplementary Information of the manuscript |

*From:*  Page MJ, McKenzie JE, Bossuyt PM, Boutron I, Hoffmann TC, Mulrow CD, et al. The PRISMA 2020 statement: an updated guideline for reporting systematic reviews. BMJ 2021;372:n71. doi: 10.1136/bmj.n71. This work is licensed under CC BY 4.0. To view a copy of this license, visit <https://creativecommons.org/licenses/by/4.0/>
