# Supplementary material for: Leadership and governance for integrating mental healthcare at the primary healthcare (PHC) level: A mixed methods study in Ghana
Source: PLOS Glob Public Health. 2025 Aug 19;5(8):e0002672. doi: 10.1371/journal.pgph.0002672 (PMC12364371; doi:10.1371/journal.pgph.0002672)
Supplement: Attachment — Submitted filename: Review-PGPH-D-23-02157.pdf [file pgph.0002672.s004.pdf]

December 24, 2023

Dear Editor and Authors,

I reviewed the article “Leadership and governance of mental healthcare and integration at the community level: a mixed methods study in Ghana.” I would like to express my appreciation for your valuable contribution to the governance field.

However, as I reflected on your article, I believe that enhancing the scientific rigor, especially in the qualitative aspect, could further strengthen your findings. I would like to offer some suggestions for consideration:

Even though the article adeptly outlines the research design and methods, it is crucial to consider certain refinements to ensure methodological rigor. Specifically, in the context of assessing leadership and governance in mental healthcare, a more detailed explanation of the mapping of actors would enhance the clarity and robustness of the study.

Further to strengthen the research design, it is recommended to explicitly mention the process of actor mapping, including the identification of relevant stakeholders and the definition of selection criteria. This will provide readers with a clear understanding of how the study identified and engaged with key actors in mental healthcare at the community level.

Authors should provide more information about how themes or patterns were identified in your qualitative data. This could involve a discussion on the coding process, inter-rater reliability, or any measures taken to ensure the trustworthiness of your qualitative findings.

In reviewing the qualitative methodology in the manuscript, it became apparent that there are some technical discrepancies, particularly regarding participant selection. In qualitative research, participant selection is typically guided by specific criteria rather than random sampling.

I recommend explicitly stating the criteria employed in participant selection, such as specific roles, experiences, or characteristics that make individuals eligible for inclusion in the study. This information is crucial for readers to understand the rationale behind participant selection and ensures that the qualitative data collection aligns with the research objectives.

"Theoretical Saturation" is a concept mainly associated with the qualitative research methodology called "Grounded Theory". How did you adapt it to your method used in your analysis?

Other points to strengthen in your manuscript are the following:

To contextualize the integration of mental health within the broader healthcare landscape, it is essential to draw an outline of the structure of health services in Ghana.

When examining mental health resources and services, it is crucial to utilize a standardized metric for comparison. In this context, I recommend employing the number of resources per 100 thousand inhabitants as a key indicator. This metric allows for a more equitable and insightful assessment of mental health support across different regions or populations. (Lines 94-102 in their manuscript).

Moreover, it would be beneficial to include a brief section discussing the implications of the findings and specific recommendations derived from the study. Also include a section on study limitations and future directions for a more comprehensive discussion.

Finally, when reviewing the manuscript, I observed that two different citation styles are employed, leading to inconsistencies in the presentation of references. Maintaining a uniform citation style is crucial for the overall coherence and professionalism of the article.
