# Supplementary material for: Leadership and governance for integrating mental healthcare at the primary healthcare (PHC) level: A mixed methods study in Ghana
Source: PLOS Glob Public Health. 2025 Aug 19;5(8):e0002672. doi: 10.1371/journal.pgph.0002672 (PMC12364371; doi:10.1371/journal.pgph.0002672)
Supplement: Attachment — Submitted filename: Letter_YARO_Response to Reviewers_05022024.pdf [file pgph.0002672.s006.pdf]

C/O Department of Health Policy, Planning and Management
School of Public Health, College of Health Sciences
University of Ghana, P. O. Box LG13, Legon
Accra, Ghana

Thursday, 25th January 2024

Jennifer Tucker, PhD
Staff Editor
PLOS Global Public Health

Dear Dr Jennifer TUCKER,

**ADDRESSED REVIEW COMMENTS**

Thank you for the constructive feedback provided on the manuscript entitled "Leadership and governance of mental healthcare and integration at the community level: a mixed methods study in Ghana".

Below are comments made by reviewers, as well as point-by-point responses indicating how and where these have been addressed throughout the manuscript.

| S/N | REVIEW COMMENT | RESPONSES |
|---|---|---|
| **A.** | **Editor/Reviewers Comments** | |
| | **Editorial Comments** | |
| 1. | Please include a complete copy of PLOS' questionnaire on inclusivity in global research in your revised manuscript. | This has been done and uploaded as supplementary file |
| 2. | Please describe in your methods section how capacity to provide consent was determined for the participants in this study. Please also state whether your ethics committee or IRB approved this consent procedure. If you did not assess capacity to consent, please briefly outline why this was not necessary in this case. | This has been addressed. Kindly see page 7-10 lines 176-186; 217-224; 231-241. |
| 3. | . Please provide separate figure files in .tif or .eps format only and remove any figures embedded in your manuscript file. Please also ensure all files are under our size limit of 10MB | The figure has been concerted to tif and uploaded separately |
| 4. | . We have noticed that you have a list of Supporting Information legends in your manuscript. However, there are no corresponding files uploaded to the submission. Please upload them as separate files with the item type 'Supporting Information'. | All supporting file referenced in manuscript (Page 37; Line 887) have been uploaded |
| 5. | Fig 1: please (a) provide a direct link to the base layer of the map (i.e., the country or region border shape) and ensure this is also included in the figure legend; and (b) provide a link to the terms of use / license information for the base layer image or shapefile. We cannot publish proprietary or copyrighted maps (e.g. Google Maps, Mapquest) and the terms of use for | The figure was composed by Gerald Yiran, Department of Geography and Resource Development, University of Ghana, Legon; Email: |

| | | |
|---|---|---|
| | your map base layer must be compatible with our CC-BY 4.0 license.<br><br>Note: if you created the map in a software program like R or ArcGIS, please locate and indicate the source of the basemap shapefile onto which data has been plotted. | gloriasheilayiran@gmail.com; Mobile phone: +233244847018. This has been acknowledged |
| | **Reviewers** | |
| 6. | Even though the article adeptly outlines the research design and methods, it is crucial to consider certain refinements to ensure methodological rigor. Specifically, in the context of assessing leadership and governance in mental healthcare, a more detailed explanation of the mapping of actors would enhance the clarity and robustness of the study.<br><br>Further to strengthen the research design, it is recommended to explicitly mention the process of actor mapping, including the identification of relevant stakeholders and the definition of selection criteria. This will provide readers with a clear understanding of how the study identified and engaged with key actors in mental healthcare at the community level. | These additions have been done; Kindly see sub-heading on sampling. Page 8; Lines 179-186 of manuscript with tracked changes |
| 7. | Authors should provide more information about how themes or patterns were identified in your qualitative data. This could involve a discussion on the coding process, inter-rater reliability, or any measures taken to ensure the trustworthiness of your qualitative findings | More details have been provided on pages 9 and 10 (Lines 217-224) of the revised manuscript with tracked changes. |
| 8. | In reviewing the qualitative methodology in the manuscript, it became apparent that there are some technical discrepancies, particularly regarding participant selection. In qualitative research, participant selection is typically guided by specific criteria rather than random sampling.<br><br>I recommend explicitly stating the criteria employed in participant selection, such as specific roles, experiences, or characteristics that make individuals eligible for inclusion in the study. This information is crucial for readers to understand the rationale behind participant selection and ensures that the qualitative data collection aligns with the research objectives. | These additions have been done; Kindly see sub-heading on sampling. Page 7-8; Lines 176-187 |
| 9. | "Theoretical Saturation" is a concept mainly associated with the qualitative research methodology called "Grounded Theory". How did you adapt it to your method used in your analysis? | This has been clarified on page 9 and 10 lines 209-224 |
| 10. | To contextualize the integration of mental health within the broader healthcare landscape, it is essential to draw an outline of the structure of health services in Ghana. | The five-tier system(s) of the health service in Ghana has been added. See page 6; Lines 141 – 148. |
| 11. | When examining mental health resources and services, it is crucial to utilize a standardized metric for comparison. In this context, I recommend employing the number of resources per 100 thousand inhabitants as a key indicator. This metric | Thank you for the comments. This has been highlighted in the introduction. The purpose of the study was not to measure metrics |

| | | |
|---|---|---|
| | allows for a more equitable and insightful assessment of mental health support across different regions or populations. (Lines 94-102 in their manuscript). | but examine and describe the governance and leadership situation with respect to mental health services in Ghana |
| 12. | Moreover, it would be beneficial to include a brief section discussing the implications of the findings and specific recommendations derived from the study. Also include a section on study limitations and future directions for a more comprehensive discussion. | Revision has been made and limitations and future directions added. Kindly see pages 26-27 |
| 13. | Finally, when reviewing the manuscript, I observed that two different citation styles are employed, leading to inconsistencies in the presentation of references. Maintaining a uniform citation style is crucial for the overall coherence and professionalism of the article. | The citations have been revised for consistency |

Thanks again for your kind review.

Yours Sincerely,

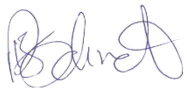

Peter Badimak YARO

E-mail: peter.yaro@basicneedsghana.org

Mobile Tel.: +233(0)244572733
