# Supplementary material for: Leadership and governance for integrating mental healthcare at the primary healthcare (PHC) level: A mixed methods study in Ghana
Source: PLOS Glob Public Health. 2025 Aug 19;5(8):e0002672. doi: 10.1371/journal.pgph.0002672 (PMC12364371; doi:10.1371/journal.pgph.0002672)
Supplement: Attachment — Submitted filename: YARO_Letter_response to Reviewer comments_YARO.27012025.pdf [file pgph.0002672.s007.pdf]

Editors-in-Chief
PLOS Global Public Health
Journal on Health Policy and Systems Research

Dear Editors-in-Chief,

Resubmission of Revised Manuscript: Leadership and governance of mental healthcare and integration at the community level: a mixed methods study in Ghana(PGPH-D-23-02157R1)

I wish to resubmit the revised version of the above manuscript for review and consideration for publication in the PLOS Global Health Journal. The authors have addressed all the comments and provide a point-by-point response as follows:

Reviewer 1
Comment 1: Title: While leadership and governance of mental healthcare are relevant across various levels, from global to local contexts, the current manuscript focuses specifically on leadership and governance related to integrating mental healthcare into primary healthcare (PHC). This specificity should be clearly reflected in the title to enhance clarity for readers and avoid any ambiguity.

To the best of my understanding, the current manuscript focuses on "leadership and governance for integrating mental healthcare at the community level"

Response 1: The authors agree with the reviewer and has changed the title of the manuscripts from 'Leadership and governance of mental healthcare and integration at the community level: mixed methods study in Ghana' to 'Leadership and governance for integrating mental healthcare at the primary healthcare (PHC) level: a mixed methods study in Ghana'

Comment 2: Introduction: While the introduction seeks to emphasize the scope and importance of the problem under study and identify existing gaps, the arguments and claims should be substantiated with contemporary and specific evidence. For instance, arguments claimed from "WHO's Integrating Mental Health in Primary Health Care Package (https://www.emro.who.int/mnh/publications/integrating-mental-health-in-primary-health-care-package.html) would provide more relevant and specific context for mental healthcare compared to those references on generic health care context.

Additionally, the authors should verify the accuracy of references cited throughout the manuscript. For example, references 10 and 13 appear to be identical and need correction.

Response 2: The URL - https://www.emro.who.int/mnh/publications/integrating-mental-health-in-primary-health-care-package.html - reference on 'WHO's Integrating Mental Health in Primary Health Care Package' provided for more relevant and specific context is noted and the references of the manuscript have been dully revised.

Please, see manuscript lines 45, 47, & 50.

References of the manuscript have been checked and verified. References 10 (now 13) and 13 (now 15) are different documents. Please, see manuscript lines 555-558, 559-561, and 572-575.

Comment 3: The sentence in lines 116-117 needs to be completed to reflect the context under enquiry. "Improving and sustaining community mental health is integral to overall **health system strengthening efforts ………………..."**

Response 3: The sentence in lines 116-117 has been reviewed and is a fully complete as follows, 'Improving and sustaining community mental is integral to overall health and system strengthening efforts' and needs no revision. Please, see lines 115-116 of the revised manuscript.

Comment 4: The authors should reevaluate the use of the term "indicators" in line 119, considering the methodology employed and the subject matter explored.

Response 4: The term 'indicators' in manuscript has been replaced with 'undertakings' as in manuscript line 118.

Comment 5: Objectives: The authors should ensure coherence between the title, abstract, and stated objectives. In the aim, the phrase "among others" appears to dilute the focus, making the primary topic of "Leadership and Governance," on which the case is built, seem secondary. Moreover, "oversight, coordination, and general stewardship of mental healthcare at the PHC" is distinct from "integration of mental healthcare at the PHC." The authors should clarify and align these concepts for better consistency.

Additionally, the authors might consider using a more precise and technically appropriate term for "oversight" that complements better with the term "coordination."

Response 5: Objectives:

The phrase 'among others' has been deleted.

The aim has been revised as '… to assess the leadership and governance in place for the integration of mental healthcare at the PHC in Ghana. See manuscript lines 120-121.

Use of the terms in the manuscript have been revised.

Comment 6: Materials and methods: The authors should clearly define the primary purpose of both the qualitative and quantitative approaches to enhance clarity.

Response 6: Materials and methods:

This has been revised with additional text which reads as 'The primary purpose for the qualitative and quantitative approaches are to maximise the effect of both methods in proffering a better understanding of the phenomenon being studied than will be the case with

just a singular use of one of either approaches hence minimising the bias and weaknesses of qualitative or quantitative research.’ Please, see manuscript lines 126-130.

Comment 7: In the section on the study area, the text refers to Figure 1, while the figure title labels it as Figure 2.1. Additionally, the map does not display the three ecological zones. The authors should clarify the relevance of mentioning the three ecological zones and their connection to the context of the study, if applicable.

Response 7: The Figure refers to Figure 1 in the manuscript, and the label of Figure 2.1 is inadvertent. The map depicts the actual study locations, which by their location are coterminous with the ecological zones of Ghana.

Comment 8: In Line 139, do “Sub-district” level health facilities provide primary and secondary health care services?

Response 8: The sentence has been revised for clarity, (Please, see revised manuscript Lines 141-143). The sentence covers the gamut of services the regional, district, and sub-district healthcare facilities provide.

Comment 9: Sample Size: The authors should provide better clarity on sample size estimation. Typically, the size of the population is not directly relevant for sample size calculation, except in cases where finite population correction is applied.

Additionally, it is unclear how the sample size calculation, based on a 13% prevalence of mental health conditions, is applicable to other stakeholders such as health policy officials, healthcare managers, service providers, primary caregivers of individuals with mental health conditions, civil society organizations, self-advocates, and mental health champions.

Further, there is a lack of clarity regarding the target districts, how the population of 956,597 scales to 3,095,520 with a 13% prevalence, and how the final sample size of 1,010 was determined (the calculated sample size based on the parameters provided appears to be 183). The authors need to ensure scientific rigor and clarity by bringing more clarity in their sample size estimation process.

Response 9: Listing of stakeholders was done and used as the population to determine the sample size. Additional information as supplementary added.

Comment 10: The authors need to clarify where and why FGDs and KIIs were used?

From Table 1, it is unclear why certain stakeholders within the entire district are excluded when it might have been more convenient to include them. In this context, the authors should clarify what criteria define "convenience" in their use of convenient sampling.

Table 1 should have footnotes (for example ??F)

Response 10: FGDs and KIIs were carried out across the categories of respondents for the study.

Stakeholders not included in the districts are those at the national level and not available in the districts.

Comment 11: The authors can provide reference for “critical descriptive ethnography approach” for the benefit of readers.

Response 11: Reference for ‘critical descriptive ethnography approach’ is provided. Please, see revised manuscript Line 184.

Comment 12: From the description, it was not clear how quantitative data was collected and how sampling was done for quantitative component. What was the language of administration of quantitative questionnaire, its transnational validity, description of response categorisation as provided in table 2a and 2b etc.,

Response 12: Quantitative data was collected with the use of a questionnaire developed using Microsoft 365 Office form in which the questionnaire was self-administered or interviewer assisted by research data collection assistants recruited and trained for the purpose. The questionnaires were completed in the English Language or translated from the English Language into the local language of the respondent. See revised manuscript’s Lines 187-192

The manuscript’s text has been reviewed throughout and revisions made for clarity.

Comment 13: English language and grammar require revisions at places throughout the manuscript. For example, Line 213, “However. After data collection”

In line 219, the authors should clarify whether they are referring to "key indicators" or "themes." If it is "key indicators," they need to explain how these were derived.

Response 13: Key indicators’ in Line 198 of the manuscript, now Line 208 in the revised manuscript to ‘themes. These were key leadership and governance ingredients to realising integration of mental healthcare at the PHC as per the WHO handbook for monitoring the building blocks of health systems.

Comment 14: From the description it was not clear, whether some respondents were part of both qualitative and quantitative approach.

Response 14: A number of research respondents participated in both completing the questionnaire and in the key informant interviews and key informant interviews. This was particularly for the national level government officials and healthcare facility managers, leaders of SHGs, and disability/ human rights advocates. Please, see Lines 186 – 189.

Comment 15: The authors should briefly describe how and why data triangulation was done.

Response 15: Revised manuscript Lines 202 – 205 has additional text in response to the query which reads as “This was done by aligning the data to various categories and patterns in other to synthesise the information from the methods and data collection instruments utilised. Data triangulation helped to increase the validity and reliability of the data reducing or eliminating altogether bias and ensuring data saturation.”

Comment 16: Overall, the methodology seems weak, and the authors should provide more clarity and bring scientific rigor in describing their methodology to ensure the results are interpreted accurately.

Response 16: revisions have been made to improve the rigour of the methodology.

Comment 17: Results: The description of results from line 243-246 appear incongruent to the table 2a.

The table legend does not clearly distinguish between Table 2a, 2b, 3a, 3b. The authors should provide separate and more distinct legends for each table to enhance clarity. The column title for table 2b need to be revised as per the description in the main text (?? regions)

The authors should provide results/thematic illustration of the actor mapping exercise (mentioned in method section) either in the results section or as supplemental material.

The rationale for using a generic questionnaire for healthcare providers and service users, who have different mandates, responsibilities, competencies and needs, needs to be justified. The same applies to how the results are presented.

It is appreciable that the authors have discussed the relevance of results by geographic region in the discussion section.

The authors should supplement the quantitative questionnaire, KII and FGD guide for better validation and interpretation of the results.

Results could be benefited by providing sub-headings or key themes (for example it was not clear how oversight, coordination, and general stewardship were presented).

Response 17: Research study data collection and analysis framework Table provided as a supplementary.

The rationale for the generic questionnaire was so that

This has been done as presented in the results, Lines 315-623

The results section have been revised and have and sub-headings as in Lines 339, 448-449, 526, 555, and 623.

Comment 18: Discussion: With lack of adequate information in methods section, it was not clear whether “leadership and governance of mental health care at the community level” was assessed or “perceptions about leadership and governance of mental health care at the community level" was assessed.

In lines 503-505, the description does not clearly explain how the mention of traditional and spiritual healers' activities indicates that the importance of mental health is being gradually and steadily recognized. The authors should provide clearer explanation on this point.

The discussion of the qualitative findings is relatively limited compared to the quantitative findings, which raises concerns about the relevance and added value of the qualitative component in relation to the quantitative component.

The lines 521 to 525 which are politically sensitive, “The Minister of Health needs to make a strong case to get the President and Cabinet aware of the urgency of addressing the nation's mental health problems through a thorough implementation of current policies and laws supported by funding to expand access to mental healthcare. A presidential initiative on mental health might help the existing plan to build 111 hospitals in Ghana, including two psychiatric facilities” should be avoided. The authors should use technical terms and avoid terms referring to persons/personnel.

At places discussion appears not to flow from the results, for example lines 531-532, “high levels” of stigma and poor attitudes and practices towards mental health and people with mental neurological and substance use illnesses……,

Implications should also describe how findings from Ghana have global mental health relevance.

Limitations: one is not sure how identifying contextual issues on the leadership and governance in mental health services across the country is a limitation.

Response 18: The discussions section (Lines 641-696) has been revised to provide clarity. The results section has texts detailing respondent self-reported scores which inform the discussions.

The discussion section has been revised and adequately addresses the review comment(s) raised.

Same as above. The discussion section has been revised and address(es) the review comment raised.

The discussion section has been revised and the text referenced removed.

The discussion section has been revised and the text referenced removed.

This has been revised to read more appropriately

Comment 19: Limitations: one is not sure how identifying contextual issues on the leadership and governance in mental health services across the country is a limitation

Response 19: A section on ‘Generalisability, Limitations, and De-limitations’ has been revised and address(es) the review comment(s) raised. Please, see manuscript Lines 698-721.

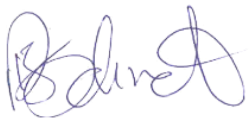

Peter Badimak YARO

E-mail:peter.yaro@basicneedsghana.org

Mobile Tel.: +233(0)244572733
