# Supplementary material for: Leadership and governance for integrating mental healthcare at the primary healthcare (PHC) level: A mixed methods study in Ghana
Source: PLOS Glob Public Health. 2025 Aug 19;5(8):e0002672. doi: 10.1371/journal.pgph.0002672 (PMC12364371; doi:10.1371/journal.pgph.0002672)
Supplement: Attachment — Submitted filename: YARO_Letter_response to Reviewer comments_07042025.pdf [file pgph.0002672.s008.pdf]

15th March 2025

Editors-in-Chief
PLOS Global Public Health
Journal on Health Policy and Systems Research

Dear Editors-in-Chief,

Resubmission of Revised Manuscript: Leadership and governance of mental healthcare and integration at the community level: a mixed methods study in Ghana (PGPH-D-23-02157R1)

I wish to resubmit the revised version of the above manuscript for review and consideration for publication in the PLOS Global Health Journal. The authors have addressed all the comments and provide a point-by-point response as follows:

Reviewer #2

Comment 1: The authors might consider using a more precise and technically appropriate term for "oversight" that complements better with the term "coordination." I presume the oversight the authors meant pertains to "supervision" or "monitoring"

Response 1: Reviewer's comment/ suggestion accepted and accordingly revised as in line(s) 24, 70, and 122 of the revised manuscript.

Comment 2: The figure title in the figure still label it as Figure 2.1 instead of Figure 1.

Response 2: Figure title and label is **Figure 1: Map of Ghana showing the study locations**. Please, refer to manuscript line 142 and also aligns with the supplementary information.

Based on Reviewer comment(s), the map was further enhanced with depictions of the ecological zones of the country which can be found in the supplementary information.

Comment 3: Regarding sampling terminology, the authors might reconsider using "stratified sampling" instead of "stratified purposive sampling." The term "purposive" is typically associated with non-probability sampling, and its usage should be reconsidered. Since the sampling units (respondents) are defined primarily by eligibility criteria, the terminology should accurately reflect the sampling approach.

Response 3: 'Stratified sampling' accordingly replaces 'stratified purposive sampling' to reflect the sampling approach used. Please, see, revised manuscript's line 158.

Comment 4: In the revised manuscript, the assumptions for sample size calculation have not been provided. Additionally, references 65–68 appear to be incorrectly placed. Without sufficient details, it is difficult to evaluate the veracity of the final estimate.

Response 4: The assumptions for sample size calculation have been provided in the supplementary information and detailed in the revised manuscript's lines 172 - 181. References 65-68 stated in the last revised manuscript have been appropriately addressed to the relevant texts which relate to sampling for the qualitative arm of the study's data collection, including aspects on maximum variation of respondents from the various stakeholder groups. The applicable reference (64) has been placed in the appropriate text. Please, see revised manuscript line 176.

Comment 5: The authors should incorporate their response -10 into the footnotes of Table 1. Furthermore, in Table 1, the meaning of "F" is unclear. While it might be inferred as "Female," the table should be self-explanatory to avoid ambiguity.

Response 5: Footnote added, and read as

[1]'F' is Female. FGDs and KIIs were carried out across the categories of respondents for the study. Stakeholders not included in the districts are those at the national level and not available in the districts.

Please, see revised manuscript page 12.

Comment 6: It is also unclear why certain paragraphs, such as "Ethics Approval and Permissions" and "Inclusivity in Global Research," were removed while some were added without explanation. These unexplained changes complicate the review process, making it challenging to assess the revisions without starting from scratch.

Response 6: A section on 'Ethics Approval and Permissions and Inclusivity in Global Research' is added in the revised manuscript from lines 289 – 298.

Comment 7: There are quite lot of discrepancies (with additions, deletions and modifications) between the previous and revised versions particularly in the methodology section that raises serious concerns. To improve clarity, the authors should explicitly mention which paragraphs or lines were added or deleted and provide a justification for these changes.

Response 7: Point-by-point explanations of revisions made to the last revision (of 13th January 2025) and the current (7th April, 2025) revision on the manuscript are detailed in responses 7.1 to 7.16.

Response 7.1: Revised manuscript line 2 – Portions of titled changed at the recommendation of Reviewer #2. '*... for integrating mental healthcare ...*' replaced '*... of mental healthcare and integration ...*', and '*... primary healthcare (PHC) ...*' replaced '*... community ...*'.

The authors agreed with the suggestion of Reviewer #2 to refine the phrase and replace the word/ term 'Community' for to ensure the title aligns with the focus of the paper.

Response 7.2: Revised manuscript line(s) 21, 23, – For alignment and consistency with the suggestion of Reviewer #2, '*Community*' was replaced with '*Primary Health Care (PHC)*'.

Response 7.3: Revised manuscript line 119. The word '*undertakings*' replaced '*indicators*' as the focus is one actions that demonstrate towards realising integration of mental healthcare at the PHC level.

Response 7.4: Revised manuscript lines 121 - 122, - the phrase '*... structures put ...*' was deleted for the sentence to read as '*... to assess the leadership and governance in place for the integration of mental healthcare at the PHC in Ghana.*' rather than '*... to assess the leadership and governance structures put in place to ensure oversight ...*'. This is so as to focus on the dynamics interactions and actions that demonstrate a sense of integrated mental healthcare services at the PHC level than use of the word 'structures.'

Lines 122 - 123 is an additional sentence, '*This is part of understanding the oversight, coordination and general stewardship of mental healthcare integration in Ghana.*' to provide clarity on the leadership and governance of mental healthcare integration at the PHC.

In this current response, the sentence has been further revised with the word '*oversight*' replaced with '*supervision and monitoring*' agreeing with advice and recommendation of Reviewer #2 to use precise and technically appropriate term.

Response 7.5: Revised manuscript lines 125 - 136 on **Study design** was revised by being expanded upon to address Reviewer #2 comment(s) requesting that clarity be provided on how quantitative data was collected and how sampling was done for the quantitative component, as well as the language of administration of quantitative questionnaire, its transnational validity, description of respondent categorisation as provided in the results tables.

Response 7.6: Revised manuscript lines 138 - 150 on **Study area** was improved to clarify the healthcare service referrals tiers of Ghana. The revision was in response to Reviewer #2 comments.

Response 7.7: Revision was done on the Section on '**Sampling and Sample size determination'** based on Reviewer #2 comments on that that section needed further clarification on sample size estimation and sample size calculation, and the population of the target districts and how they were determined. The additional texts are from lines 157 – 181. These

The manuscript's lines 183 – 231 provided clarity on the qualitative arm of the data collection. These covered explanations for the terms 'purposive sampling' (lines 183-187), 'maximum variation' (lines 188-196), 'categorisation of stakeholders and selection of respondents' (lines 190 – 227), and 'data saturation' (lines 229 – 231).

Response 7.8: Additional text was added to the section on **Data collection** (lines 229 – 233) to describe how the questionnaire for the quantitative data was designed Based on reviewer comments, additional text was provided on data triangulation.

Additional text was also provided to give clarity on data reliability and validity (manuscript lines 240 – 249) detailing the pre-test of the questionnaire for finalisation before the field level collection of the quantitative data.

Response 7.9: The section on **Data collection procedure** (lines 256 – 275) was revised to add clarity to how the questionnaire was administered and the languages used in the data collection.

Response 7.10: Additional text was provided on **'data triangulation'** to clarify how and why data triangulation was carried out in the study. This was one of the comments Reviewer #2 commented on requesting that it be addressed. Please, see revised manuscript's lines 276 – 280.

Response 7.11: A sentence was added to detail how '**reflexivity**' was ensured in the study. Please, see manuscript's lines 281 – 284.

Response 7.12: Additional texts were added in the study's data '**Analysis**' section to explain how qualitative data was collected and kept, transcribed, and analysed, with the use of Thematic Analysis approach. Please, see revised manuscripts lines 299 – 218.

Additional texts were provided to explain overall means scores of study respondents' self-reported perceptions of level(s) of integration of mental health in each of the health systems building block. Please, see revised manuscript's lines 219 – 646. Tables of these data are under each of the sub-headings of the study's **Results section.** These were not included in the previous manuscript.

Response 7.13: The entire '**Results**' section was revised for clarity and based on Reviewer #2 comments that the manuscript's results description needed to properly label the results tables with "… separate and more distinct legends for each table to enhance clarity", address typographical and grammatical errors and to have "… sub-headings or key themes".

The 'Results' section have introductory paragraphs (lines 321 – 343), and four sub-sections and a conclusion (345 – 646), The sub-headings are *Level of awareness and implementation of mental health legislation, policy and plan* (manuscript lines 241 – 452), *Presence of focal persons for mental health at the PHC and level of involvement of health workers in mental health at the community level* (manuscript lines 455 – 525), *Private sector involvement in mental healthcare at the PHC level* (manuscript lines 532 – 558), *Monitoring and Evaluation of mental healthcare at the PHC* (manuscript lines 561 – 626) and a concluding section on *Overall perception of the level of leadership and governance at the PHC* (manuscript lines 629 – 646).

Response 7.14: The **Discussion** section was also accordingly revised affording revision of sentences, typological and grammatical issues needing attention. Please, see revised manuscript lines 647 –701.

Response 7.15: The **Generalisability, limitations, de-limitation** revised spelling and sentence constructions. Please, see revised manuscript's lines 703 – 726.

Response 7.16: The **Conclusion** section was also revised for clarity. Please, see revised manuscript's lines 728 – 759.

Comment 8: Finally, the lack of a point-by-point response and the use of a generic response by combining the queries makes it difficult to determine whether the concerns have been adequately addressed for the benefit of the readers. Providing a detailed point-by-point response would enhance transparency and facilitate the review process.

Response 8: Responses 7 to 7.16 above provide point-by-point details to every revision made on the last manuscript and the current one.

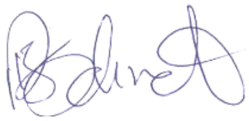

Peter Badimak YARO

E-mail:peter.yaro@basicneedsghana.org

Mobile Tel.: +233(0)244572733
