# Supplementary material for: Leadership and governance for integrating mental healthcare at the primary healthcare (PHC) level: A mixed methods study in Ghana
Source: PLOS Glob Public Health. 2025 Aug 19;5(8):e0002672. doi: 10.1371/journal.pgph.0002672 (PMC12364371; doi:10.1371/journal.pgph.0002672)
Supplement: Attachment — Submitted filename: YARO_Letter_response to Reviewer comments_05072025.pdf [file pgph.0002672.s009.pdf]

5th July 2025

Editors-in-Chief
PLOS Global Public Health
Journal on Health Policy and Systems Research

Dear Editors-in-Chief,

Resubmission of Revised Manuscript: Leadership and governance of mental healthcare and integration at the community level: a mixed methods study in Ghana (PGPH-D-23-02157R3)

I wish to resubmit the revised version of the above manuscript for review and consideration for publication in the PLOS Global Health Journal.

Thank you to **Reviewer #2 and Dr Abiona Taiwo Olufemi (Reviewer #4)** for the valuable feedback.

The authors have addressed all the comments and provide a point-by-point response as follows:

Reviewer #2

Comment 1: The authors have addressed most of the concerns diligently. Before accepting, the authors can recheck the lines 172-174 and to confirm whether it is "0.00115403 proportion of the population" or 0.115403% of the population". With the current description of 0.115403 proportion of the population, the sample comes to 110394 instead of 1104.

Response 1: Reviewer's comment/ suggestion accepted and accordingly revised as in line 173 of the revised manuscript.

Reviewer #4

Comment 1: Abstract
The author indicated that the study is a mixed method; hence, the author should add a key major finding from the quantitative analysis to the abstract..

Response 1: In line 32 of the revised manuscript, "composite mean score of 2.53", indicating low level of development of leadership and governance at the community level.

Comment 2: Introduction
The author should rephrase the first sentence in the introduction section. The phrase "mental health conditions" appears three times, making the sentence wordy. This could be rephrased as: "Only a small proportion of people with mental health conditions receive treatment, despite effective interventions that address these conditions, prevent associated impact, reduce their prevalence, and promote overall mental wellbeing."

Response 2: Reviewer's suggested statement "Only a small proportion of people with mental health conditions receive treatment, despite effective interventions that address these conditions, prevent associated impact, reduce their prevalence, and promote overall mental wellbeing' has been accepted and accordingly revised in the revised manuscript lines 43-47.

Comment 3: Line 130: add a comma after the word "approaches".

Response 3: Comma added appropriately in line 132 as per reviewer's recommendation of the revised manuscript.

Comment 4: Line 176: remove the website link since "(64)" indicates this.

Response 4: The website link has been revised as advised. Please, line 178 of revised manuscript.

Comment 5: Lines 433-436: The structure of the statement makes it difficult to see the comparison between groups. The author needs more clarity and flow with a logical structure. The phrase "by the location of the respondents was partial, 41.9% (348)" is unclear. What was the percentage representing? Could it be that the author meant that, overall, 41.9% (348) of the respondents had partial awareness of Ghana's mental health policy implementation at the PHC level? If that is the case, it will contradict the next statement, "This was followed by 35.7% (296) of respondents of the three geographical locations". The author should use a more concise and clear reporting method used in academic journal..

Response 5: Reviewer comments and suggestion(s) are noted and have informed the revisions made in lines 435-439 as follows:

"Overall, awareness of Ghana's mental health policy and its implementation at the PHC level was partial, (41.9% (348)) across the three geographical zones, followed by 35.7% (296) of respondents indicating low or non-implementation, with less than a quarter (22.4% (186)) of the view that there was a high level of awareness and implementation of the mental health policy at the PHC level.".

Comment 6: Line 575: Add a comma after the word "low".

Response 6: Comma added after "low" as per Reviewer recommendation in line 578 of revised manuscript.

Comment 7: Line 638: add bracket open in front 2.43..

Response 7: Bracket open added in front of 2.43. Please, see line 641 of revised manuscript.

Comment 8: Line 674: Change the capital letter "L" in the word "Law" to a small letter l.

Response 8: The capital letter "L" in the word "Law" changed to a small letter l. Kindly, see revised manuscript line 677.

Sincerely,

Peter Badimak YARO

E-mail:peter.yaro@basicneedsghana.org

Mobile Tel.: +233(0)244572733
